# Supplementary material for: Motifs in Natural Products as Useful Scaffolds to Obtain Novel Benzo[d]imidazole-Based Cannabinoid Type 2 (CB2) Receptor Agonists
Source: Int J Mol Sci. 2023 Jun 30;24(13):10918. doi: 10.3390/ijms241310918 (PMC10341893; doi:10.3390/ijms241310918)
Supplement: Supplementary file 1 [file ijms-24-10918-s001.zip › ijms-2334203-supplementary.pdf]

Motifs in natural products as useful scaffolds to obtain novel benzo[*d*]imidazole-based cannabinoid type 2 (CB2) receptor agonists

Analia Young Hwa Cho[1], Hery Chung[1], Javier Romero-Parra[2], Poulami Kumar[3], Marco Allarà[3], Alessia Ligresti[3], Carlos Gallardo-Garrido[1], Hernán Pessoa-Mahana[2], Mario Faúndez[1], Carlos David Pessoa-Mahana[1,*]

[1] Pharmacy Department, Faculty of Chemistry, Pontificia Universidad Católica de Chile, Vicuña Mackenna 4860, Santiago 7820436, Chile; cpessoa@uc.cl

[2] Organic Chemistry and Physical Chemistry Department, Faculty of Chemical and Pharmaceutical Sciences, Universidad de Chile, Olivos 1007, Santiago 7820436, Chile

[3] National Research Council of Italy, Institute of Biomolecular Chemistry, 80078 Pozzuoli, Italy

* Correspondence: cpessoa@uc.cl

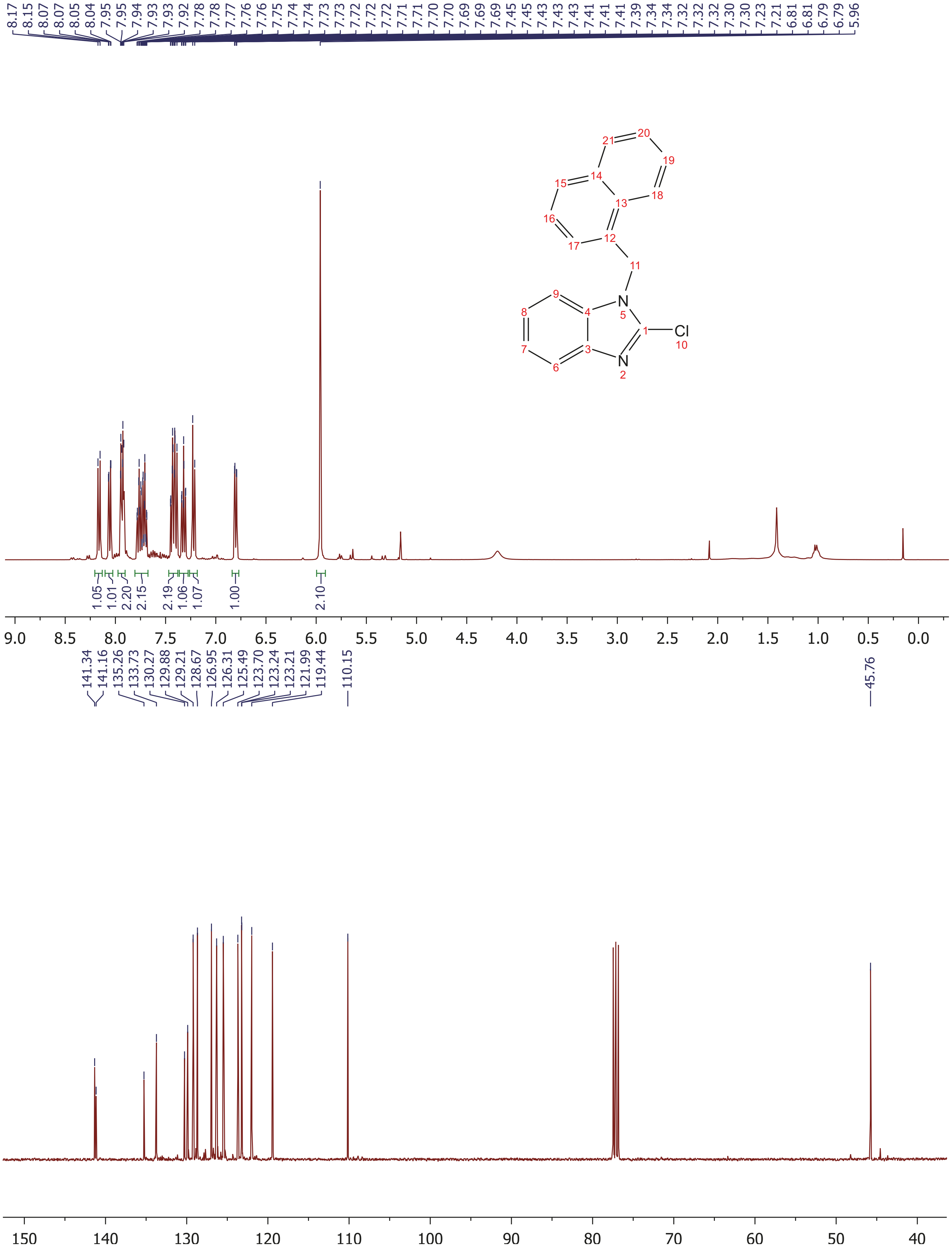
8.17 8.15 8.07 8.07 8.05 8.04 7.95 7.95 7.94 7.93 7.93 7.92 7.78 7.78 7.77 7.76 7.76 7.75 7.74 7.74 7.73 7.73 7.72 7.72 7.72 7.71 7.71 7.70 7.70 7.69 7.69 7.69 7.45 7.43 7.43 7.43 7.41 7.41 7.41 7.39 7.34 7.34 7.32 7.32 7.30 7.30 7.23 7.21 6.81 6.81 6.79 6.79 5.96
N
N
Cl
1.05 1.01 2.20 2.15 2.19 1.06 1.07 1.00 2.10
9.0 8.5 8.0 7.5 7.0 6.5 6.0 5.5 5.0 4.5 4.0 3.5 3.0 2.5 2.0 1.5 1.0 0.5 0.0
141.34 141.16 135.26 133.73 130.27 129.88 129.21 128.67 126.95 126.31 125.49 123.70 123.24 123.21 121.99 119.44 110.15 45.76
150 140 130 120 110 100 90 80 70 60 50 40

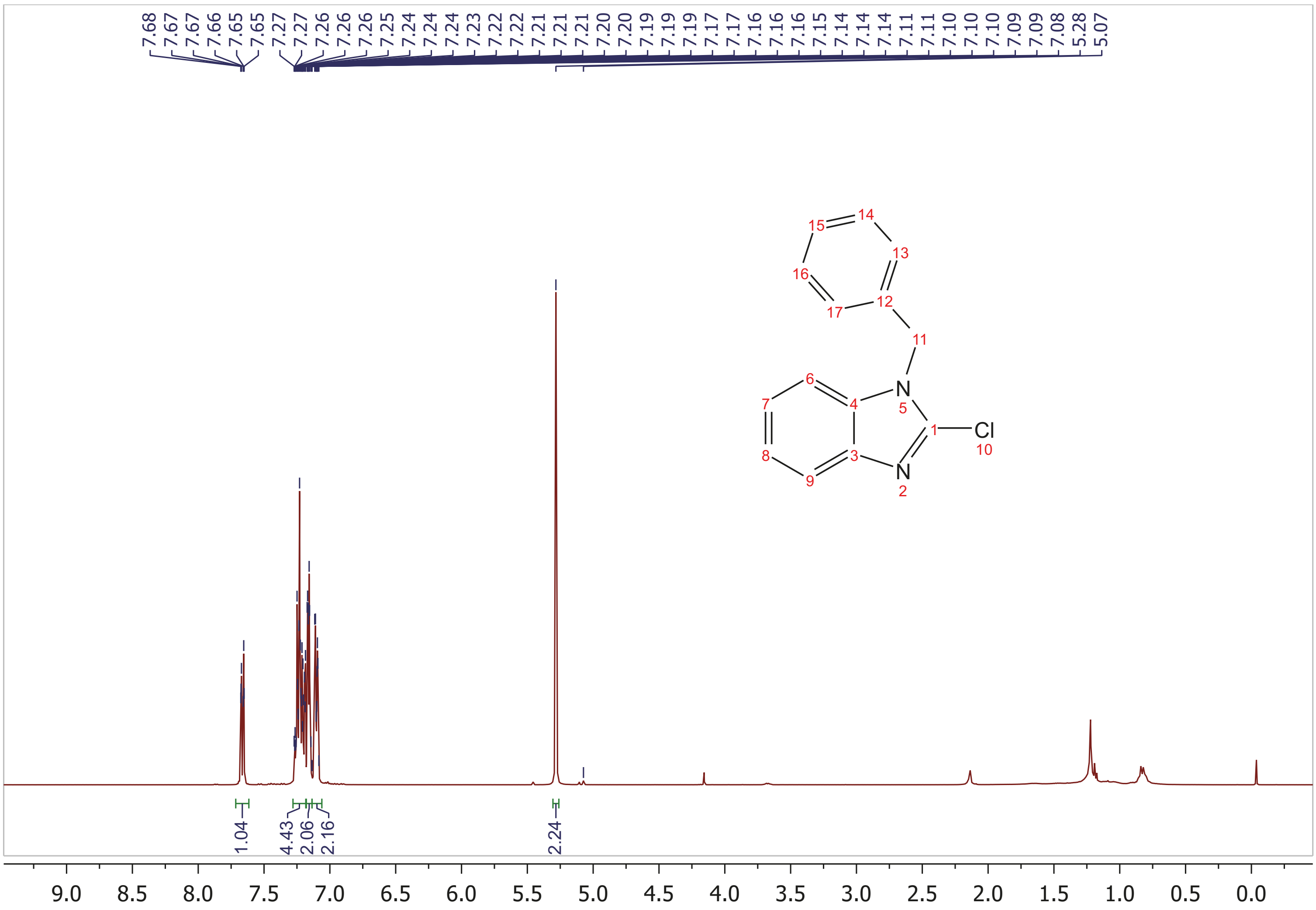
7.68
7.67
7.67
7.66
7.65
7.65
7.27
7.27
7.26
7.26
7.26
7.25
7.24
7.24
7.24
7.23
7.22
7.22
7.21
7.21
7.21
7.20
7.20
7.19
7.19
7.19
7.17
7.17
7.16
7.16
7.15
7.14
7.14
7.14
7.11
7.11
7.10
7.10
7.10
7.09
7.09
7.08
5.28
5.07
Cl
N
N
1.04
4.43
2.06
2.16
2.24
9.0
8.5
8.0
7.5
7.0
6.5
6.0
5.5
5.0
4.5
4.0
3.5
3.0
2.5
2.0
1.5
1.0
0.5
0.0

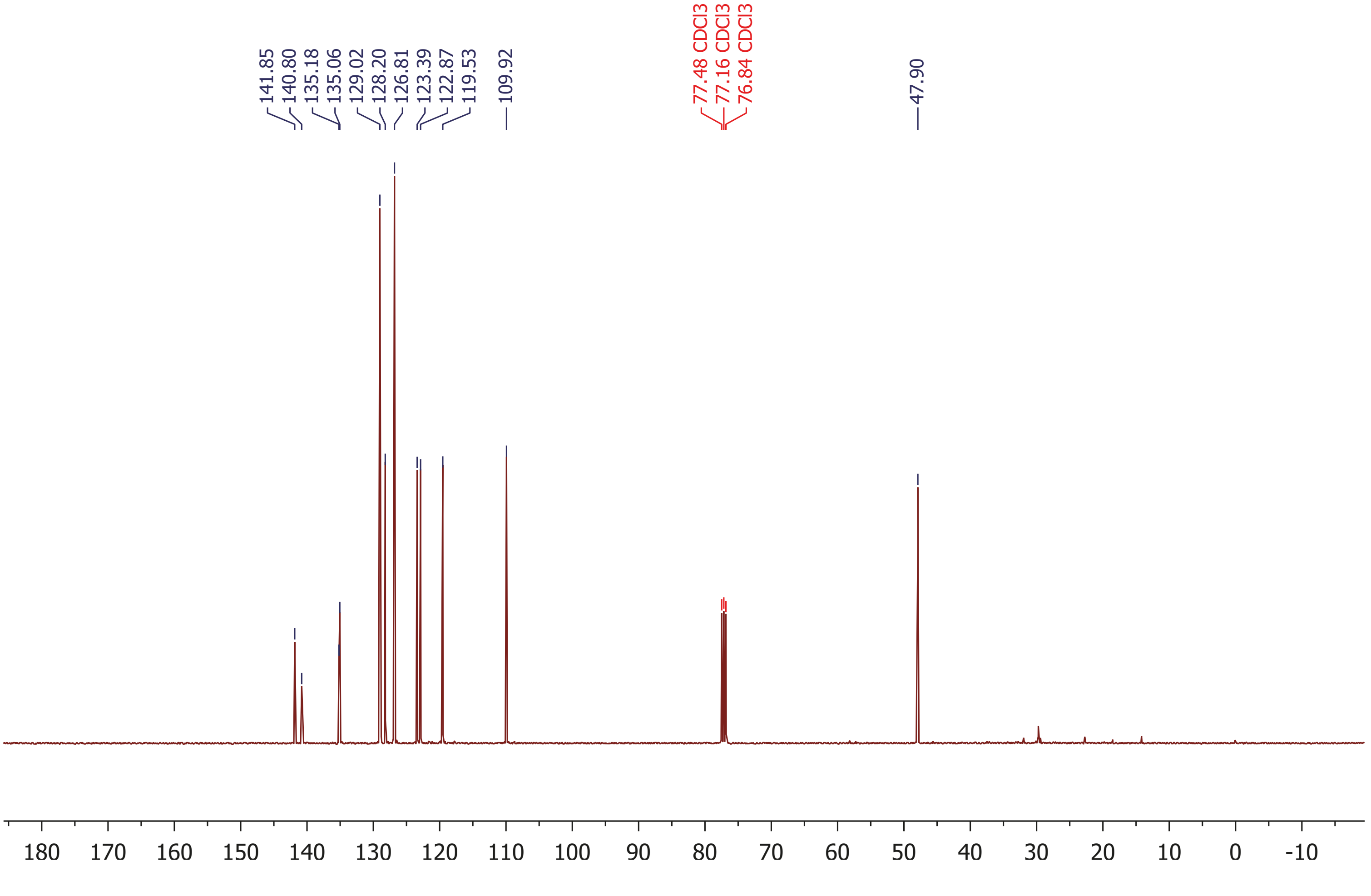
141.85
140.80
135.18
135.06
129.02
128.20
126.81
123.39
122.87
119.53
109.92
77.48 CDCl3
77.16 CDCl3
76.84 CDCl3
47.90
180
170
160
150
140
130
120
110
100
90
80
70
60
50
40
30
20
10
0
-10

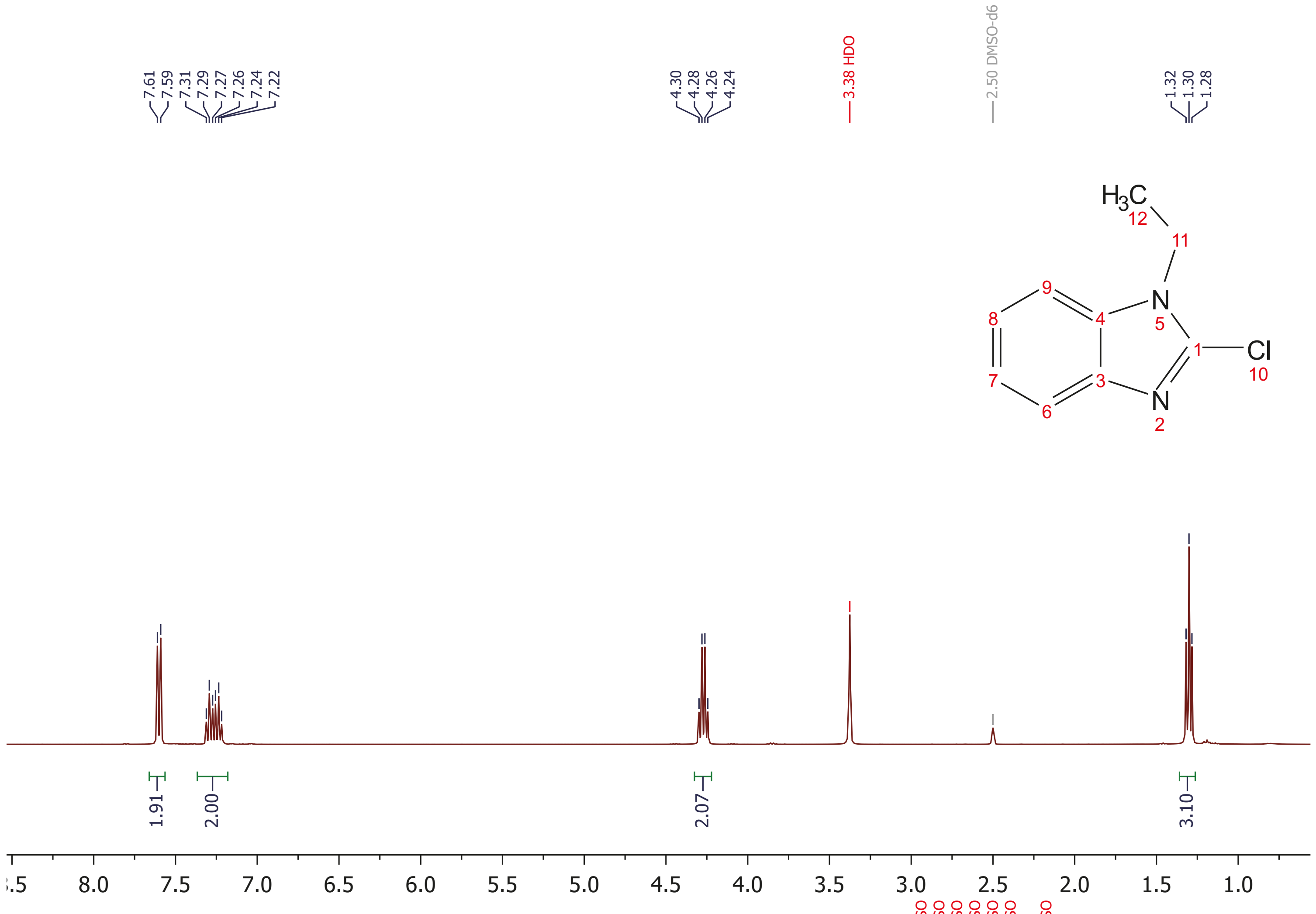
7.61
7.59
7.31
7.29
7.27
7.26
7.24
7.22
4.30
4.28
4.26
4.24
3.38 HDO
2.50 DMSO-d6
1.32
1.30
1.28
H3C
12
11
9
8
7
6
4
3
N
5
N
2
1
Cl
10
1.91
2.00
2.07
3.10
8.0
7.5
7.0
6.5
6.0
5.5
5.0
4.5
4.0
3.5
3.0
2.5
2.0
1.5
1.0

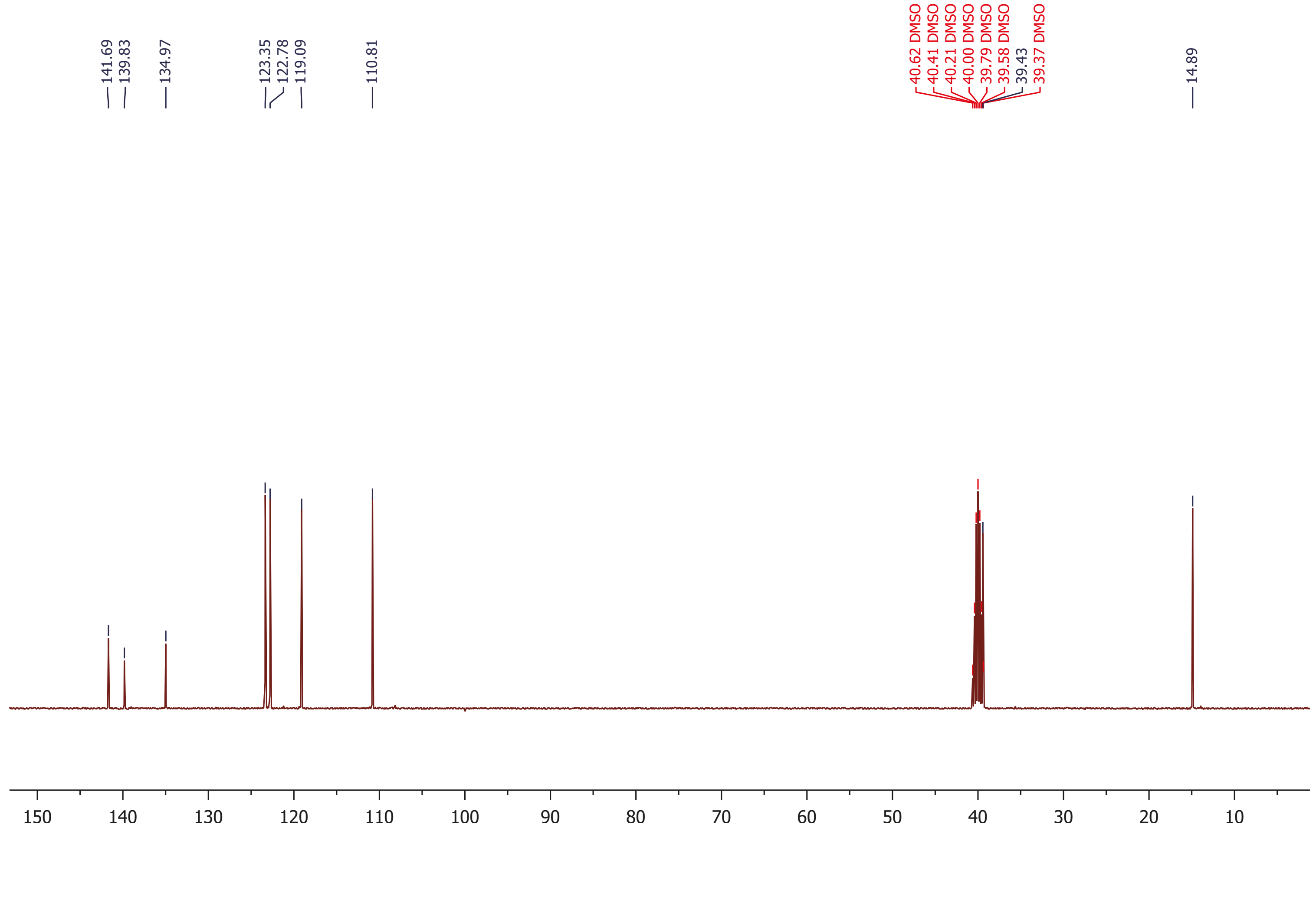
141.69
139.83
134.97
123.35
122.78
119.09
110.81
40.62 DMSO
40.41 DMSO
40.21 DMSO
40.00 DMSO
39.79 DMSO
39.58 DMSO
39.43
39.37 DMSO
14.89
150
140
130
120
110
100
90
80
70
60
50
40
30
20
10

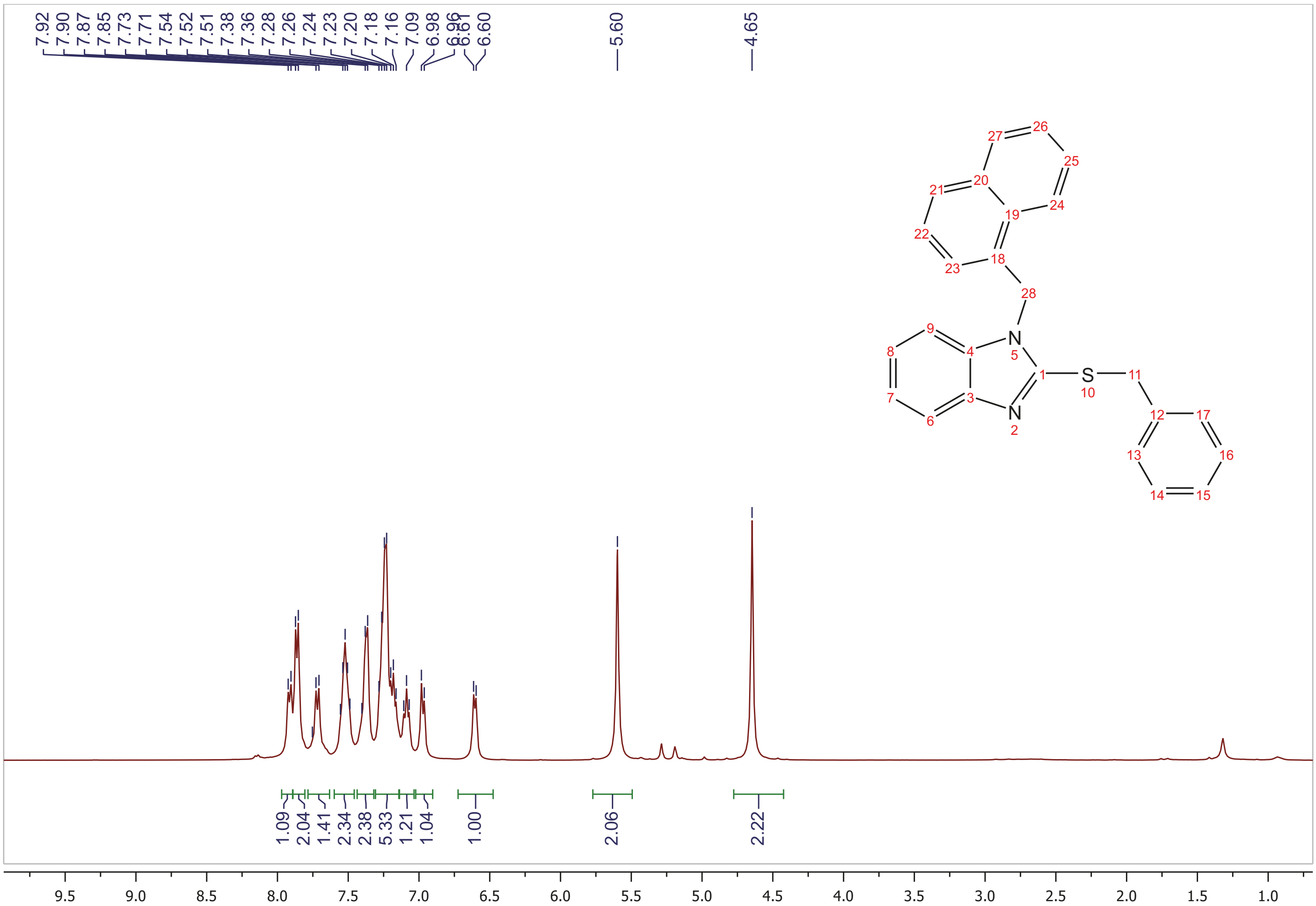
7.92
7.90
7.87
7.85
7.73
7.71
7.54
7.52
7.51
7.38
7.36
7.28
7.26
7.24
7.23
7.20
7.18
7.16
7.09
6.98
6.96
6.61
6.60
5.60
4.65
1.09
2.04
1.41
2.34
2.38
5.33
1.21
1.04
1.00
2.06
2.22
9.5
9.0
8.5
8.0
7.5
7.0
6.5
6.0
5.5
5.0
4.5
4.0
3.5
3.0
2.5
2.0
1.5
1.0

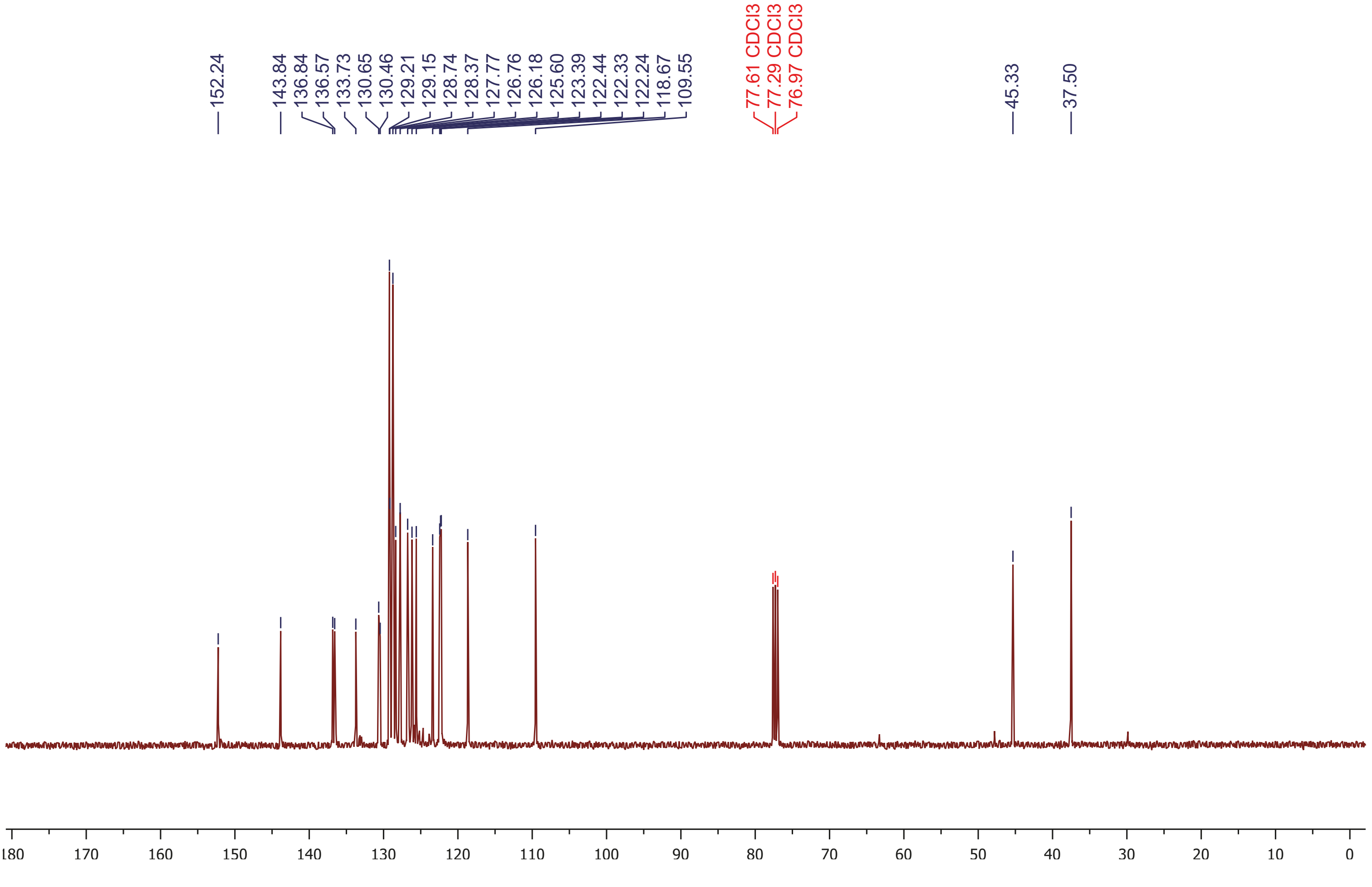
152.24
143.84
136.84
136.57
133.73
130.65
130.46
129.21
129.15
128.74
128.37
127.77
126.76
126.18
125.60
123.39
122.44
122.33
122.24
118.67
109.55
77.61 CDCl3
77.29 CDCl3
76.97 CDCl3
45.33
37.50
180
170
160
150
140
130
120
110
100
90
80
70
60
50
40
30
20
10
0

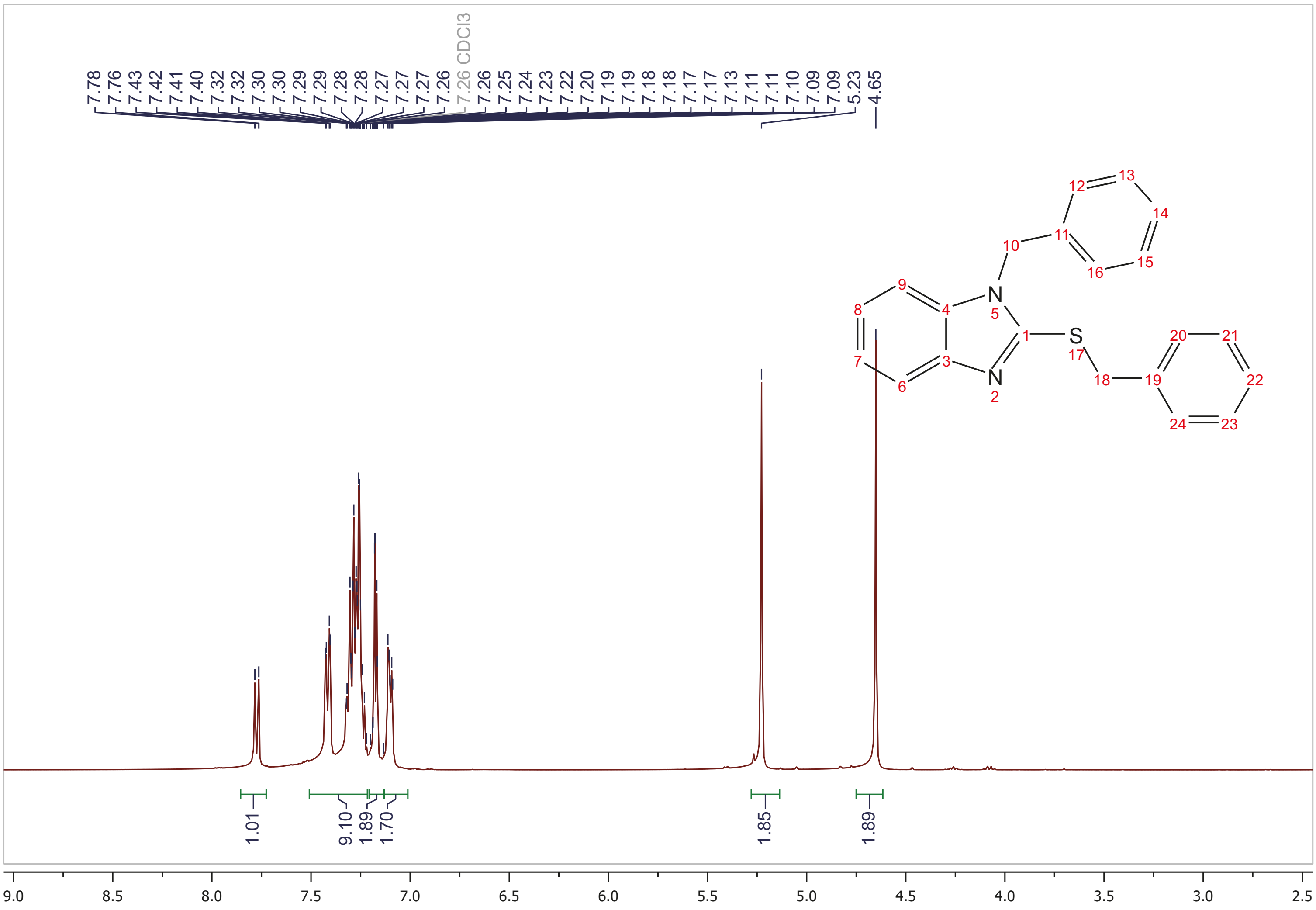

7.78
7.76
7.43
7.42
7.41
7.40
7.32
7.32
7.30
7.30
7.29
7.29
7.28
7.28
7.27
7.27
7.27
7.26
7.26 CDCl3
7.26
7.25
7.24
7.23
7.22
7.20
7.19
7.19
7.18
7.18
7.17
7.17
7.13
7.11
7.11
7.10
7.09
7.09
5.23
4.65
1.01
9.10
1.89
1.70
1.85
1.89
9.0
8.5
8.0
7.5
7.0
6.5
6.0
5.5
5.0
4.5
4.0
3.5
3.0
2.5
N
S
N
1
2
3
4
5
6
7
8
9
10
11
12
13
14
15
16
17
18
19
20
21
22
23
24

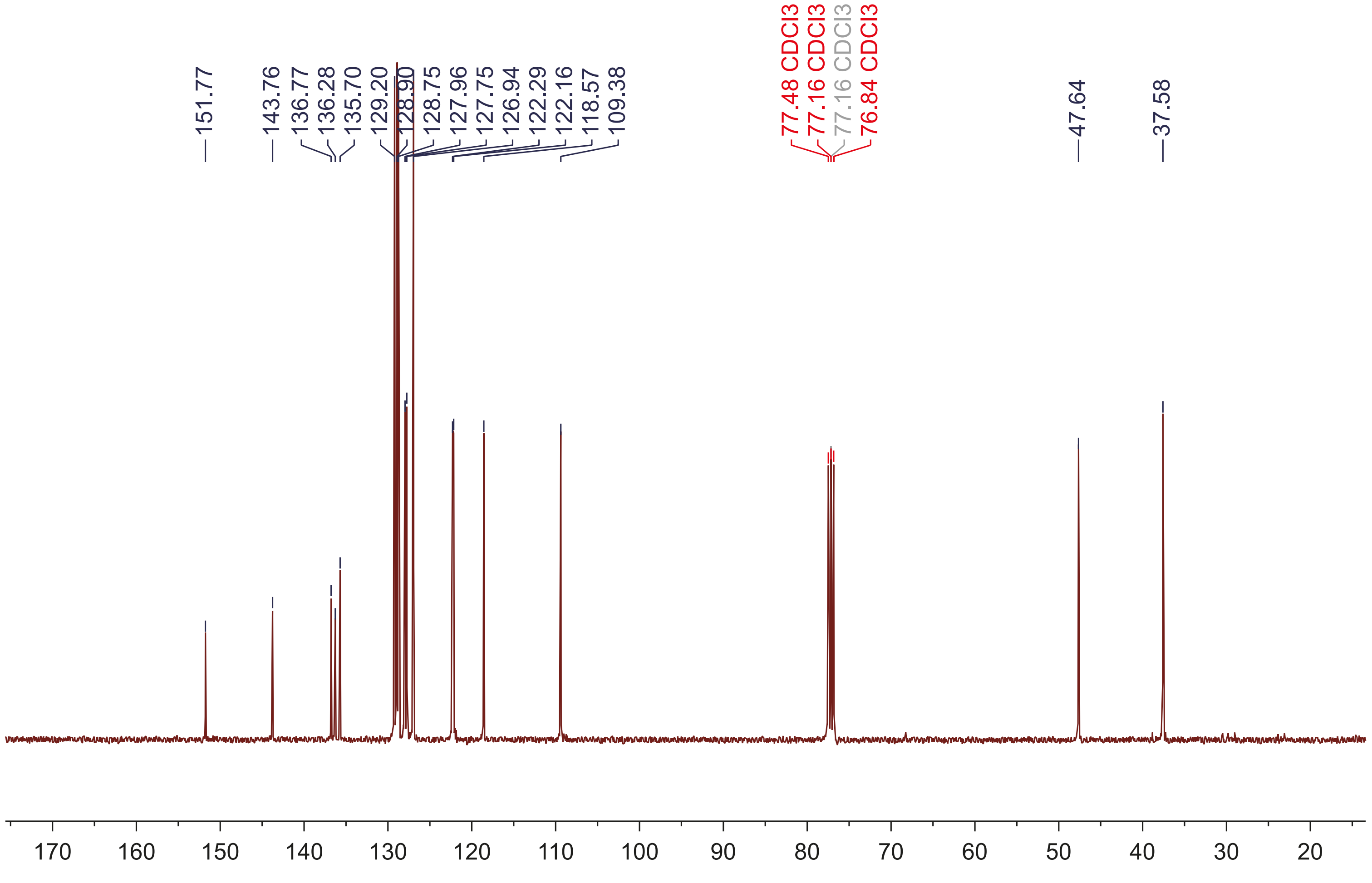

151.77
143.76
136.77
136.28
135.70
129.20
128.90
128.75
127.96
127.75
126.94
122.29
122.16
118.57
109.38
77.48 CDCl3
77.16 CDCl3
77.16 CDCl3
76.84 CDCl3
47.64
37.58
170
160
150
140
130
120
110
100
90
80
70
60
50
40
30
20

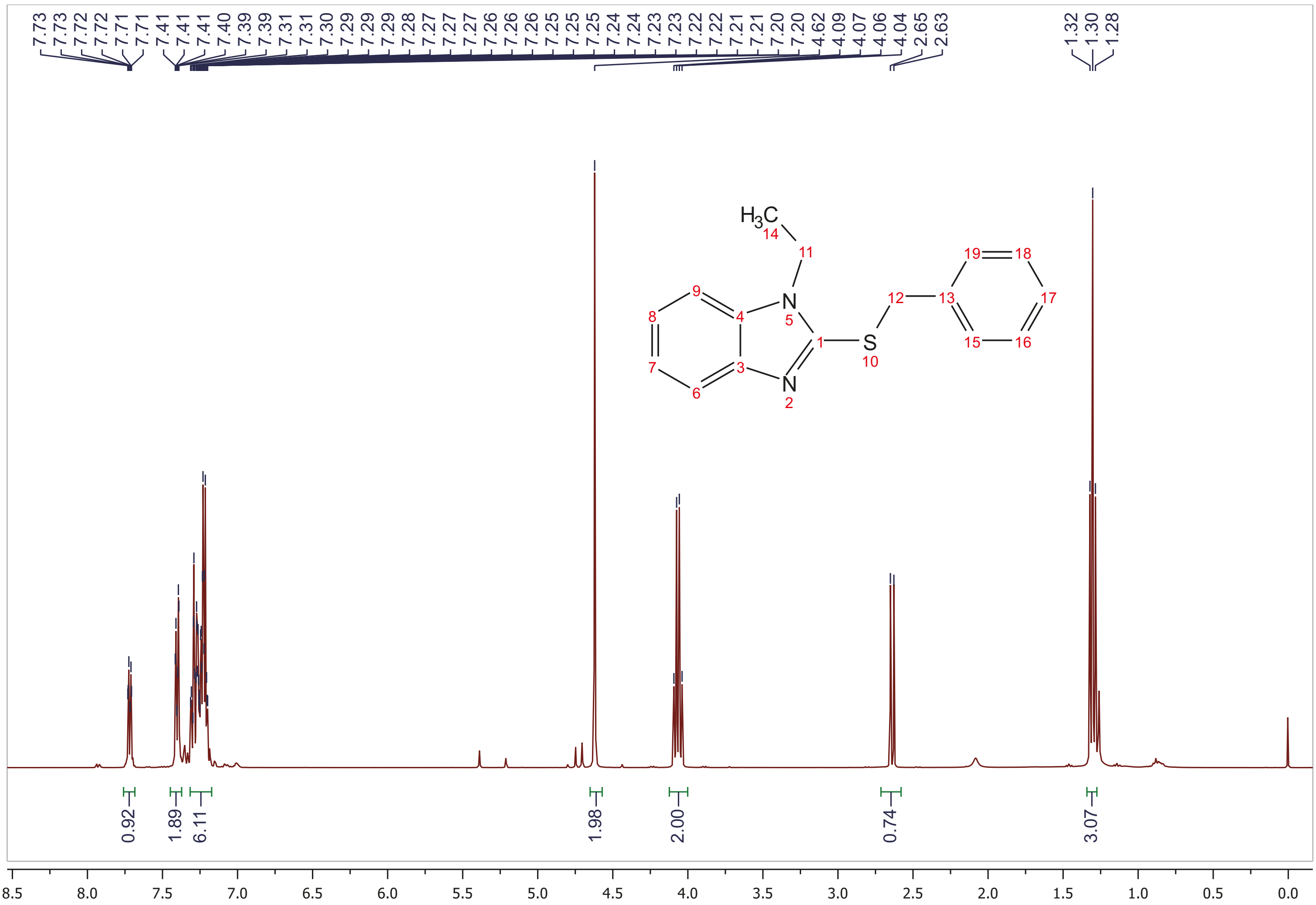

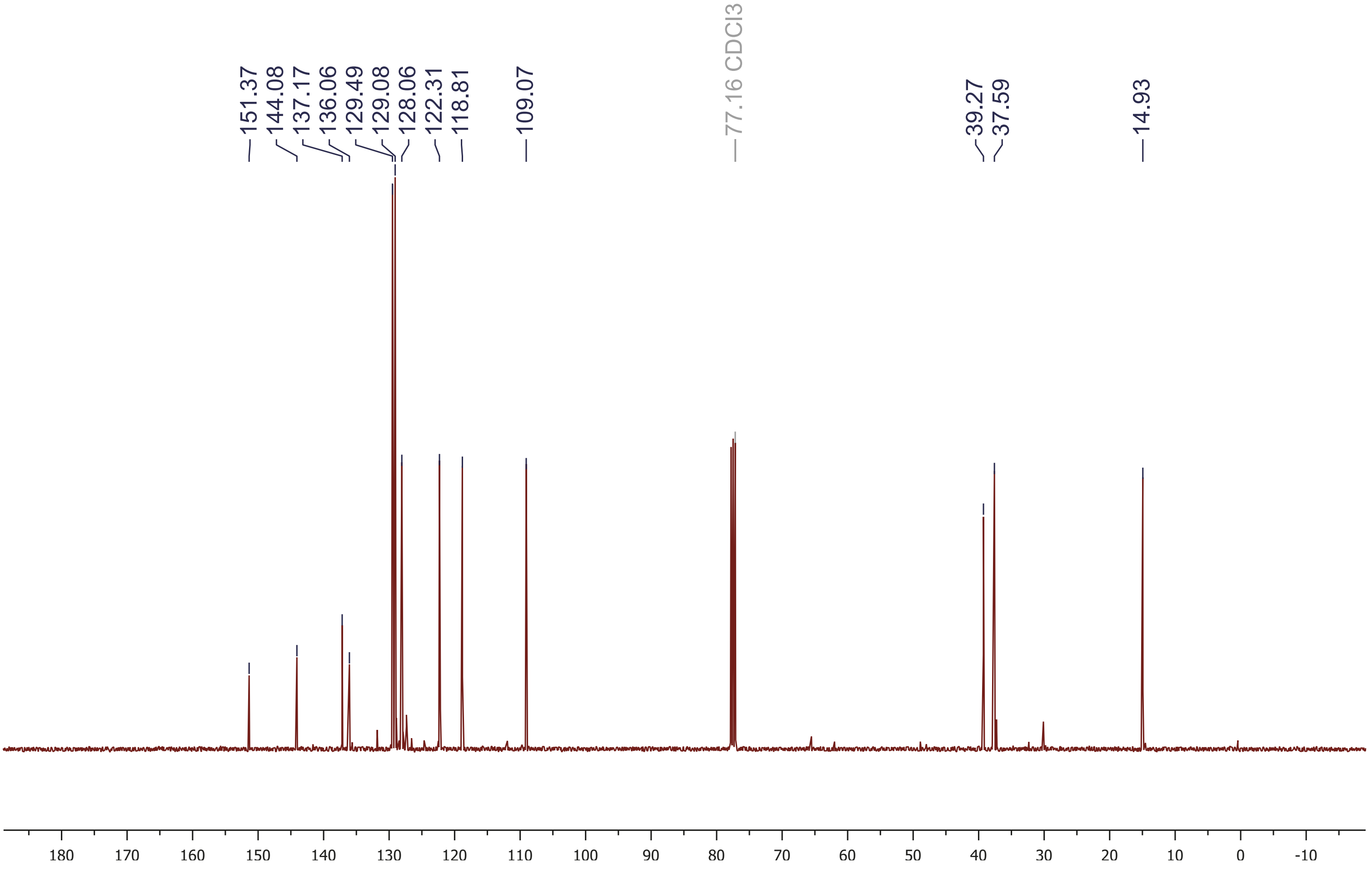

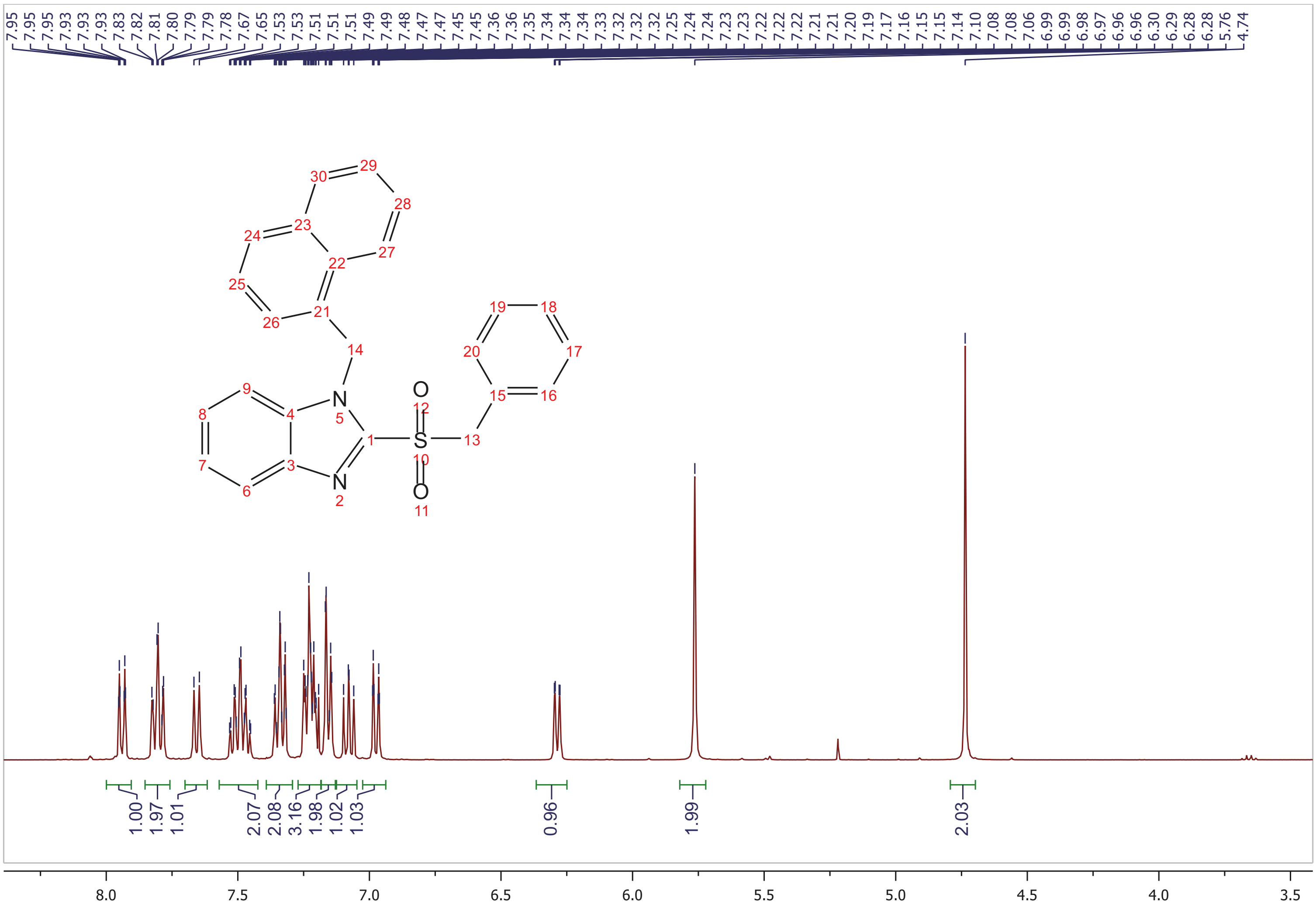
7.95 7.95 7.93 7.93 7.93 7.83 7.82 7.81 7.80 7.79 7.79 7.78 7.67 7.65 7.53 7.53 7.51 7.51 7.51 7.49 7.49 7.48 7.47 7.45 7.45 7.36 7.36 7.35 7.34 7.34 7.34 7.33 7.32 7.32 7.25 7.24 7.24 7.23 7.23 7.22 7.22 7.22 7.21 7.21 7.20 7.19 7.17 7.16 7.15 7.15 7.14 7.10 7.08 7.08 7.06 6.99 6.98 6.97 6.96 6.96 6.30 6.29 6.28 6.28 5.76 4.74
N
N
S
O
O
1.00 1.97 1.01 2.07 2.08 3.16 1.98 1.02 1.03 0.96 1.99 2.03
8.0 7.5 7.0 6.5 6.0 5.5 5.0 4.5 4.0 3.5

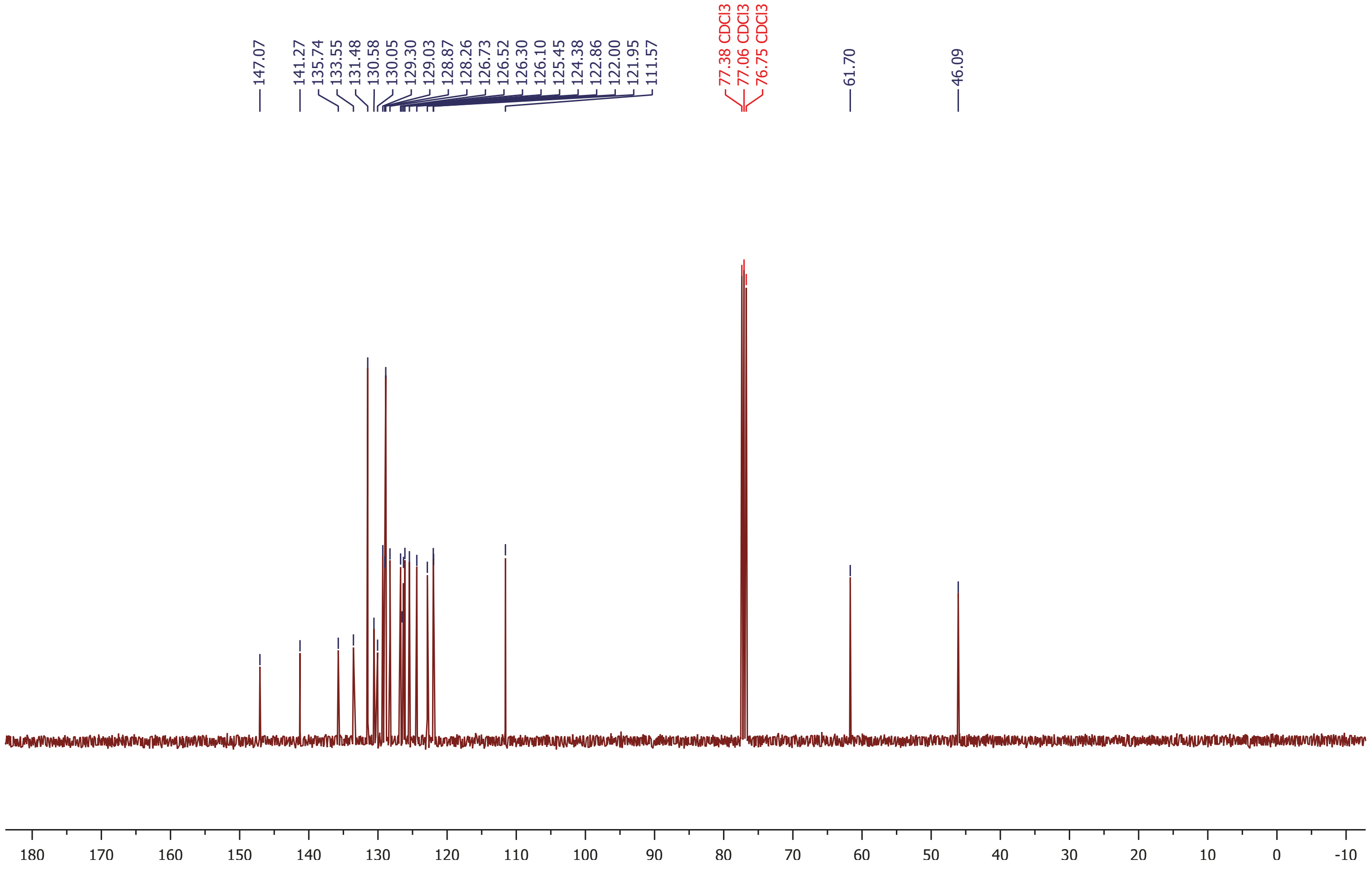
147.07 141.27 135.74 133.55 131.48 130.58 130.05 129.30 129.03 128.87 128.26 126.73 126.52 126.30 126.10 125.45 124.38 122.86 122.00 121.95 111.57
77.38 CDCl3
77.06 CDCl3
76.75 CDCl3
61.70
46.09
180 170 160 150 140 130 120 110 100 90 80 70 60 50 40 30 20 10 0 -10

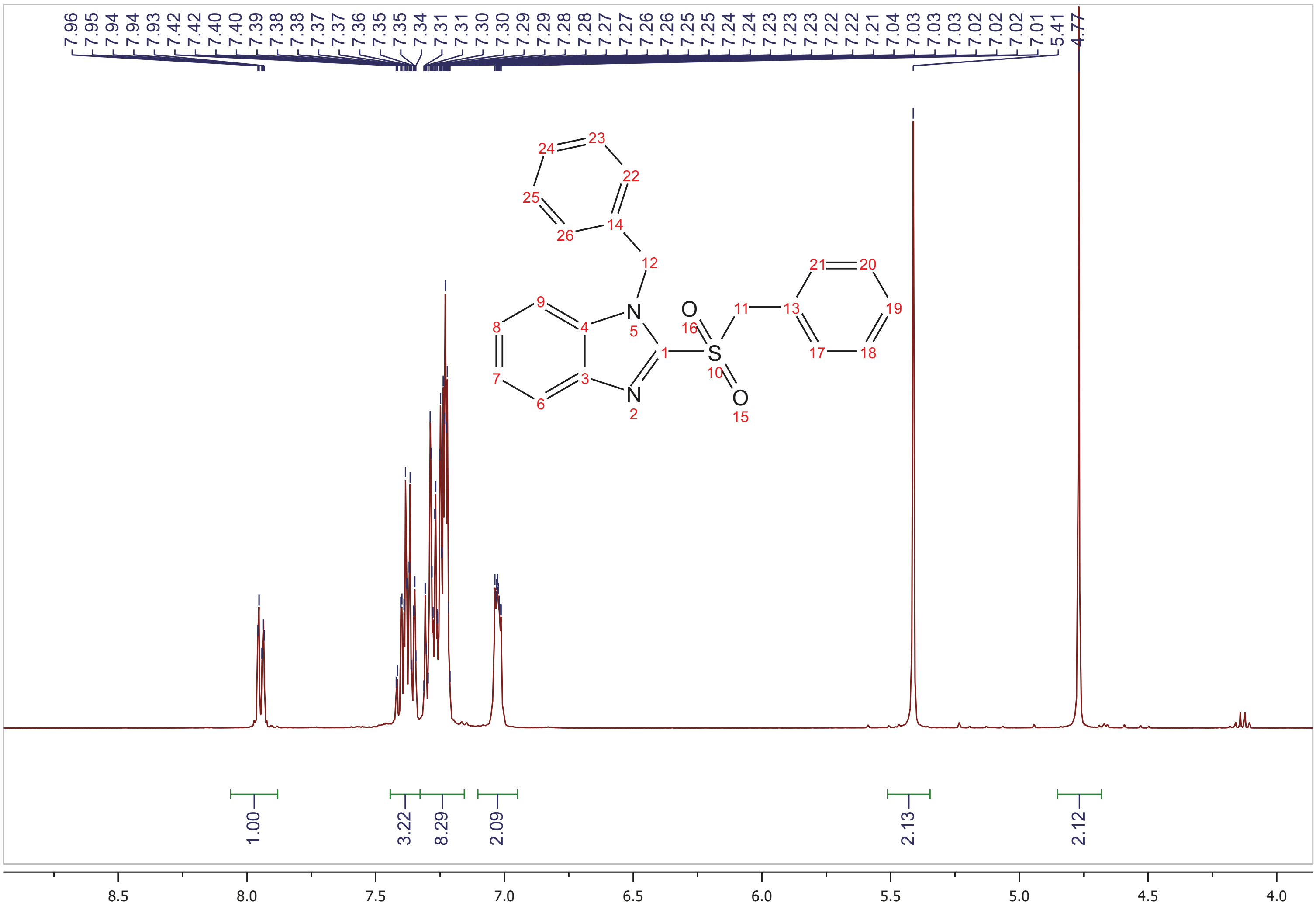
7.96 7.95 7.94 7.94 7.93 7.42 7.42 7.40 7.40 7.39 7.38 7.38 7.37 7.37 7.36 7.35 7.35 7.34 7.31 7.31 7.30 7.30 7.29 7.29 7.28 7.28 7.27 7.27 7.26 7.26 7.25 7.25 7.24 7.24 7.23 7.23 7.23 7.22 7.22 7.21 7.04 7.03 7.03 7.03 7.02 7.02 7.02 7.01 5.41 4.77
1.00
3.22
8.29
2.09
2.13
2.12
8.5
8.0
7.5
7.0
6.5
6.0
5.5
5.0
4.5
4.0

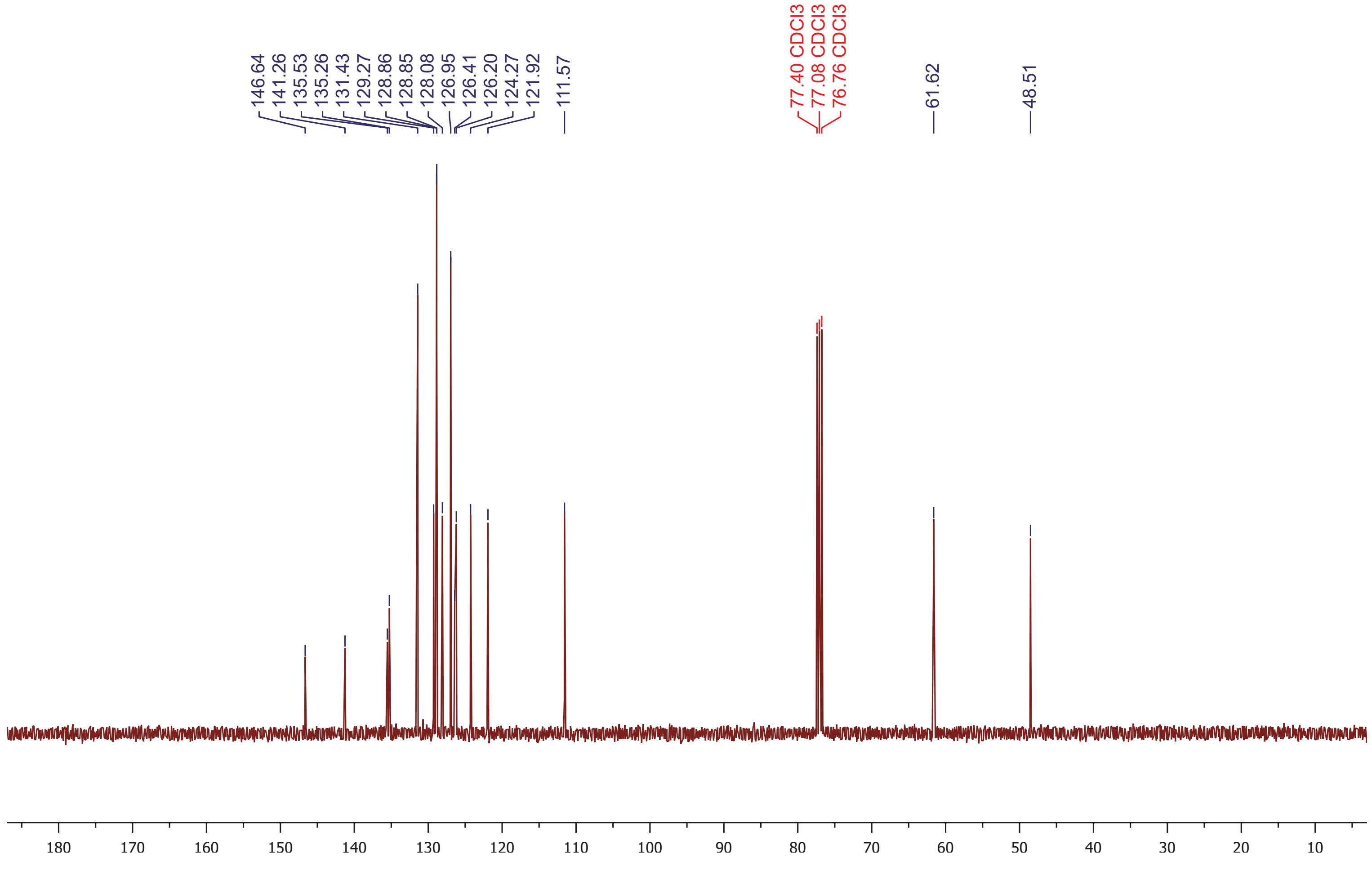
146.64 141.26 135.53 135.26 131.43 129.27 128.86 128.85 128.08 126.95 126.41 126.20 124.27 121.92
111.57
77.40 CDCl3
77.08 CDCl3
76.76 CDCl3
61.62
48.51
180 170 160 150 140 130 120 110 100 90 80 70 60 50 40 30 20 10

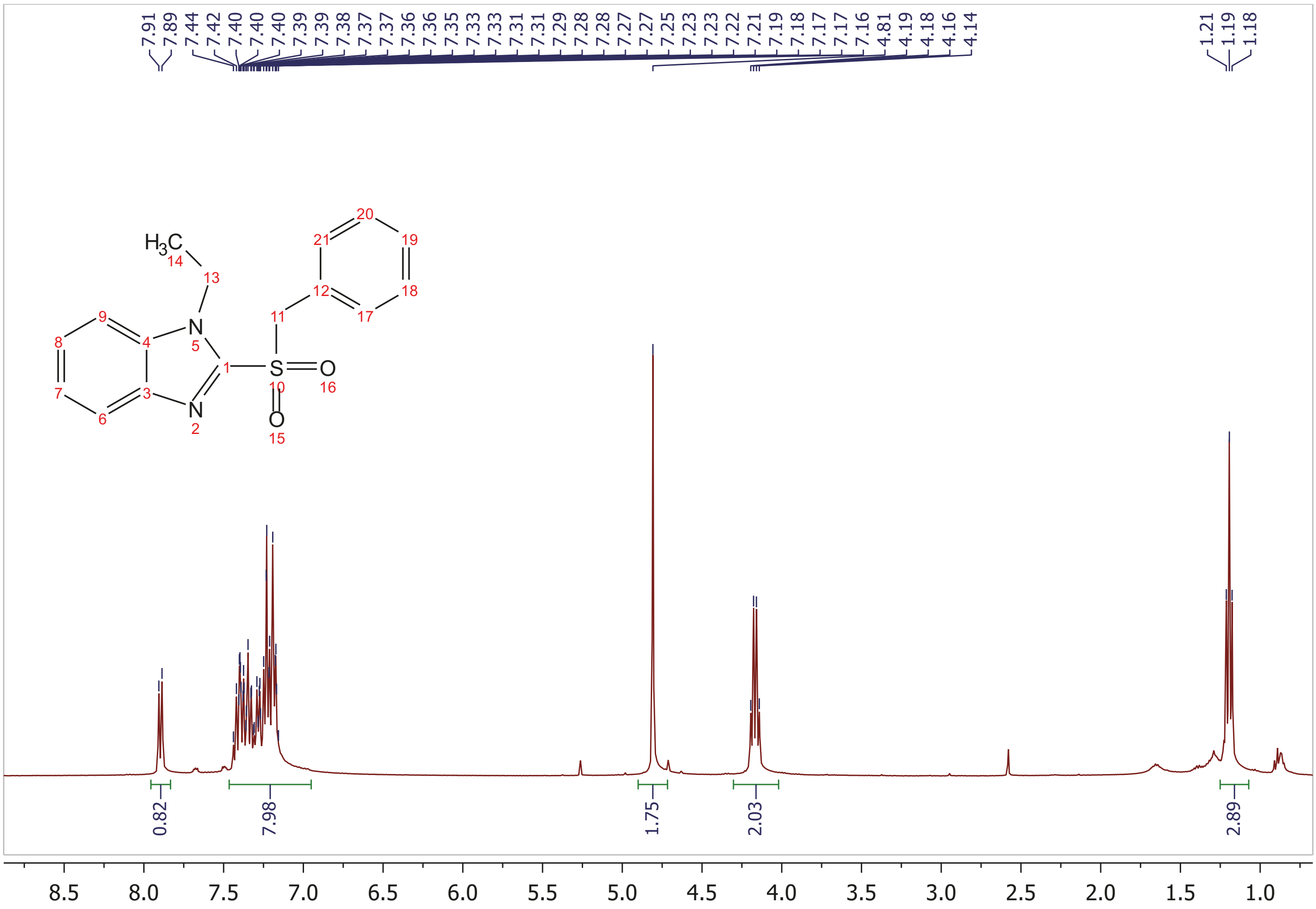
7.91
7.89
7.44
7.42
7.40
7.40
7.40
7.39
7.39
7.38
7.37
7.37
7.36
7.36
7.35
7.33
7.33
7.31
7.31
7.29
7.28
7.28
7.27
7.27
7.25
7.23
7.23
7.22
7.21
7.19
7.18
7.17
7.17
7.16
4.81
4.19
4.18
4.16
4.14
1.21
1.19
1.18
H_3C
14
13
9
8
7
6
4
3
N
5
1
N
2
11
S
10
O
16
O
15
12
21
20
19
18
17
0.82
7.98
1.75
2.03
2.89
8.5
8.0
7.5
7.0
6.5
6.0
5.5
5.0
4.5
4.0
3.5
3.0
2.5
2.0
1.5
1.0

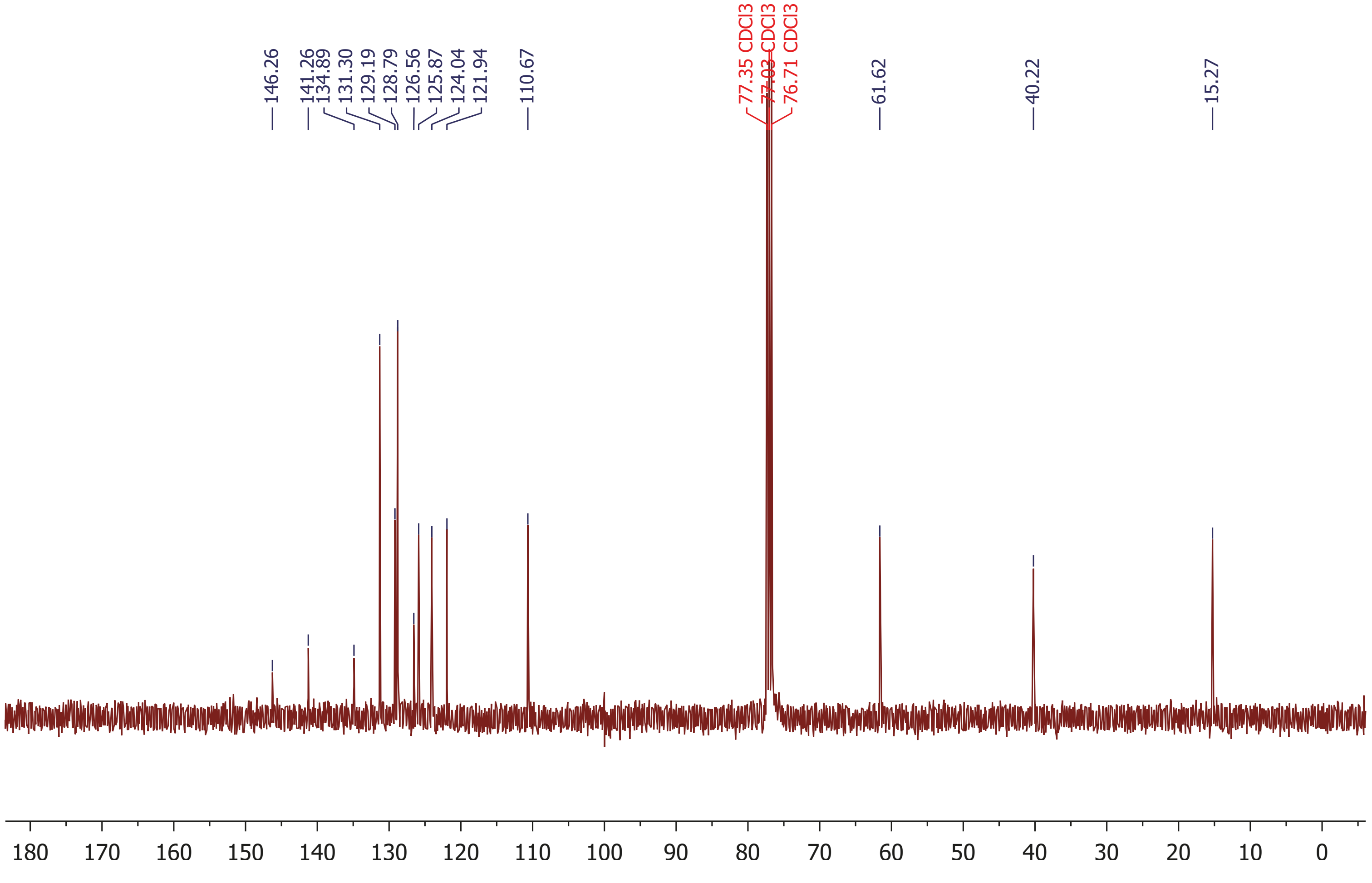
146.26
141.26
134.89
131.30
129.19
128.79
126.56
125.87
124.04
121.94
110.67
77.35 CDCl3
77.03 CDCl3
76.71 CDCl3
61.62
40.22
15.27
180
170
160
150
140
130
120
110
100
90
80
70
60
50
40
30
20
10
0

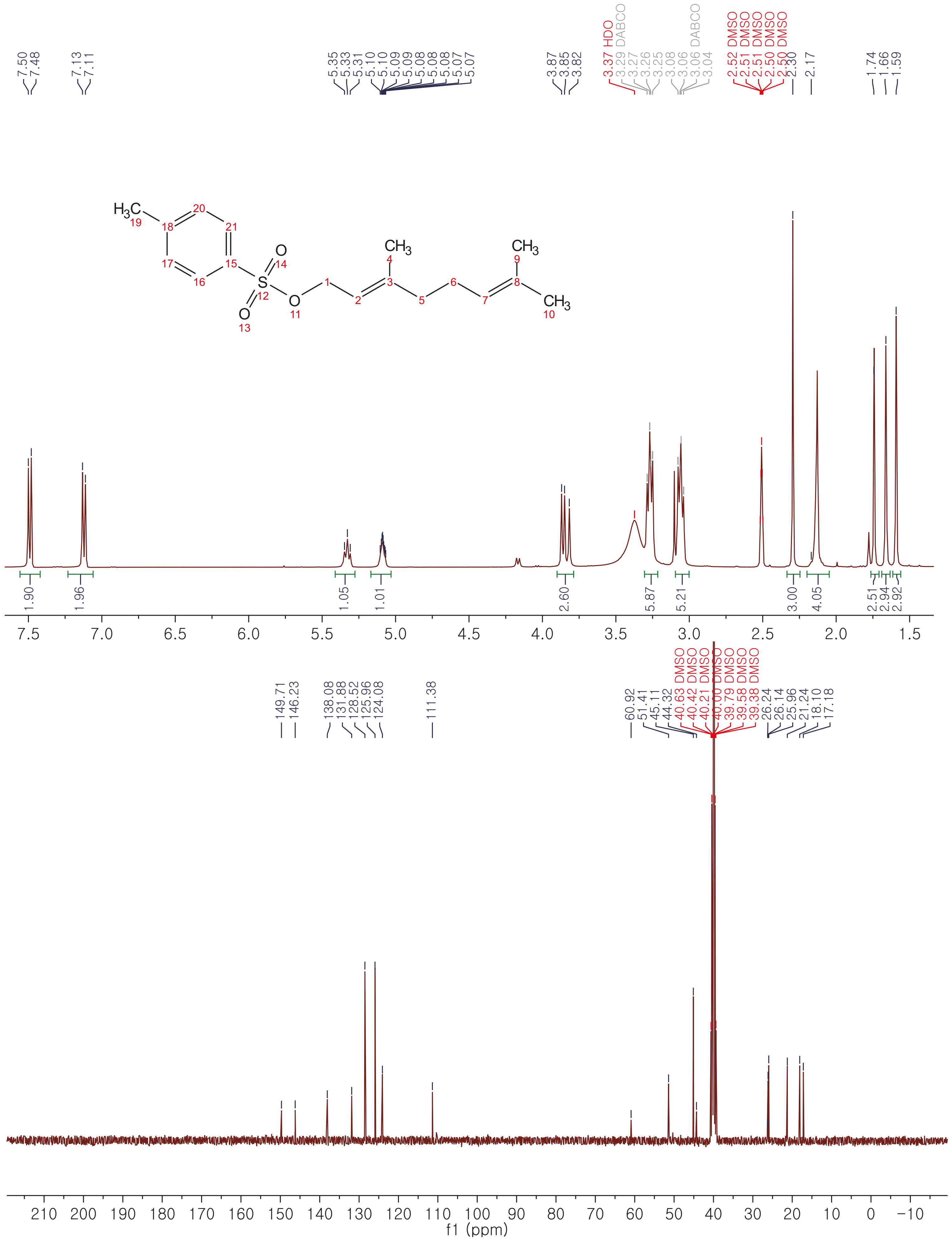

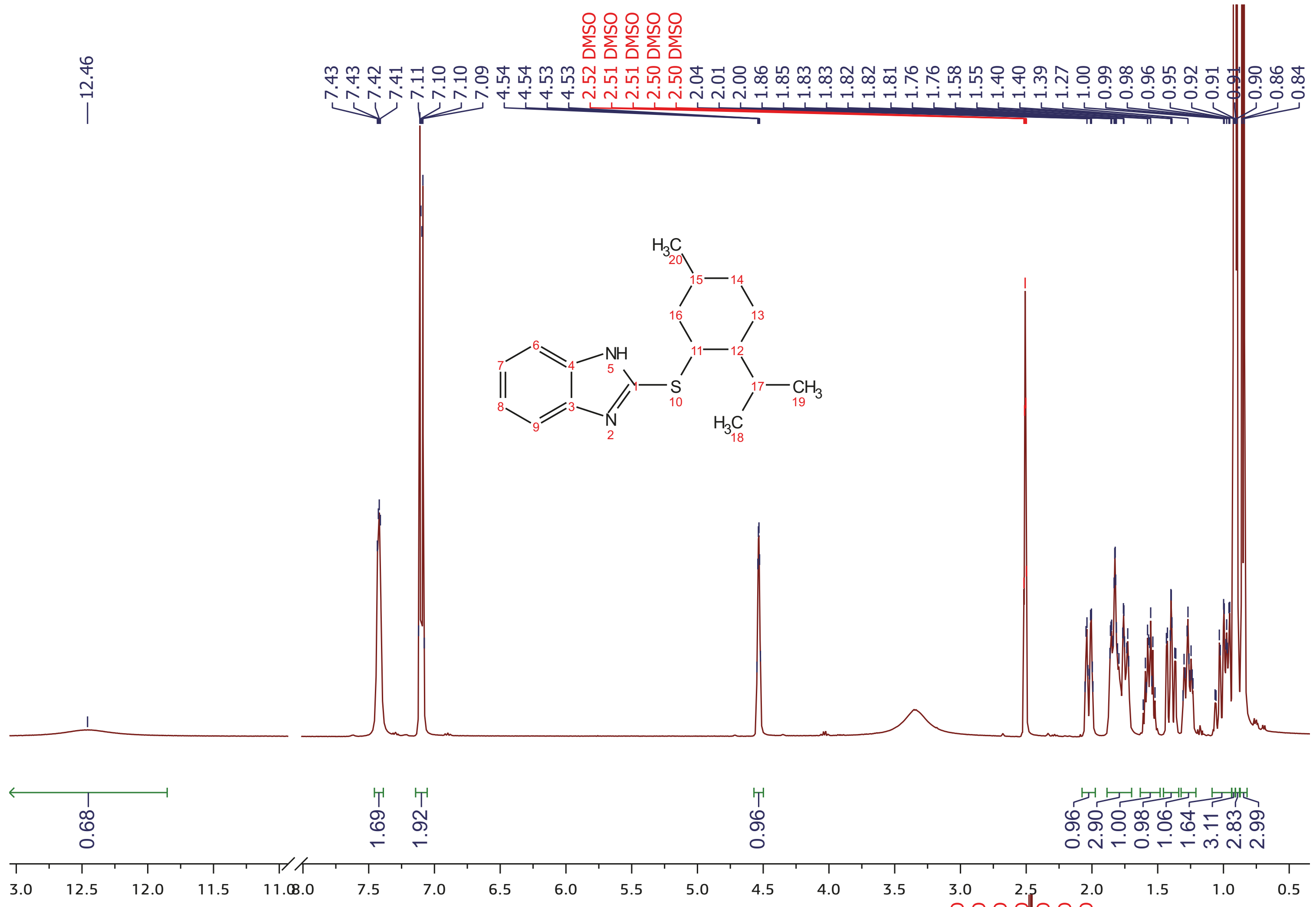

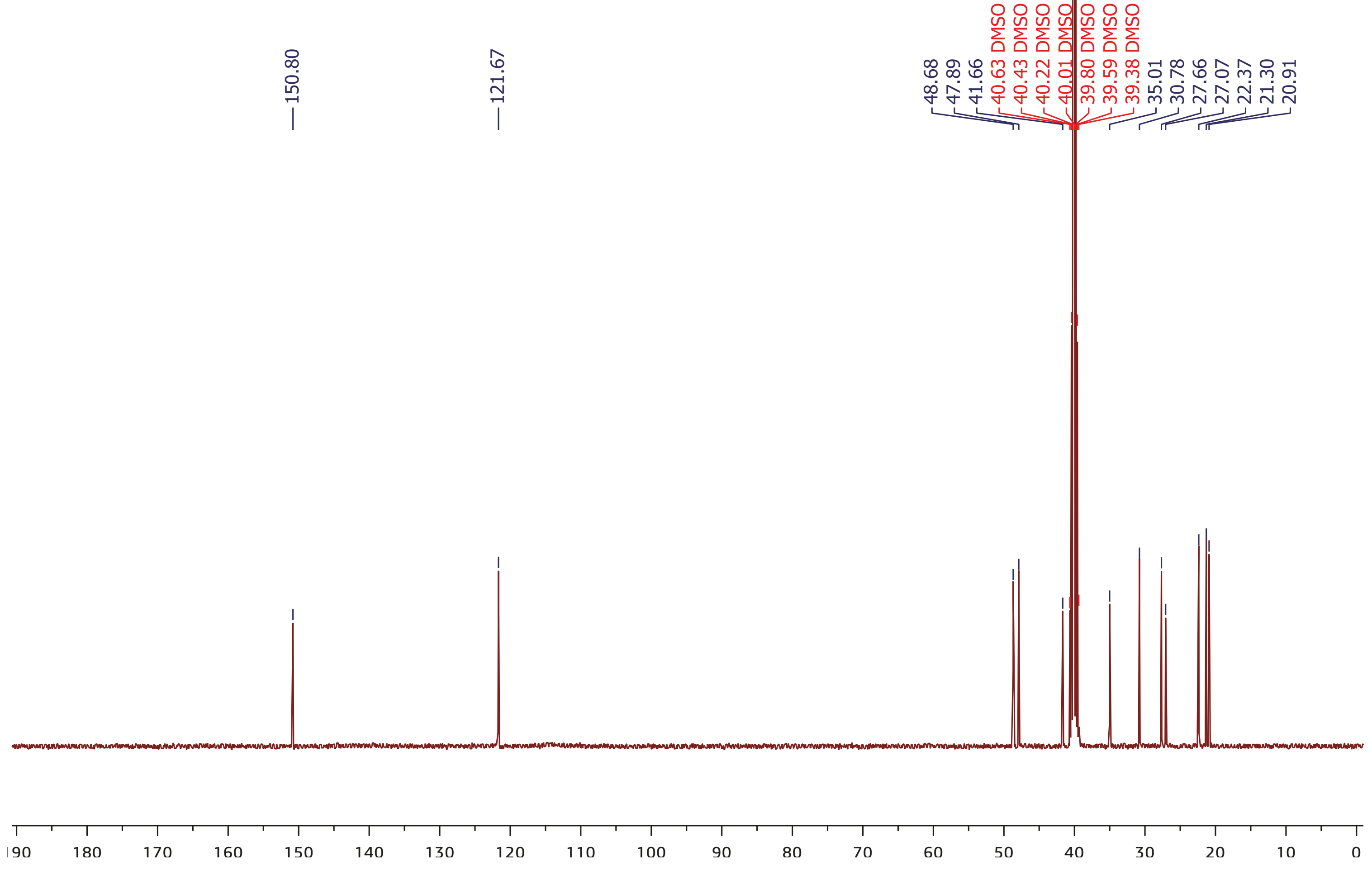

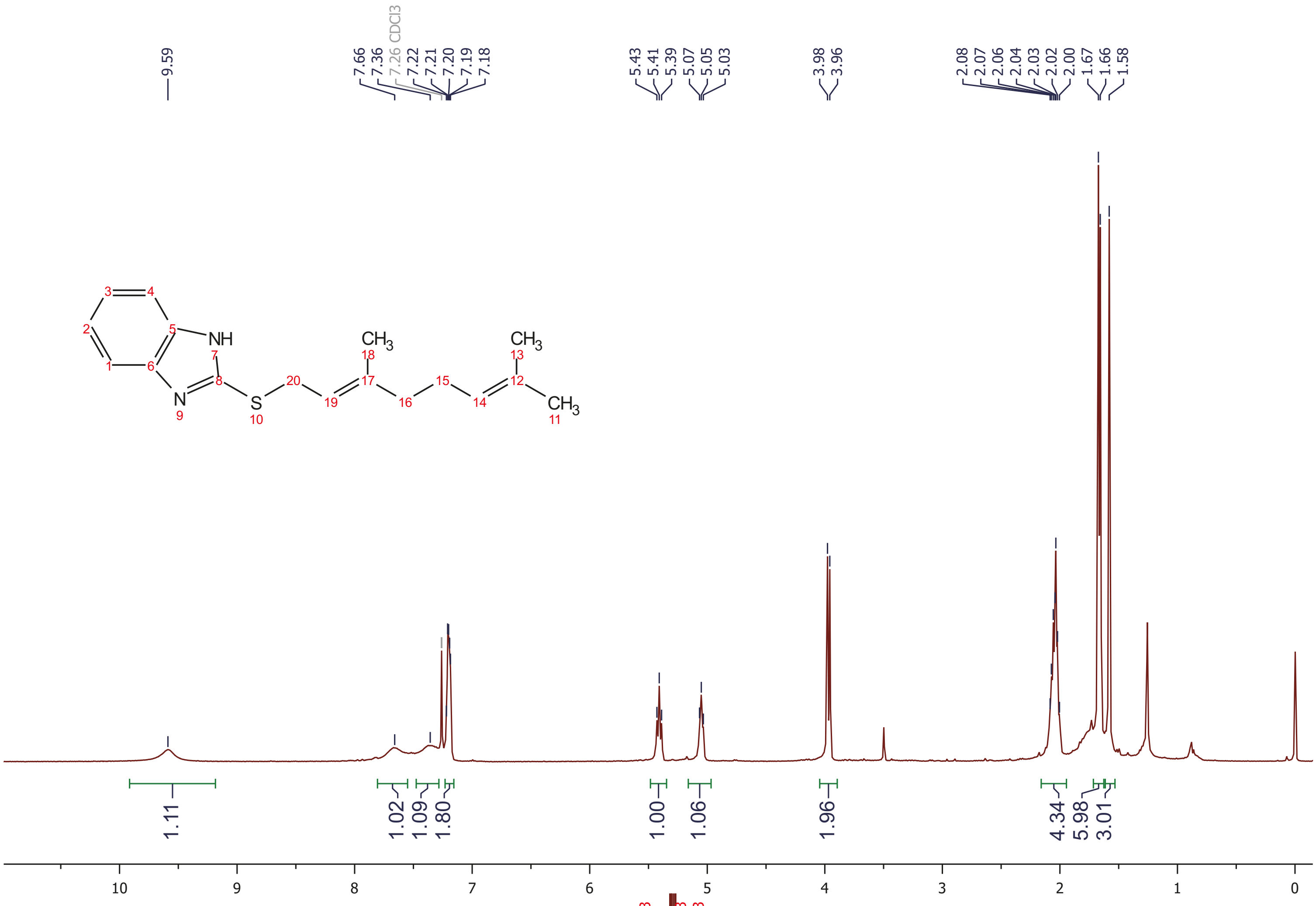

9.59
7.66
7.36
7.26 CDCl3
7.22
7.21
7.20
7.19
7.18
5.43
5.41
5.39
5.07
5.05
5.03
3.98
3.96
2.08
2.07
2.06
2.04
2.03
2.02
2.00
1.67
1.66
1.58
NH
CH3
CH3
N
S
CH3
1.11
1.02
1.09
1.80
1.00
1.06
1.96
4.34
5.98
3.01
10
9
8
7
6
5
4
3
2
1
0

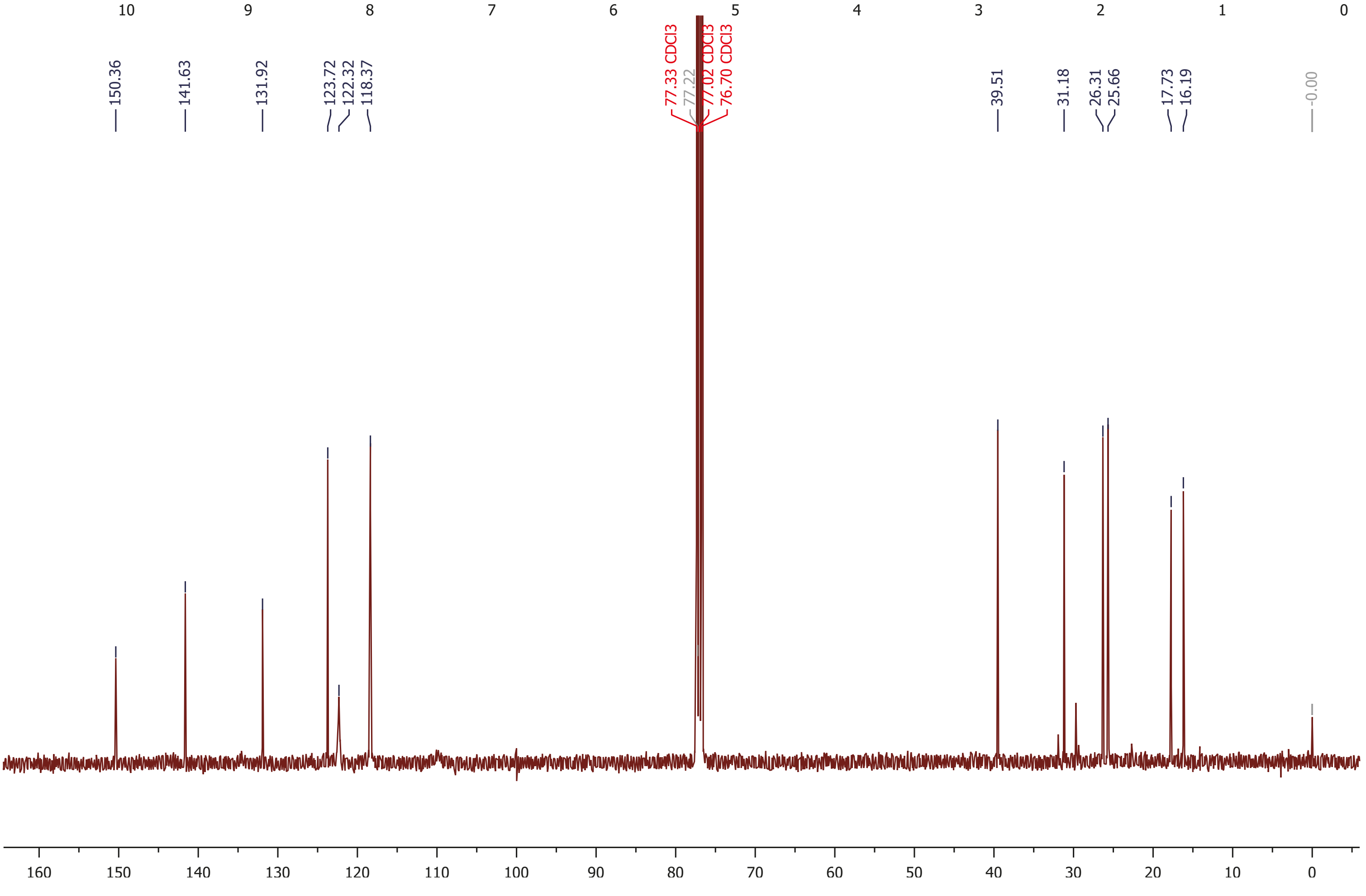

150.36
141.63
131.92
123.72
122.32
118.37
77.33 CDCl3
77.22
77.02 CDCl3
76.70 CDCl3
39.51
31.18
26.31
25.66
17.73
16.19
-0.00
160
150
140
130
120
110
100
90
80
70
60
50
40
30
20
10
0

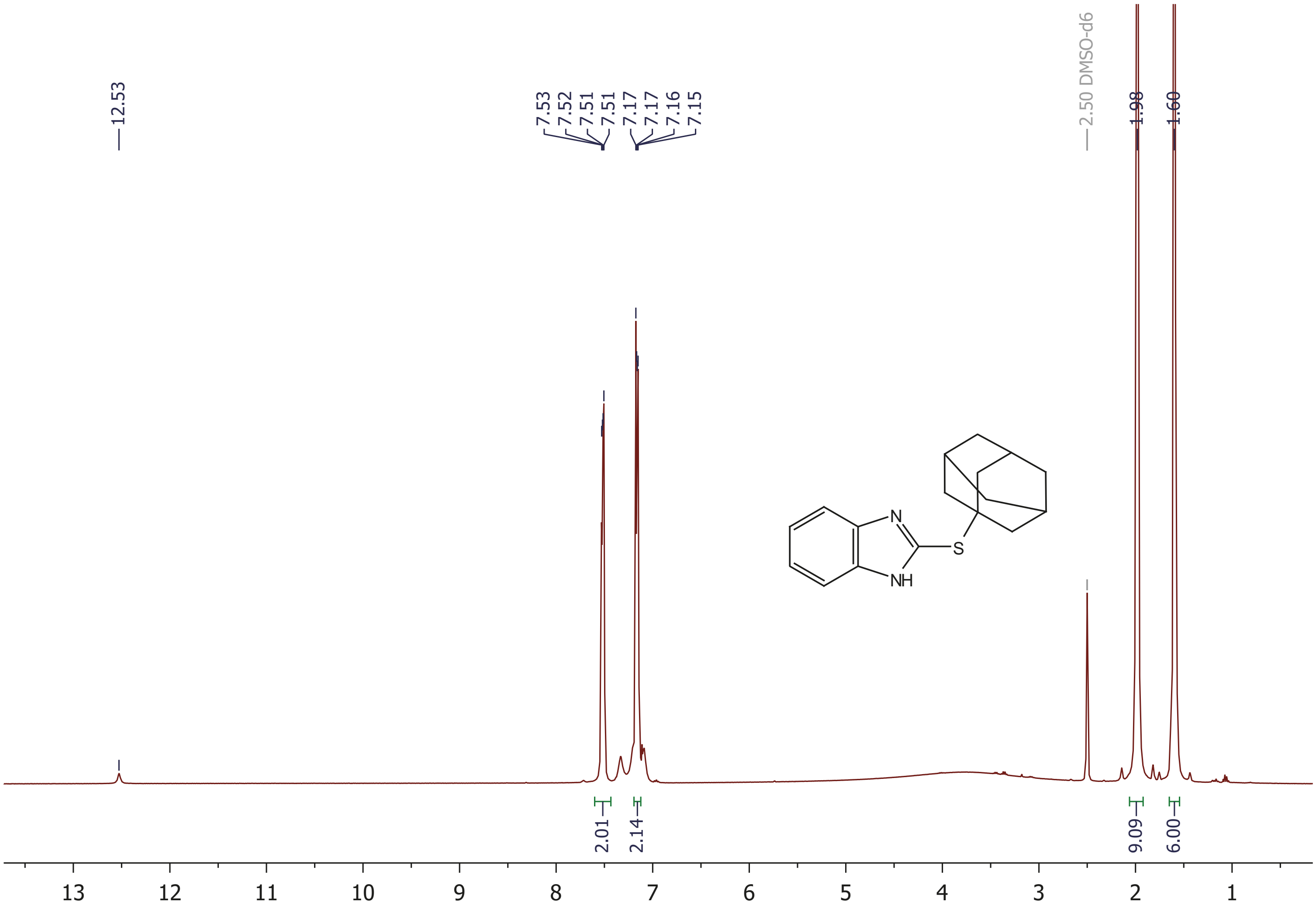
12.53
7.53
7.52
7.51
7.51
7.17
7.17
7.16
7.15
2.50 DMSO-d6
1.98
1.60
N
S
NH
2.01
2.14
9.09
6.00
13
12
11
10
9
8
7
6
5
4
3
2
1

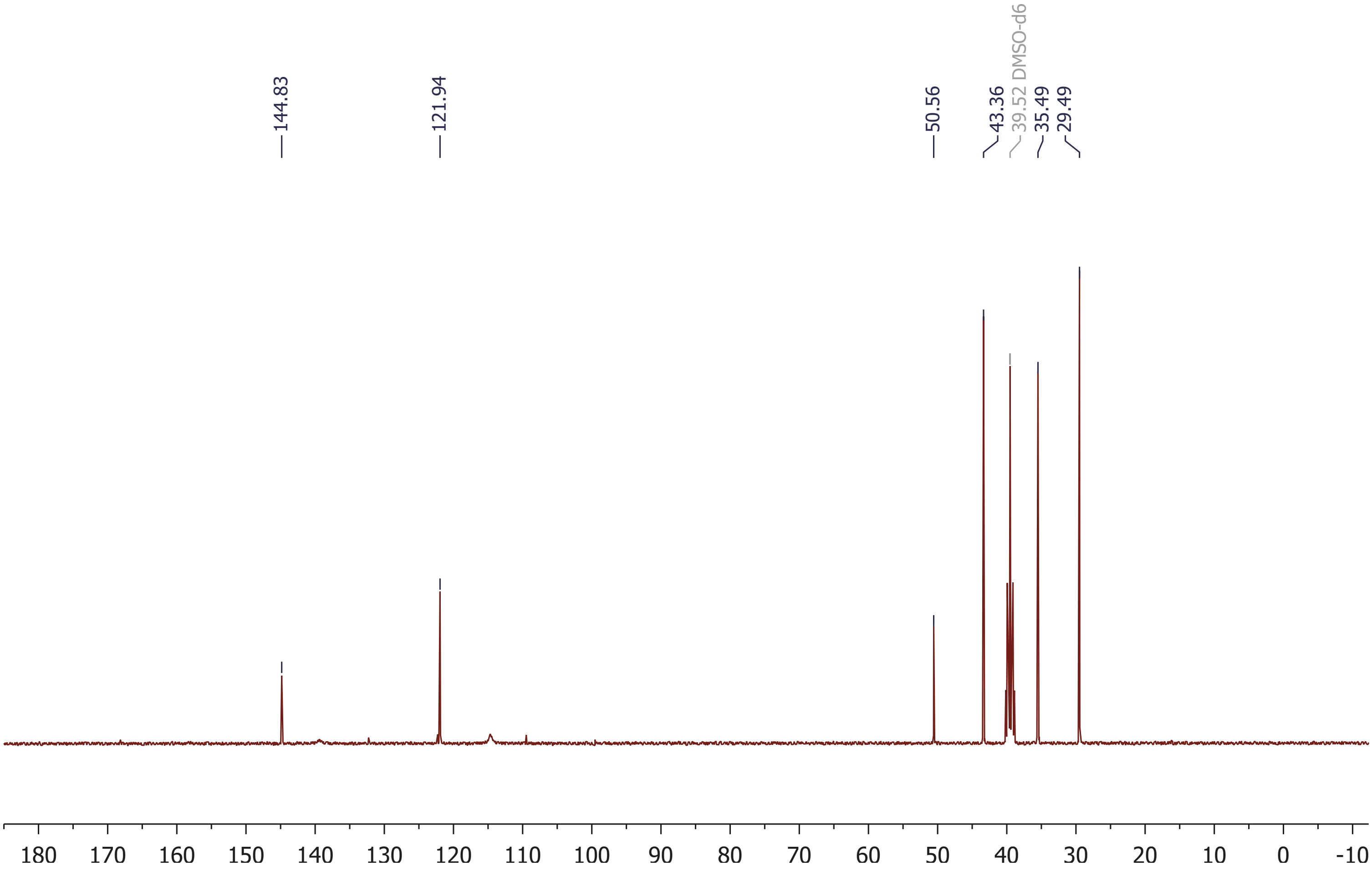
144.83
121.94
50.56
43.36
39.52 DMSO-d6
35.49
29.49
180
170
160
150
140
130
120
110
100
90
80
70
60
50
40
30
20
10
0
-10

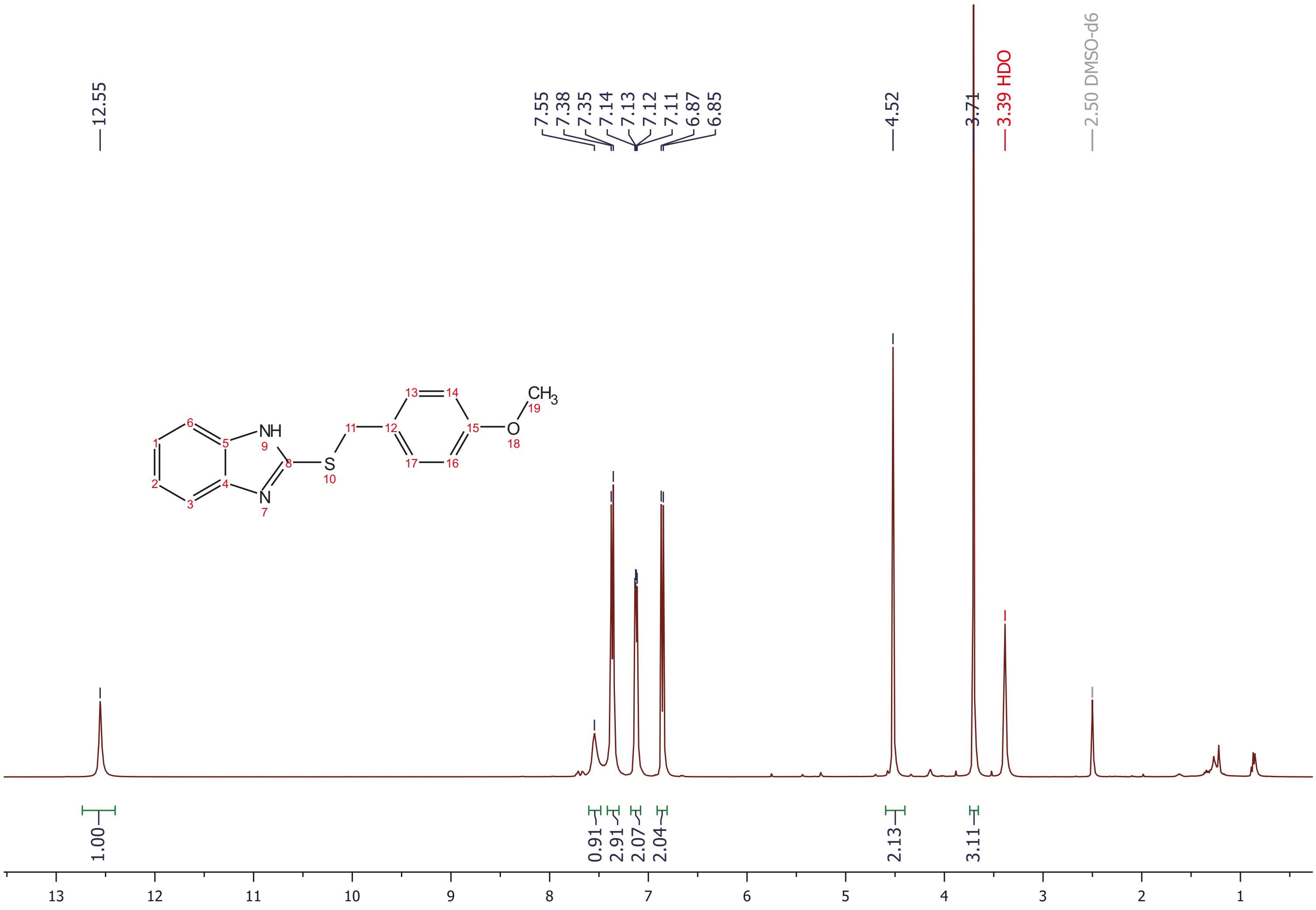
12.55
7.55
7.38
7.35
7.14
7.13
7.12
7.11
6.87
6.85
4.52
3.71
3.39 HDO
2.50 DMSO-d6
NH
S
N
O
CH3
1.00
0.91
2.91
2.07
2.04
2.13
3.11
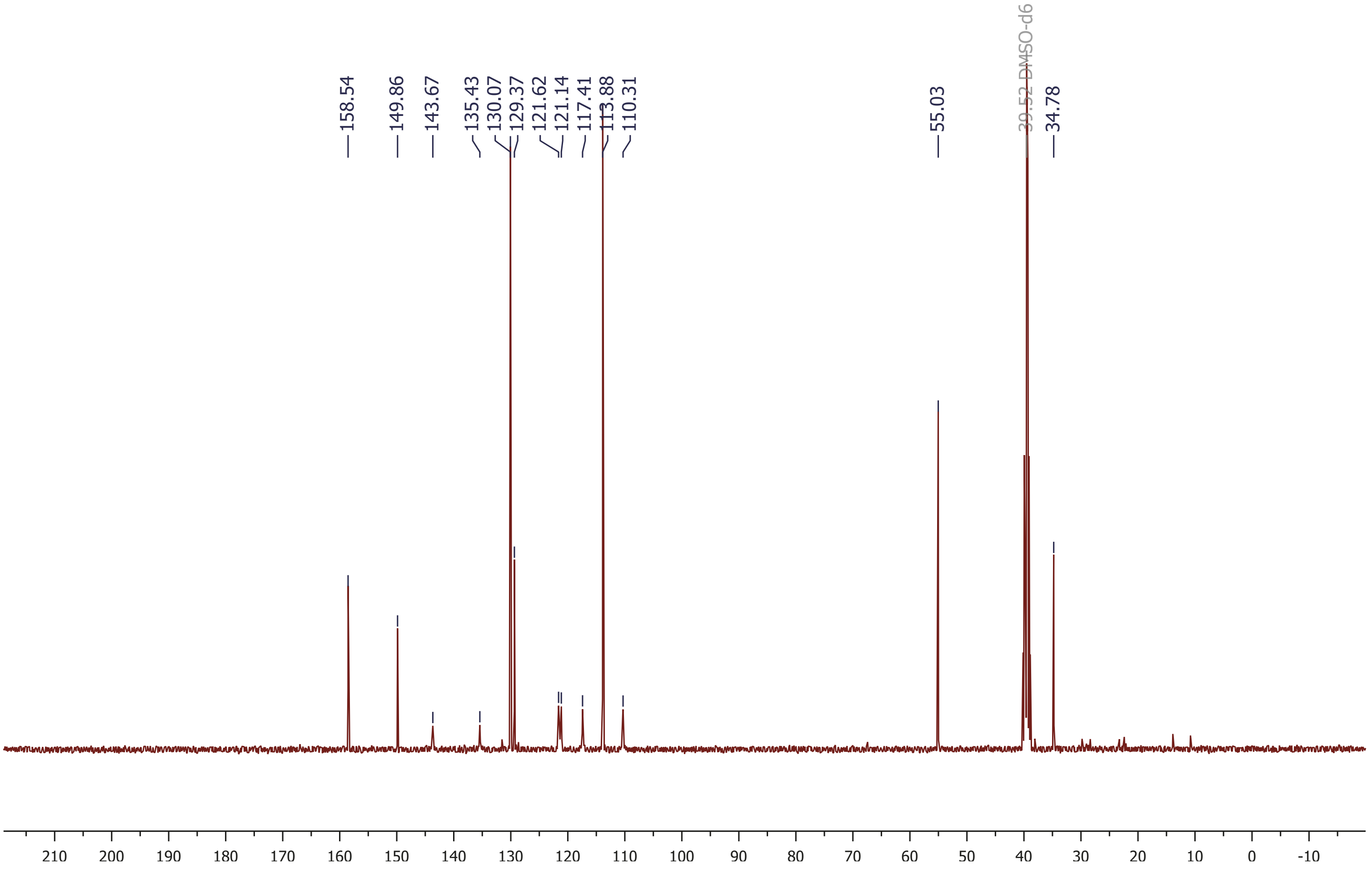
158.54
149.86
143.67
135.43
130.07
129.37
121.62
121.14
117.41
113.88
110.31
55.03
39.52 DMSO-d6
34.78

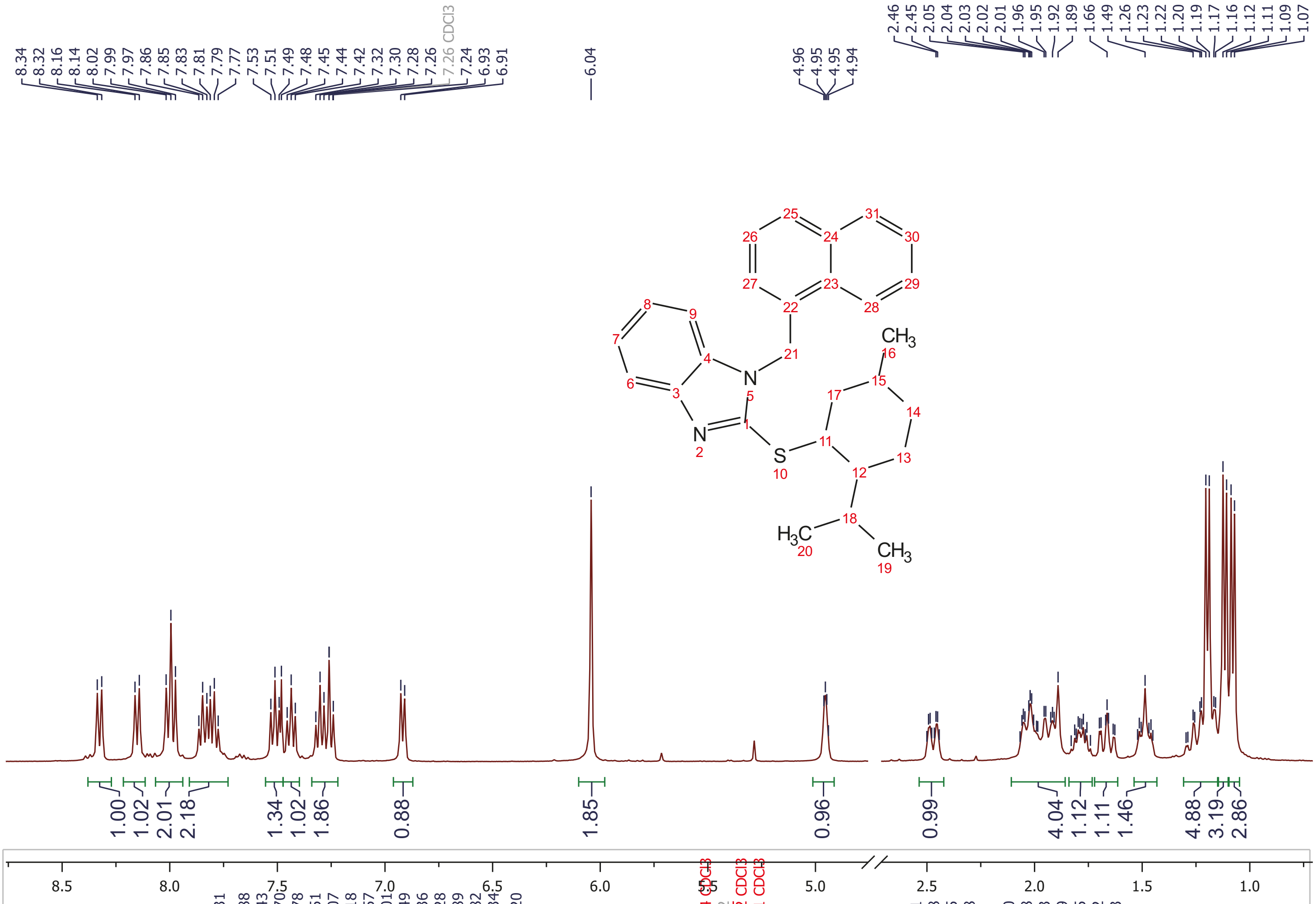
8.34
8.32
8.16
8.14
8.02
7.99
7.97
7.86
7.85
7.83
7.81
7.79
7.77
7.53
7.51
7.49
7.48
7.45
7.44
7.42
7.32
7.30
7.28
7.26
7.26 CDCl3
7.24
6.93
6.91
6.04
4.96
4.95
4.95
4.94
2.46
2.45
2.05
2.04
2.03
2.02
2.01
1.96
1.95
1.92
1.89
1.66
1.49
1.26
1.23
1.22
1.20
1.19
1.17
1.16
1.12
1.11
1.09
1.07
N
N
S
CH3
H3C
CH3
1.00
1.02
2.01
2.18
1.34
1.02
1.86
0.88
1.85
0.96
0.99
4.04
1.12
1.11
1.46
4.88
3.19
2.86
8.5
8.0
7.5
7.0
6.5
6.0
5.5
5.0
2.5
2.0
1.5
1.0

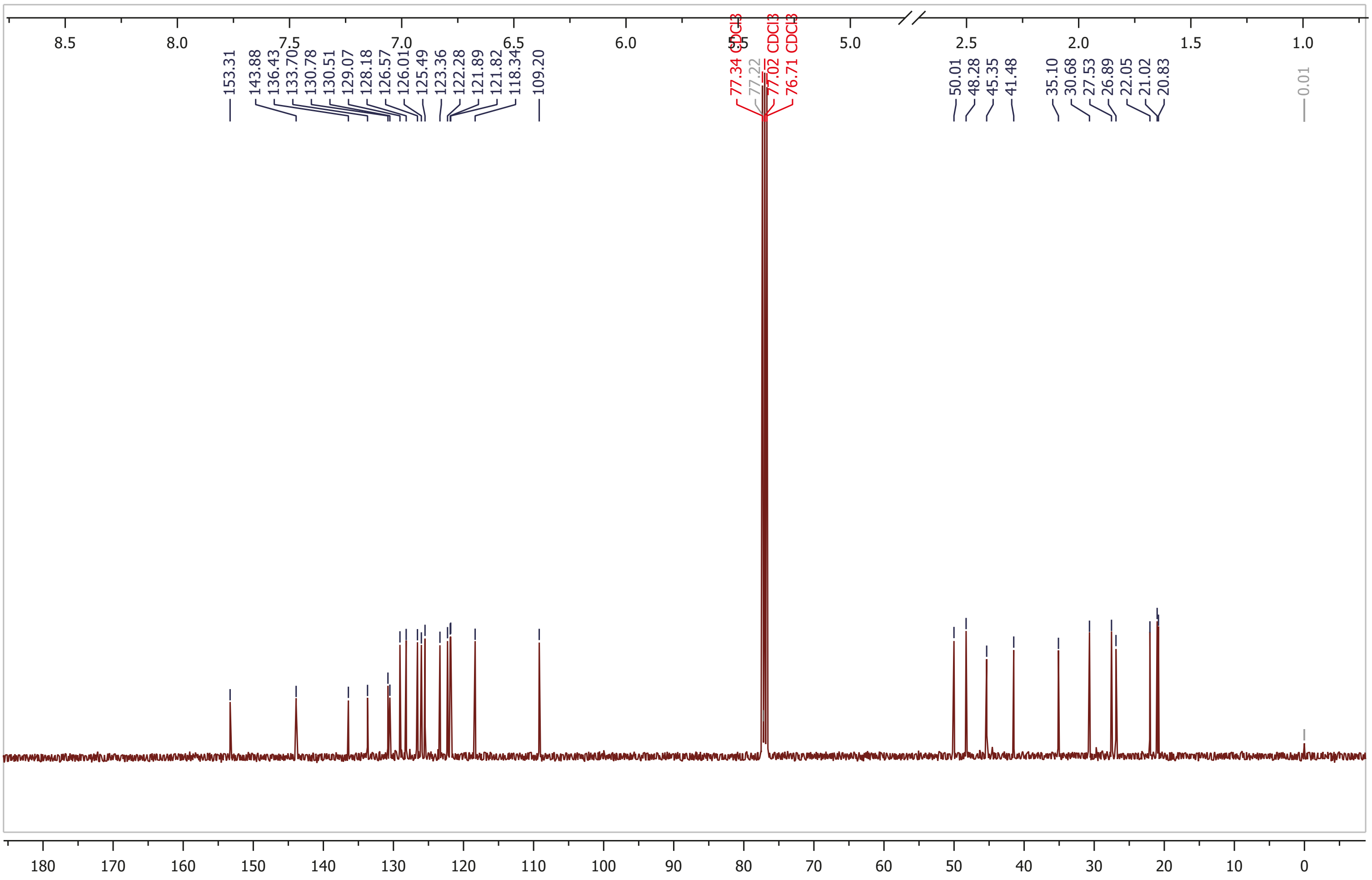
153.31
143.88
136.43
133.70
130.78
130.51
129.07
128.18
126.57
126.01
125.49
123.36
122.28
121.89
121.82
118.34
109.20
77.34 CDCl3
77.22
77.02 CDCl3
76.71 CDCl3
50.01
48.28
45.35
41.48
35.10
30.68
27.53
26.89
22.05
21.02
20.83
0.01
180
170
160
150
140
130
120
110
100
90
80
70
60
50
40
30
20
10
0

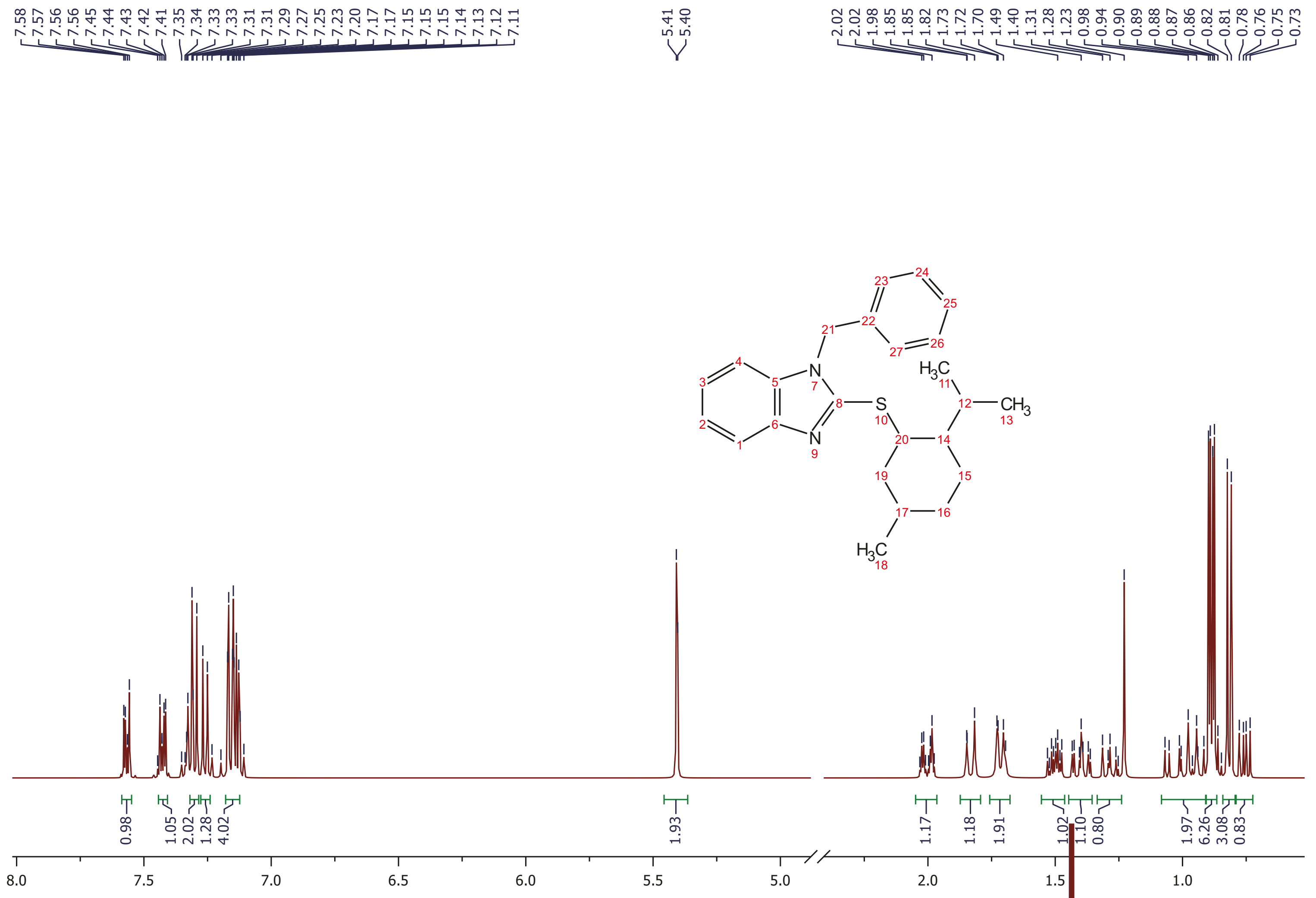

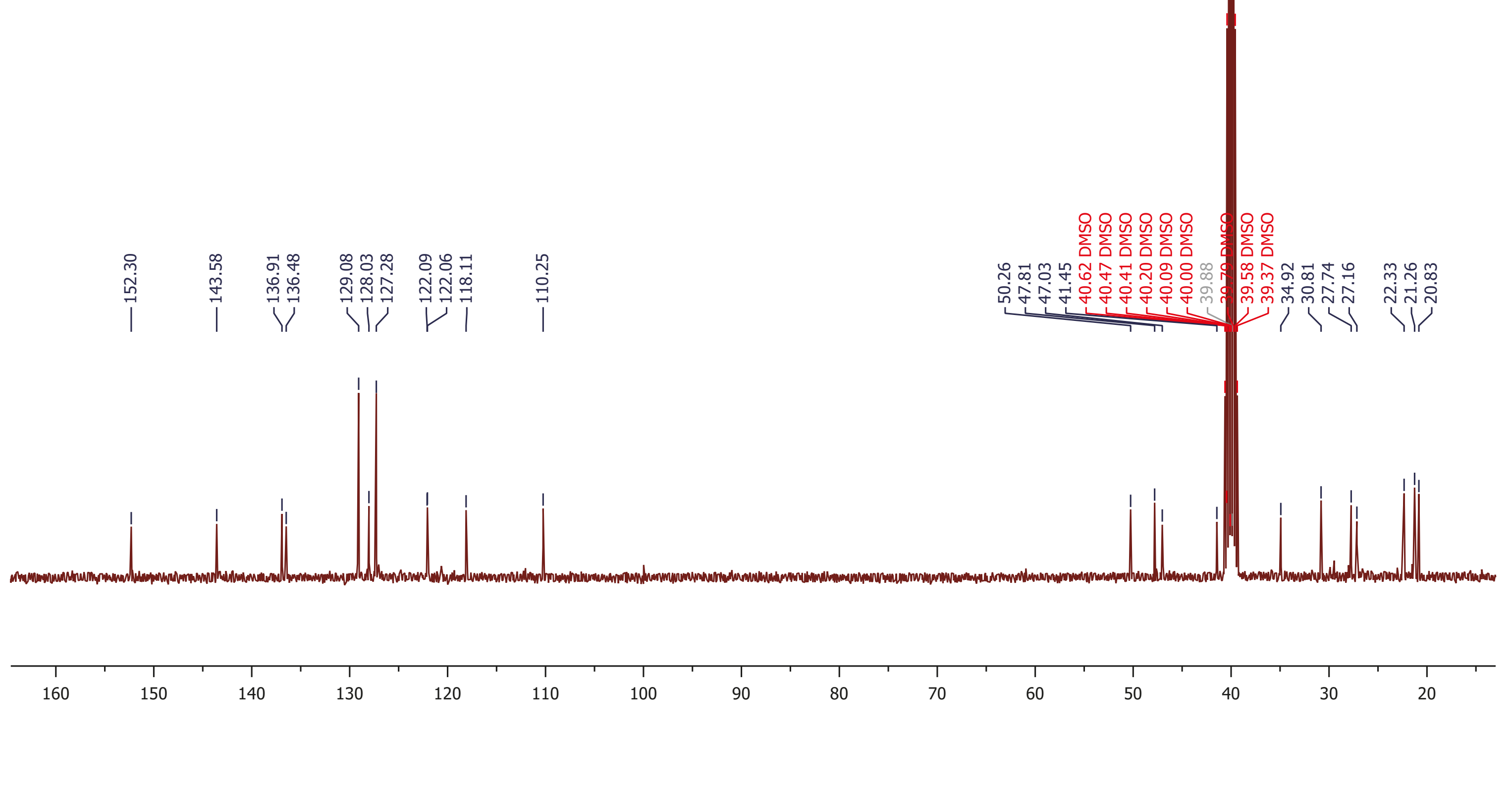

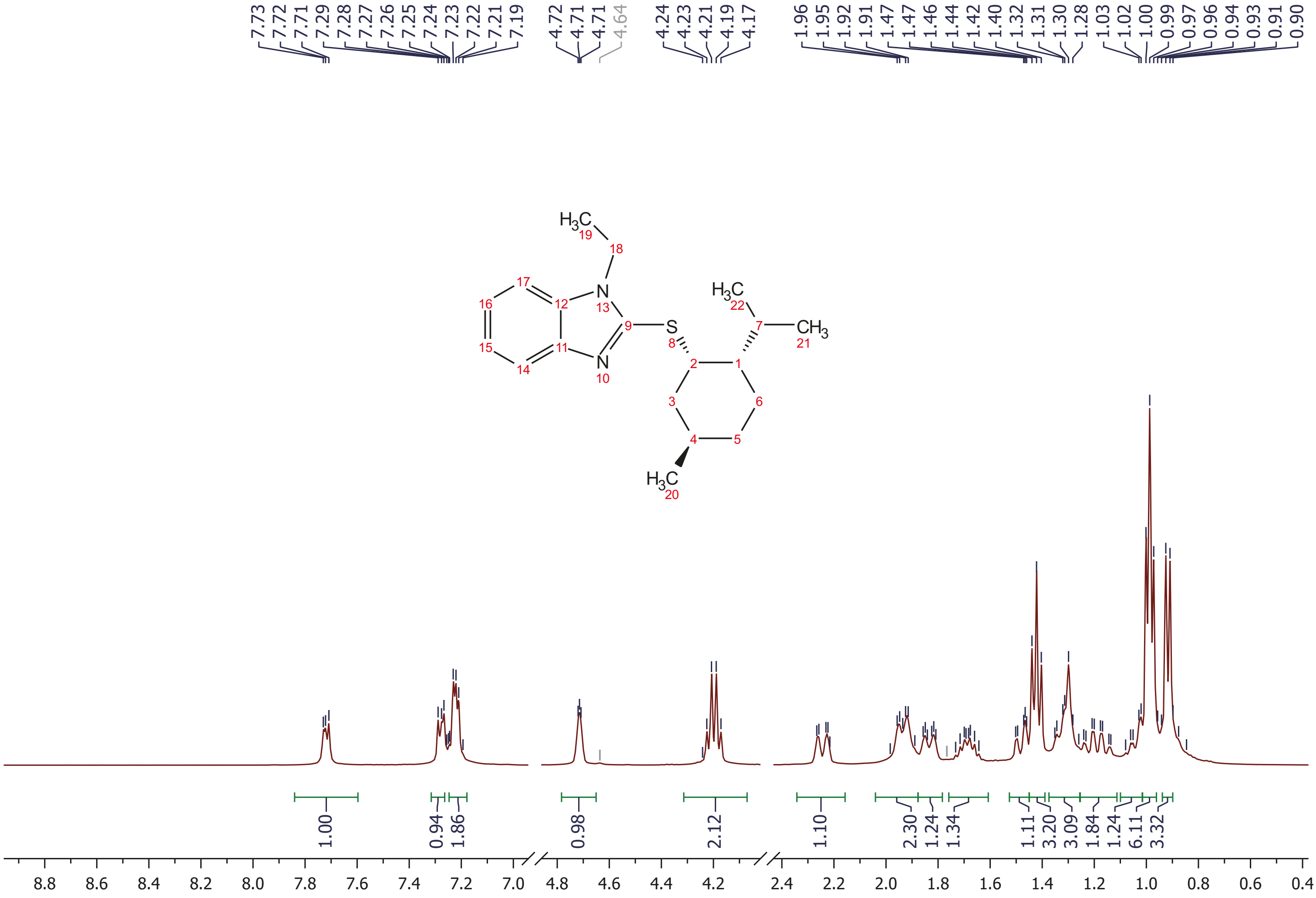

7.73 7.72 7.71 7.29 7.28 7.27 7.26 7.25 7.24 7.23 7.22 7.21 7.19
4.72 4.71 4.71 4.64
4.24 4.23 4.21 4.19 4.17
1.96 1.95 1.92 1.91 1.47 1.47 1.46 1.44 1.42 1.40 1.32 1.31 1.30 1.28 1.03 1.02 1.00 0.99 0.97 0.96 0.94 0.93 0.91 0.90
H_3C 19 18 17 16 15 14 12 11 N 13 9 S 8 N 10 H_3C 22 7 CH_3 21 2 1 3 6 4 5 H_3C 20
1.00 0.94 1.86 0.98 2.12 1.10 2.30 1.24 1.34 1.11 3.20 3.09 1.84 1.24 6.11 3.32
8.8 8.6 8.4 8.2 8.0 7.8 7.6 7.4 7.2 7.0 4.8 4.6 4.4 4.2 2.4 2.2 2.0 1.8 1.6 1.4 1.2 1.0 0.8 0.6 0.4

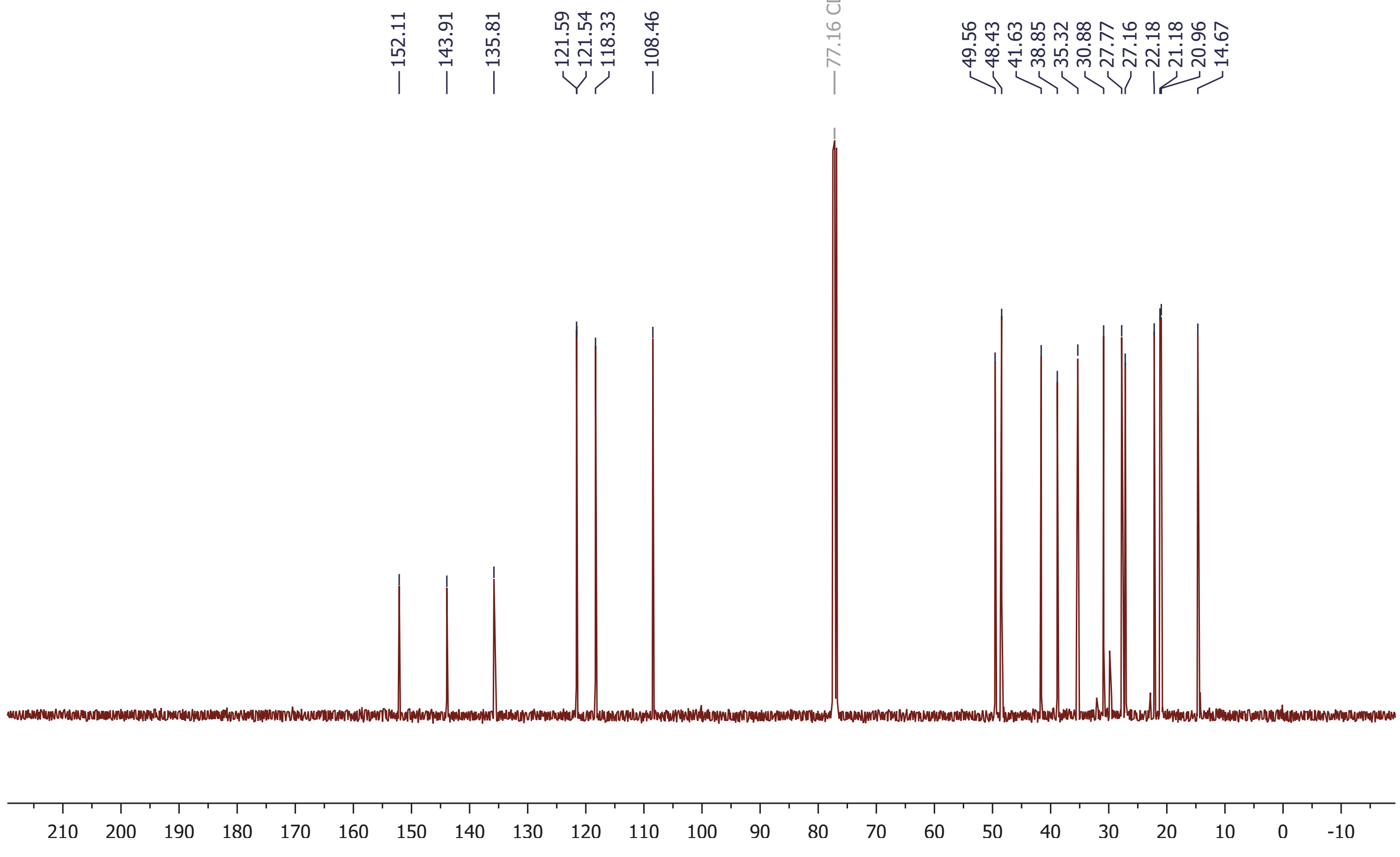

152.11 143.91 135.81 121.59 121.54 118.33 108.46
77.16 CDCl3
49.56 48.43 41.63 38.85 35.32 30.88 27.77 27.16 22.18 21.18 20.96 14.67
210 200 190 180 170 160 150 140 130 120 110 100 90 80 70 60 50 40 30 20 10 0 -10

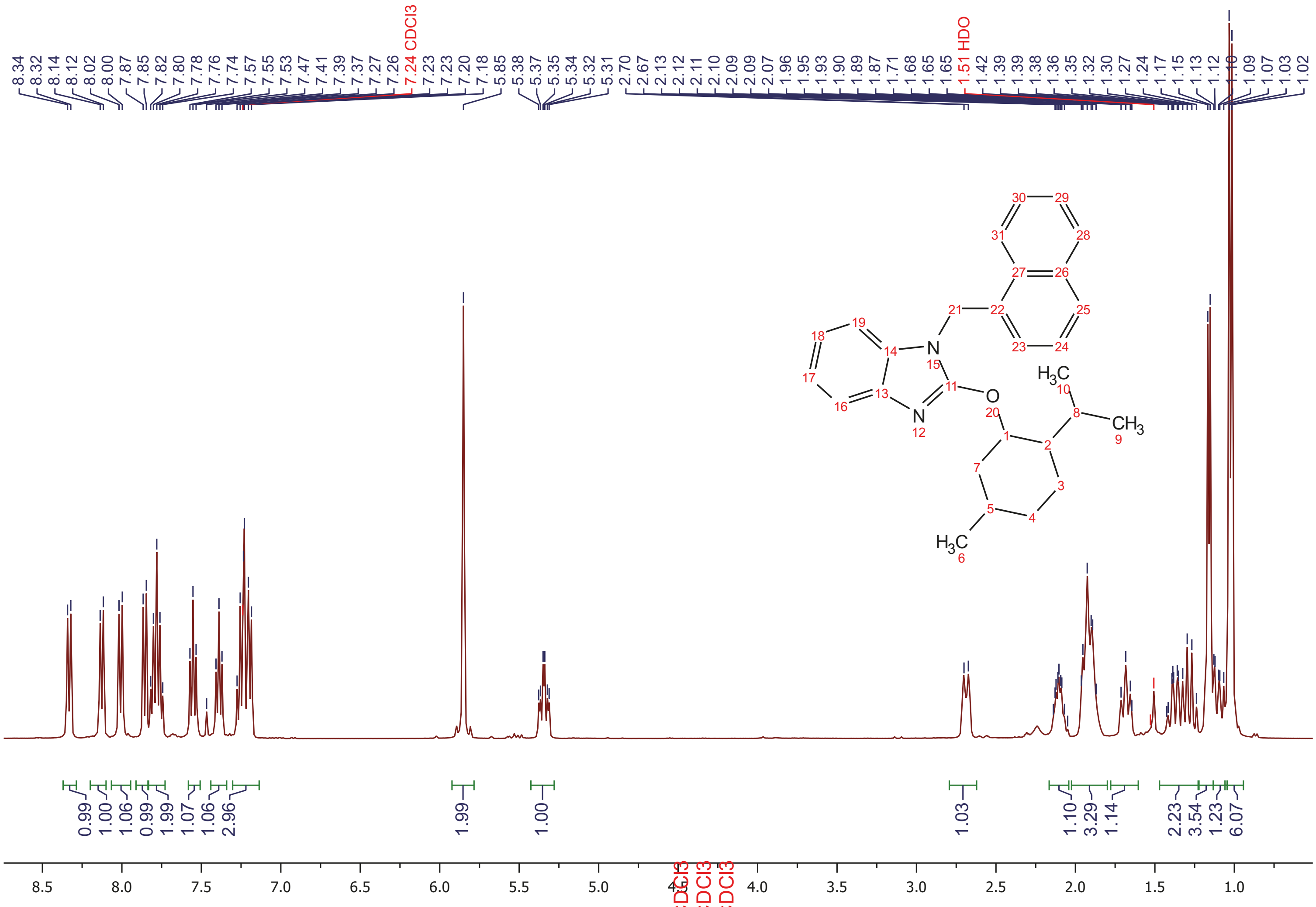
8.34 8.32 8.14 8.12 8.02 8.00 7.87 7.85 7.82 7.80 7.78 7.76 7.74 7.57 7.55 7.53 7.47 7.41 7.39 7.37 7.27 7.26 7.24 CDCl3 7.23 7.23 7.20 7.18 5.85 5.38 5.37 5.35 5.34 5.32 5.31 2.70 2.67 2.13 2.12 2.11 2.10 2.09 2.07 1.96 1.95 1.93 1.90 1.89 1.87 1.71 1.68 1.65 1.51 HDO 1.42 1.39 1.39 1.38 1.36 1.35 1.32 1.30 1.27 1.24 1.17 1.15 1.13 1.12 1.10 1.09 1.07 1.03 1.02
0.99 1.00 1.06 0.99 1.99 1.07 1.06 2.96 1.99 1.00 1.03 1.10 3.29 1.14 2.23 3.54 1.23 6.07
8.5 8.0 7.5 7.0 6.5 6.0 5.5 5.0 4.5 4.0 3.5 3.0 2.5 2.0 1.5 1.0
H3C CH3 H3C O N N
1 2 3 4 5 6 7 8 9 10 11 12 13 14 15 16 17 18 19 20 21 22 23 24 25 26 27 28 29 30 31
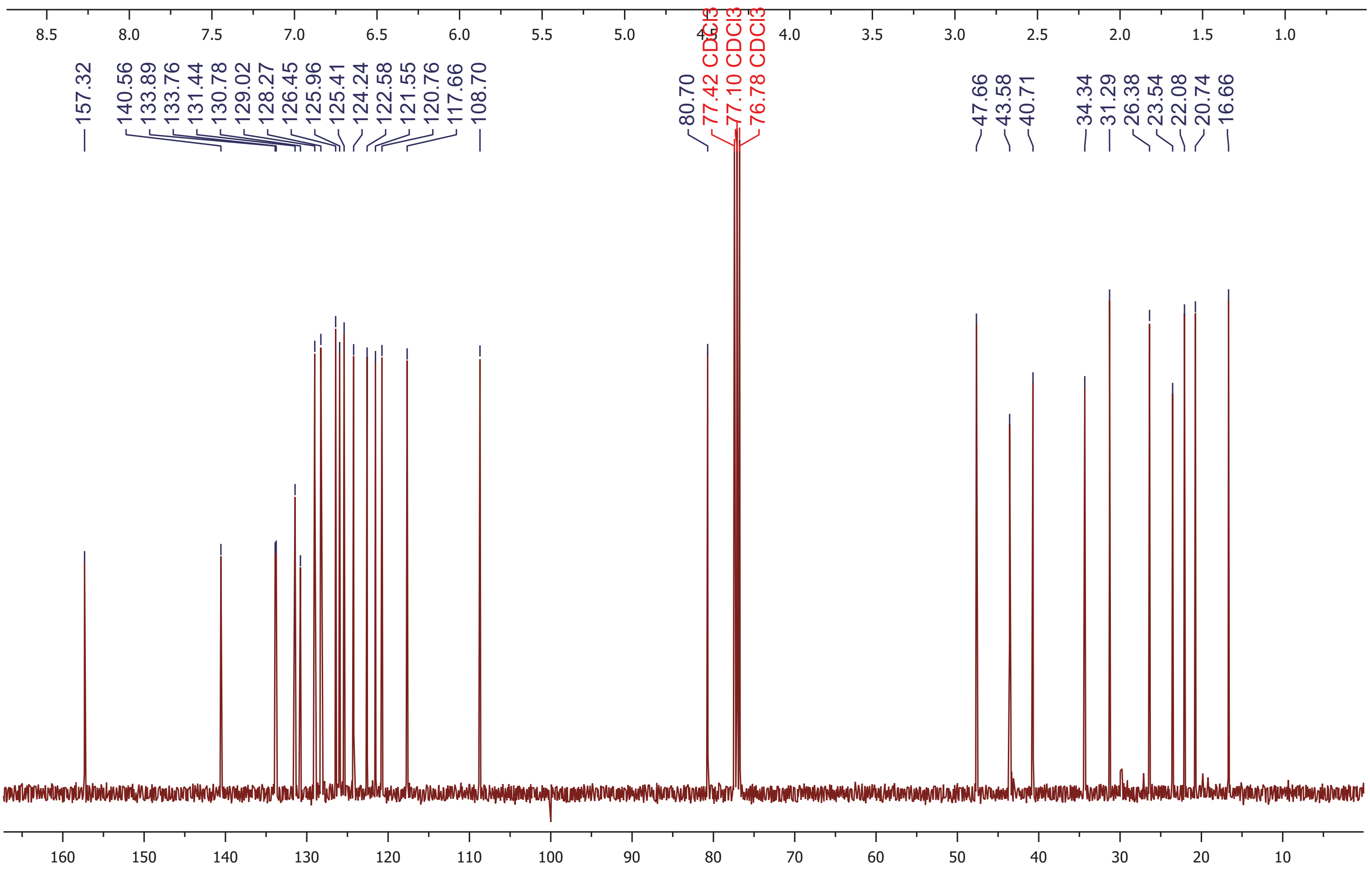
157.32 140.56 133.89 133.76 131.44 130.78 129.02 128.27 126.45 125.96 125.41 124.24 122.58 121.55 120.76 117.66 108.70 80.70 77.42 CDCl3 77.10 CDCl3 76.78 CDCl3 47.66 43.58 40.71 34.34 31.29 26.38 23.54 22.08 20.74 16.66
160 150 140 130 120 110 100 90 80 70 60 50 40 30 20 10

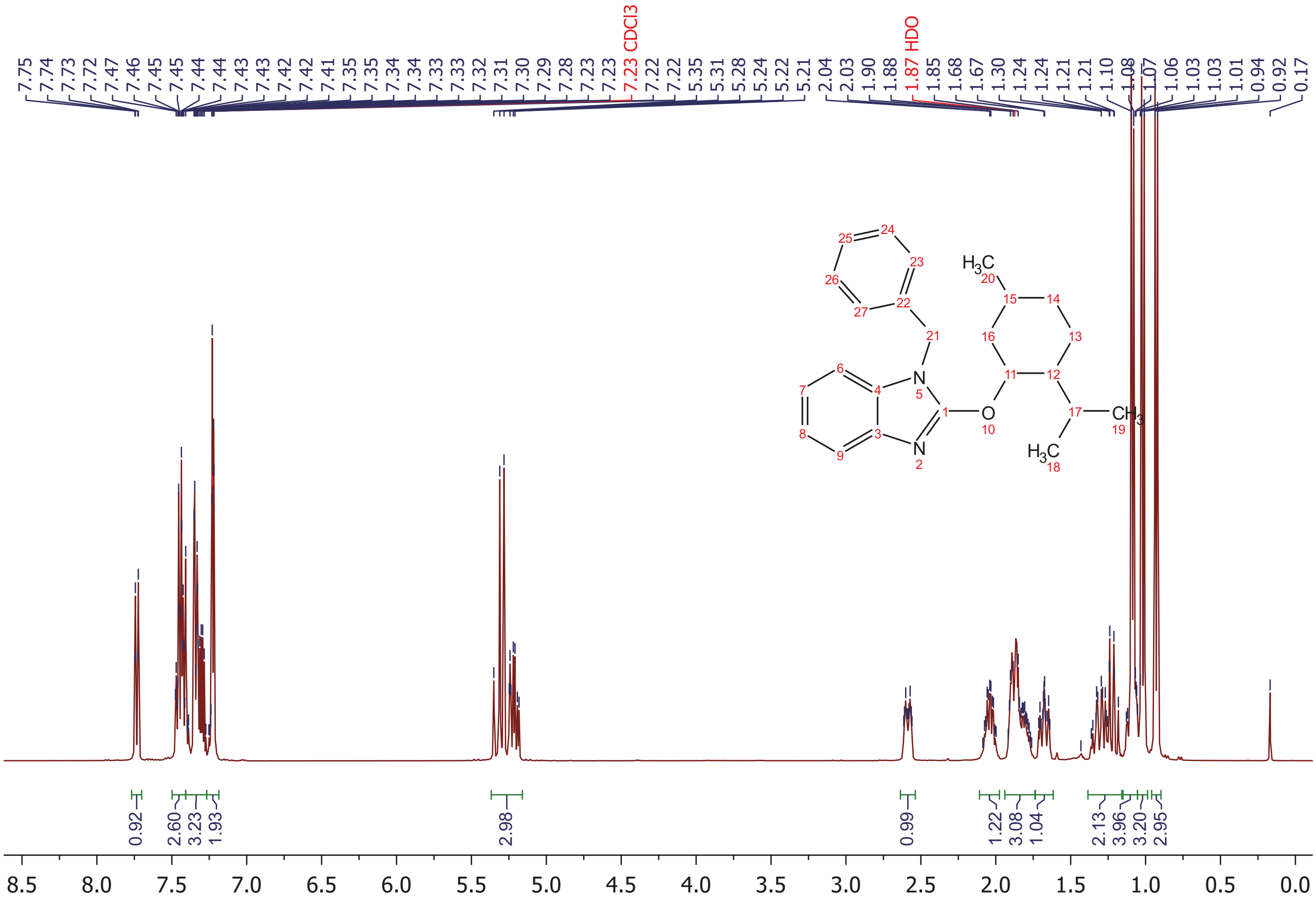
7.75 7.74 7.73 7.72 7.47 7.46 7.45 7.45 7.44 7.44 7.43 7.43 7.42 7.42 7.41 7.35 7.35 7.34 7.34 7.33 7.33 7.32 7.31 7.30 7.29 7.28 7.23 7.23
7.23 CDCl3
7.22 7.22 7.22 5.35 5.31 5.28 5.24 5.22 5.21 2.04 2.03 1.90 1.88
1.87 HDO
1.85 1.68 1.67 1.30 1.24 1.24 1.21 1.21 1.10 1.08 1.07 1.06 1.03 1.03 1.01 0.94 0.92 0.17
0.92 2.60 3.23 1.93 2.98 0.99 1.22 3.08 1.04 2.13 3.96 3.20 2.95
8.5 8.0 7.5 7.0 6.5 6.0 5.5 5.0 4.5 4.0 3.5 3.0 2.5 2.0 1.5 1.0 0.5 0.0

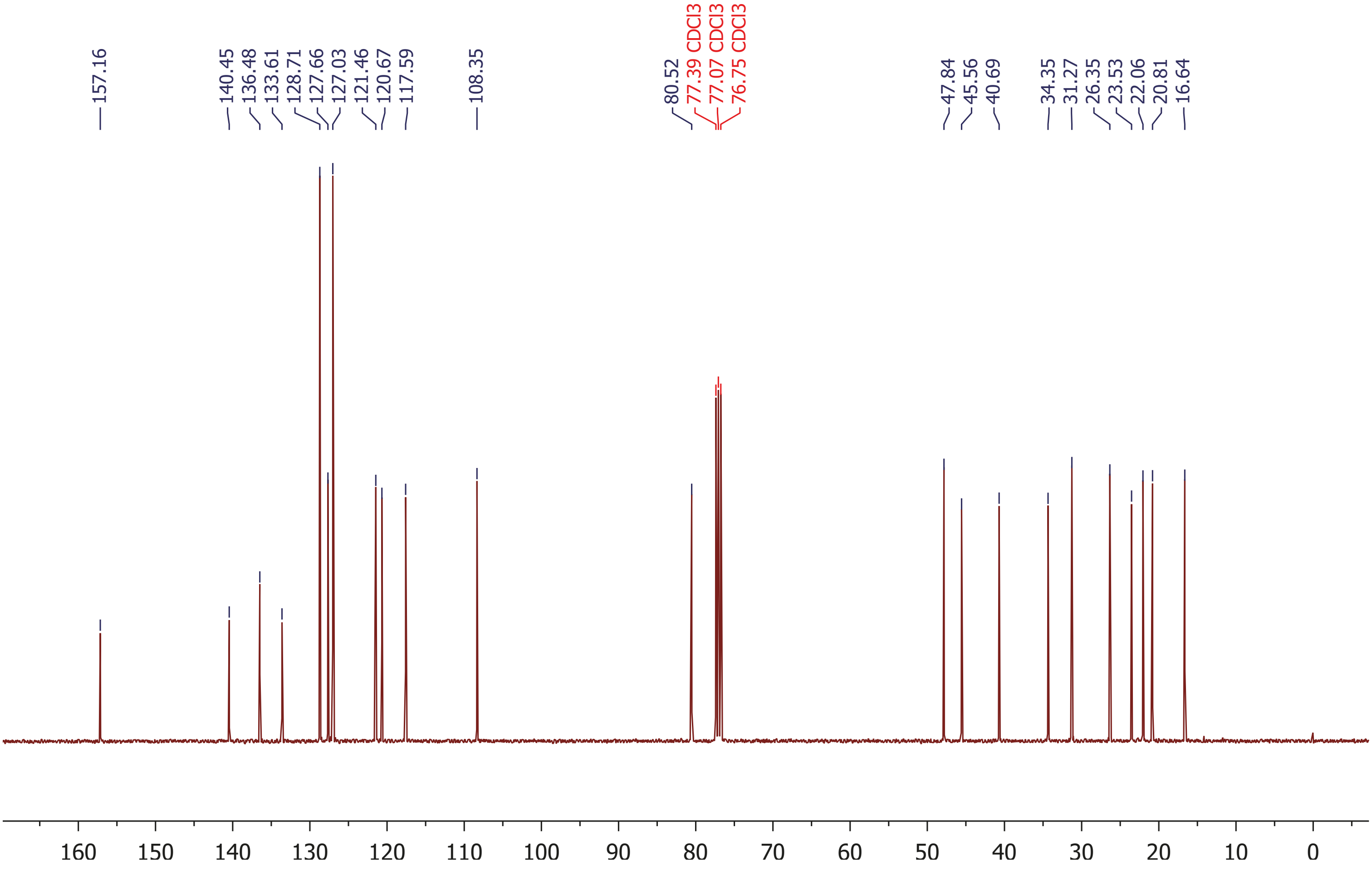
157.16 140.45 136.48 133.61 128.71 127.66 127.03 121.46 120.67 117.59 108.35 80.52
77.39 CDCl3
77.07 CDCl3
76.75 CDCl3
47.84 45.56 40.69 34.35 31.27 26.35 23.53 22.06 20.81 16.64
160 150 140 130 120 110 100 90 80 70 60 50 40 30 20 10 0

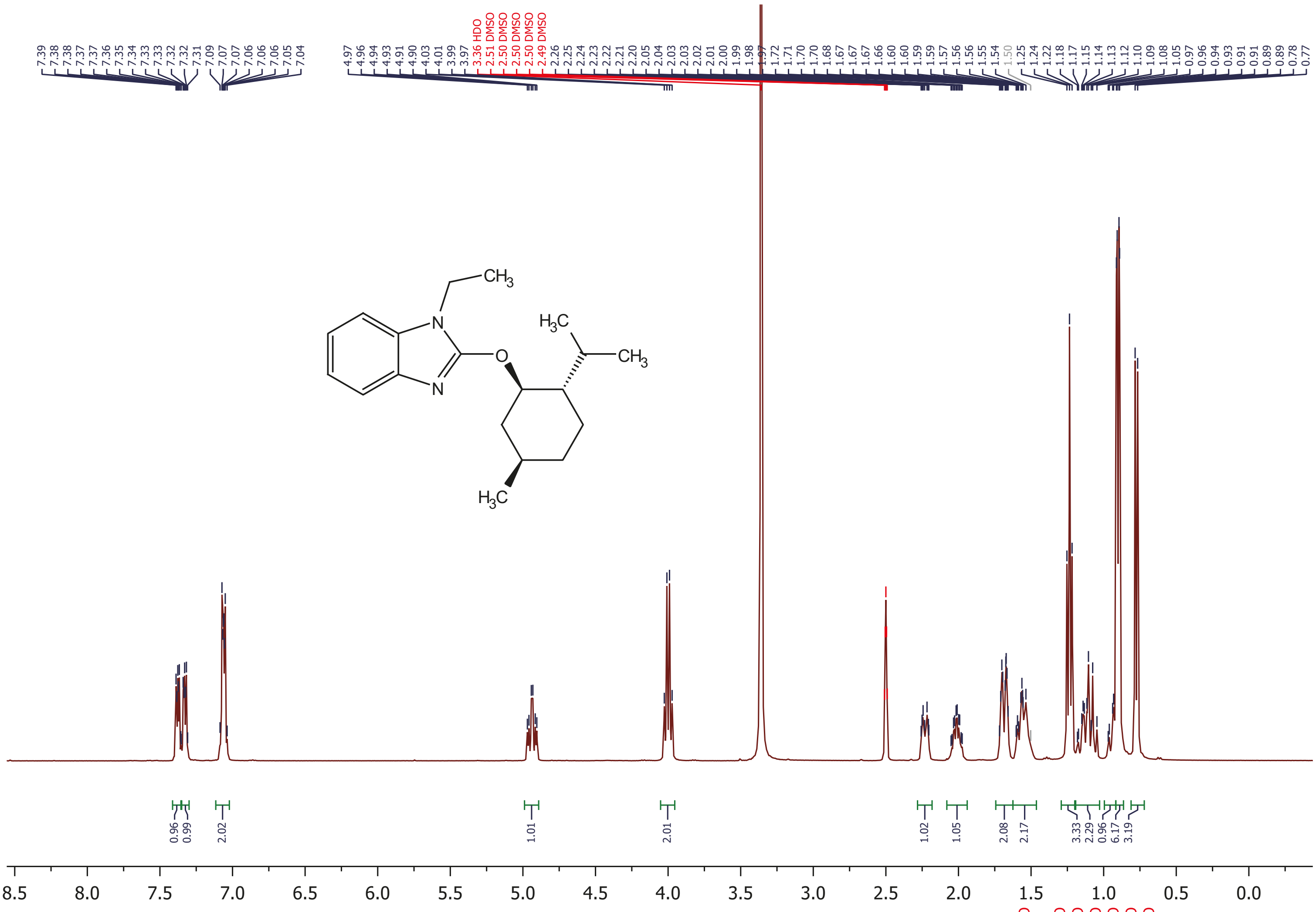

7.39 7.38 7.38 7.37 7.37 7.36 7.35 7.34 7.33 7.33 7.32 7.32 7.31 7.09 7.07 7.07 7.06 7.06 7.06 7.05 7.04
4.97 4.96 4.94 4.93 4.91 4.90 4.03 4.01 3.99 3.97
3.36 HDO
2.51 DMSO
2.50 DMSO
2.50 DMSO
2.50 DMSO
2.49 DMSO
2.26 2.25 2.24 2.23 2.22 2.21 2.20 2.05 2.04 2.03 2.03 2.02 2.01 2.00 1.99 1.98 1.97 1.72 1.71 1.70 1.68 1.67 1.67 1.67 1.66 1.60 1.60 1.59 1.59 1.57 1.56 1.55 1.54 1.50 1.25 1.24 1.22 1.18 1.17 1.15 1.14 1.13 1.12 1.10 1.09 1.08 1.05 0.97 0.96 0.94 0.93 0.91 0.91 0.89 0.78 0.77
CH3
N
N
O
H3C
CH3
H3C
0.96 0.99 2.02 1.01 2.01 1.02 1.05 2.08 2.17 3.33 2.29 0.96 6.17 3.19
8.5 8.0 7.5 7.0 6.5 6.0 5.5 5.0 4.5 4.0 3.5 3.0 2.5 2.0 1.5 1.0 0.5 0.0

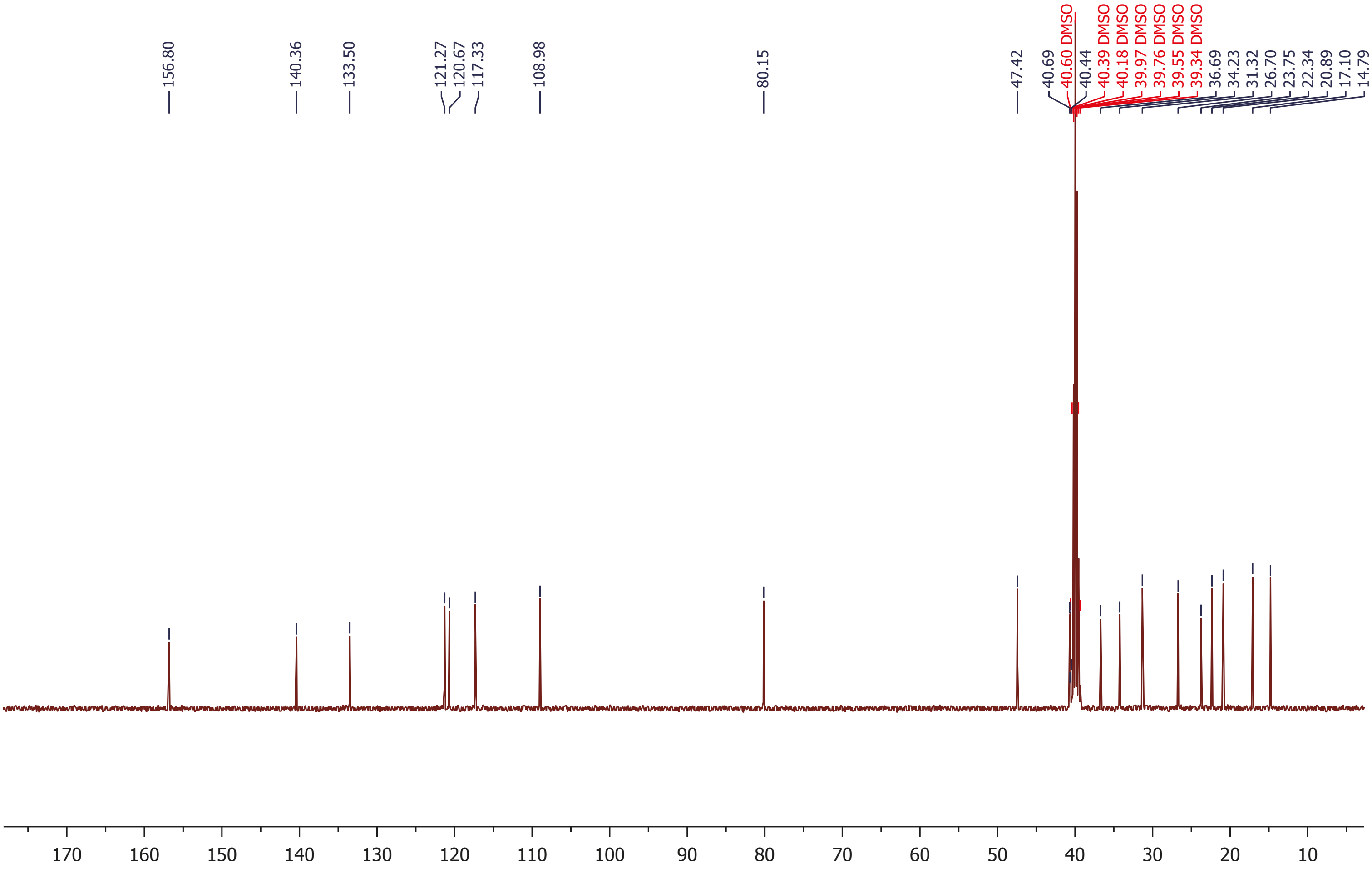

156.80
140.36
133.50
121.27
120.67
117.33
108.98
80.15
47.42
40.69
40.60 DMSO
40.44
40.39 DMSO
40.18 DMSO
39.97 DMSO
39.76 DMSO
39.55 DMSO
39.34 DMSO
36.69
34.23
31.32
26.70
23.75
22.34
20.89
17.10
14.79
170 160 150 140 130 120 110 100 90 80 70 60 50 40 30 20 10

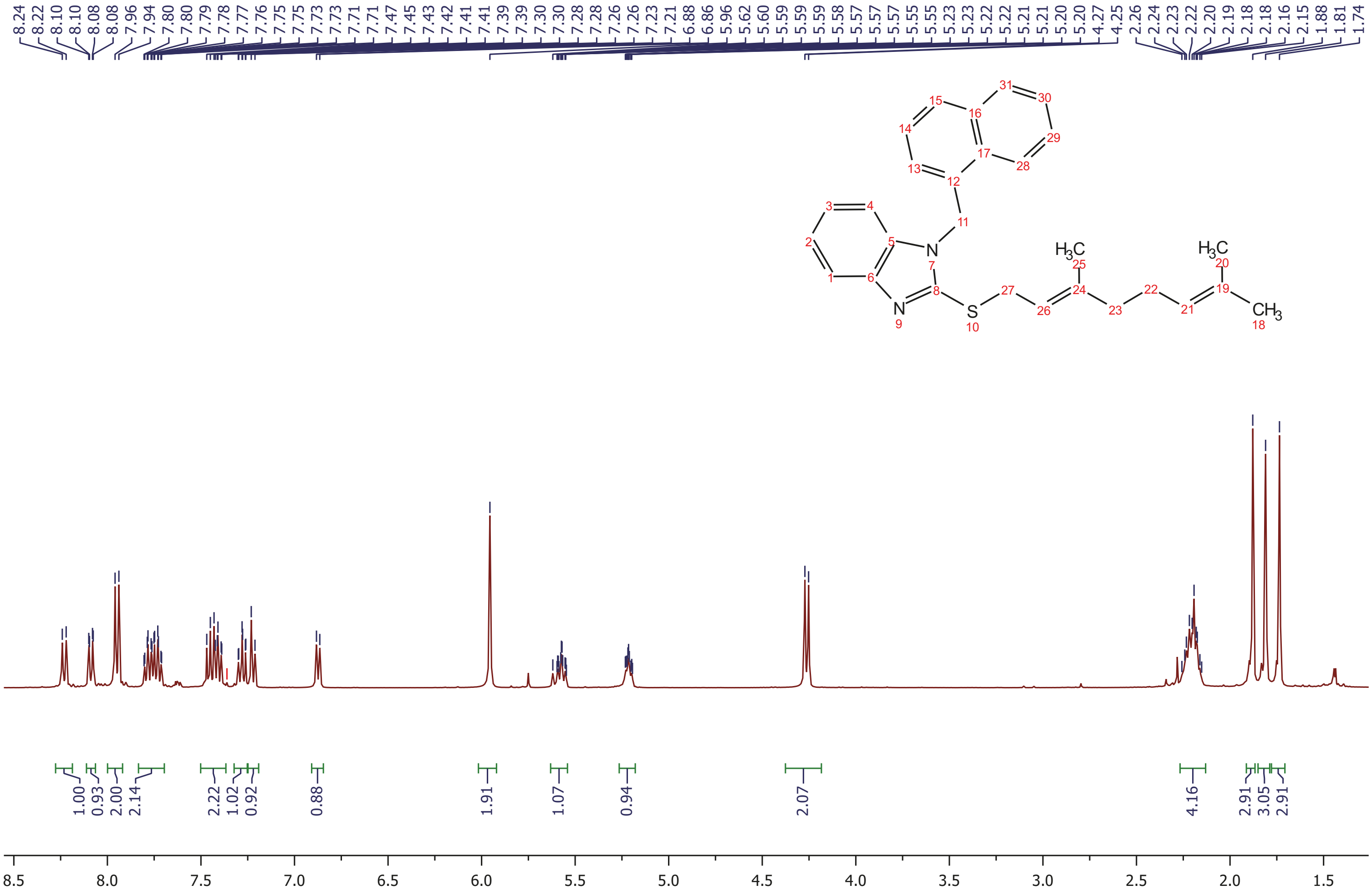
8.24 8.22 8.10 8.10 8.08 8.08 7.96 7.94 7.80 7.80 7.79 7.78 7.77 7.76 7.75 7.75 7.73 7.73 7.71 7.71 7.47 7.45 7.43 7.42 7.41 7.39 7.39 7.30 7.30 7.28 7.28 7.26 7.23 7.21 6.88 6.86 5.96 5.62 5.60 5.59 5.59 5.59 5.58 5.57 5.57 5.57 5.55 5.55 5.23 5.23 5.22 5.22 5.21 5.21 5.20 5.20 4.27 4.25 2.26 2.24 2.23 2.22 2.20 2.19 2.18 2.18 2.16 2.15 1.88 1.81 1.74
1.00 0.93 2.00 2.14 2.22 1.02 0.92 0.88 1.91 1.07 0.94 2.07 4.16 2.91 3.05 2.91
8.5 8.0 7.5 7.0 6.5 6.0 5.5 5.0 4.5 4.0 3.5 3.0 2.5 2.0 1.5
N
S
H_3C
CH_3

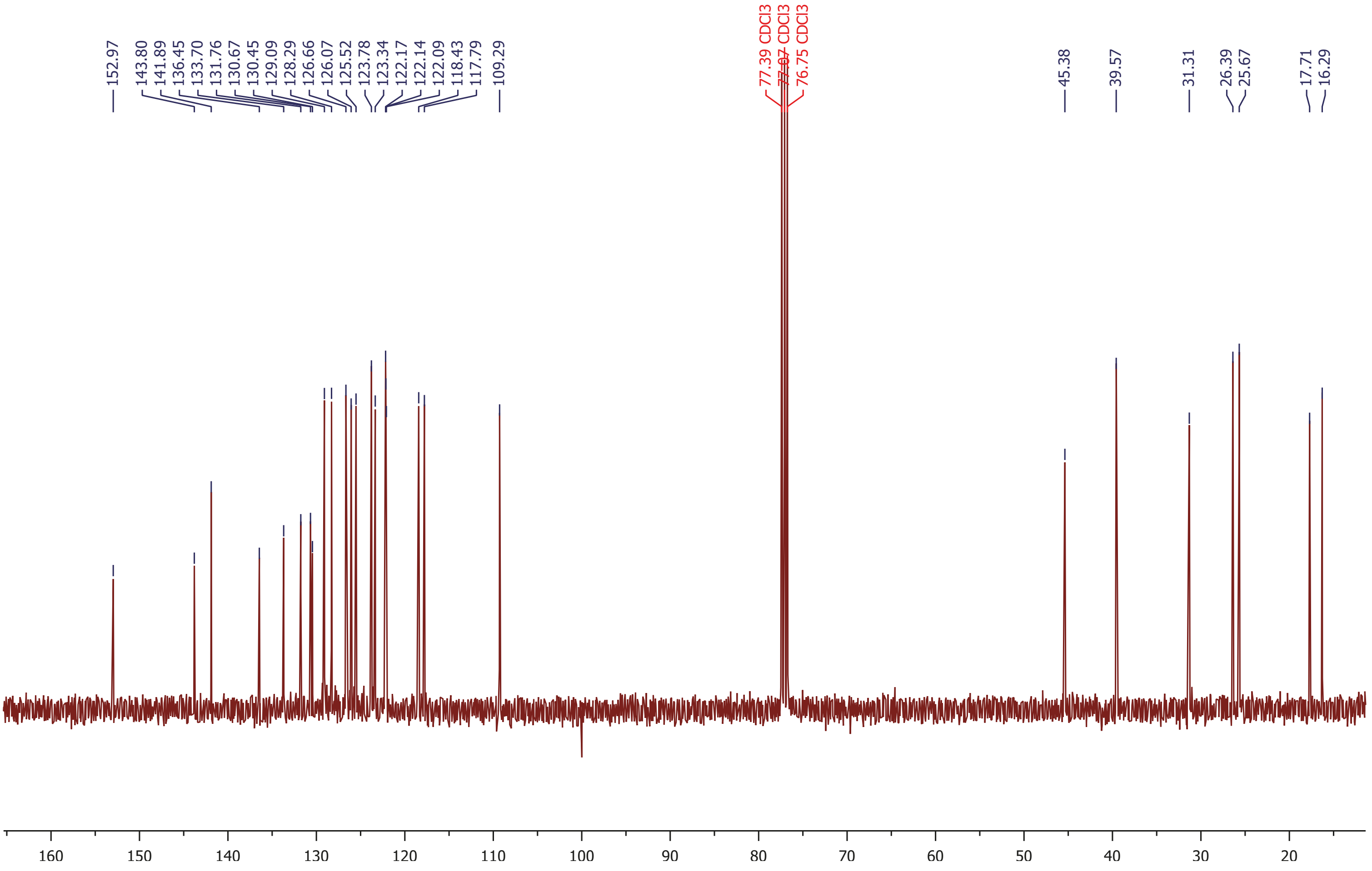
152.97 143.80 141.89 136.45 133.70 131.76 130.67 130.45 129.09 128.29 126.66 126.07 125.52 123.78 123.34 122.17 122.14 122.09 118.43 117.79 109.29
77.39 CDCl3 77.07 CDCl3 76.75 CDCl3
45.38 39.57 31.31 26.39 25.67 17.71 16.29
160 150 140 130 120 110 100 90 80 70 60 50 40 30 20

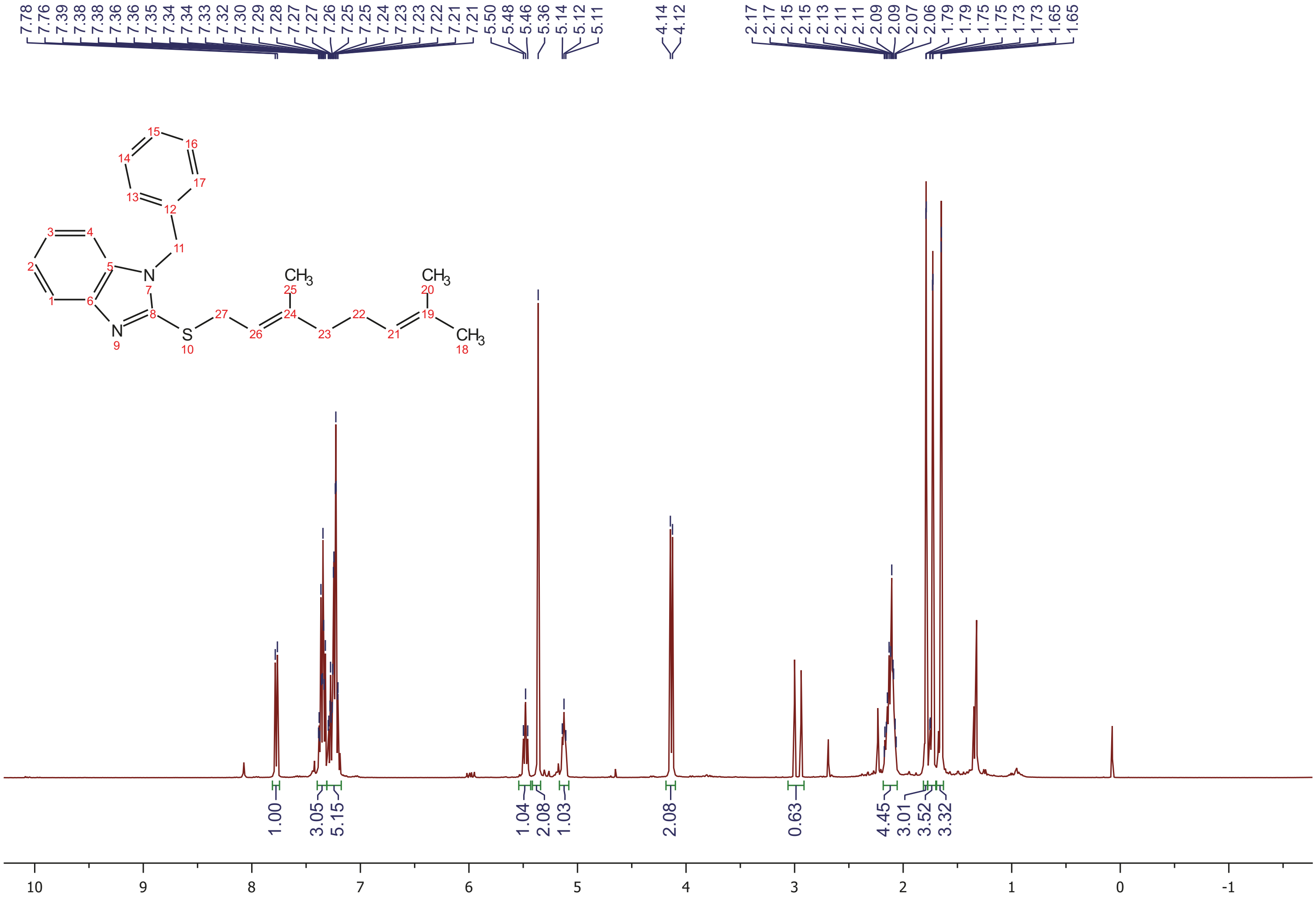
7.78 7.76 7.39 7.38 7.38 7.36 7.36 7.35 7.34 7.34 7.33 7.32 7.30 7.29 7.28 7.27 7.27 7.26 7.25 7.25 7.24 7.23 7.23 7.22 7.21 7.21
5.50 5.48 5.46 5.36 5.14 5.12 5.11
4.14 4.12
2.17 2.17 2.15 2.15 2.13 2.11 2.11 2.09 2.09 2.07 2.06 1.79 1.79 1.75 1.75 1.73 1.73 1.65 1.65
1.00 3.05 5.15 1.04 2.08 1.03 2.08 0.63 4.45 3.01 3.52 3.32
10 9 8 7 6 5 4 3 2 1 0 -1
N
N
S
CH3
CH3
CH3

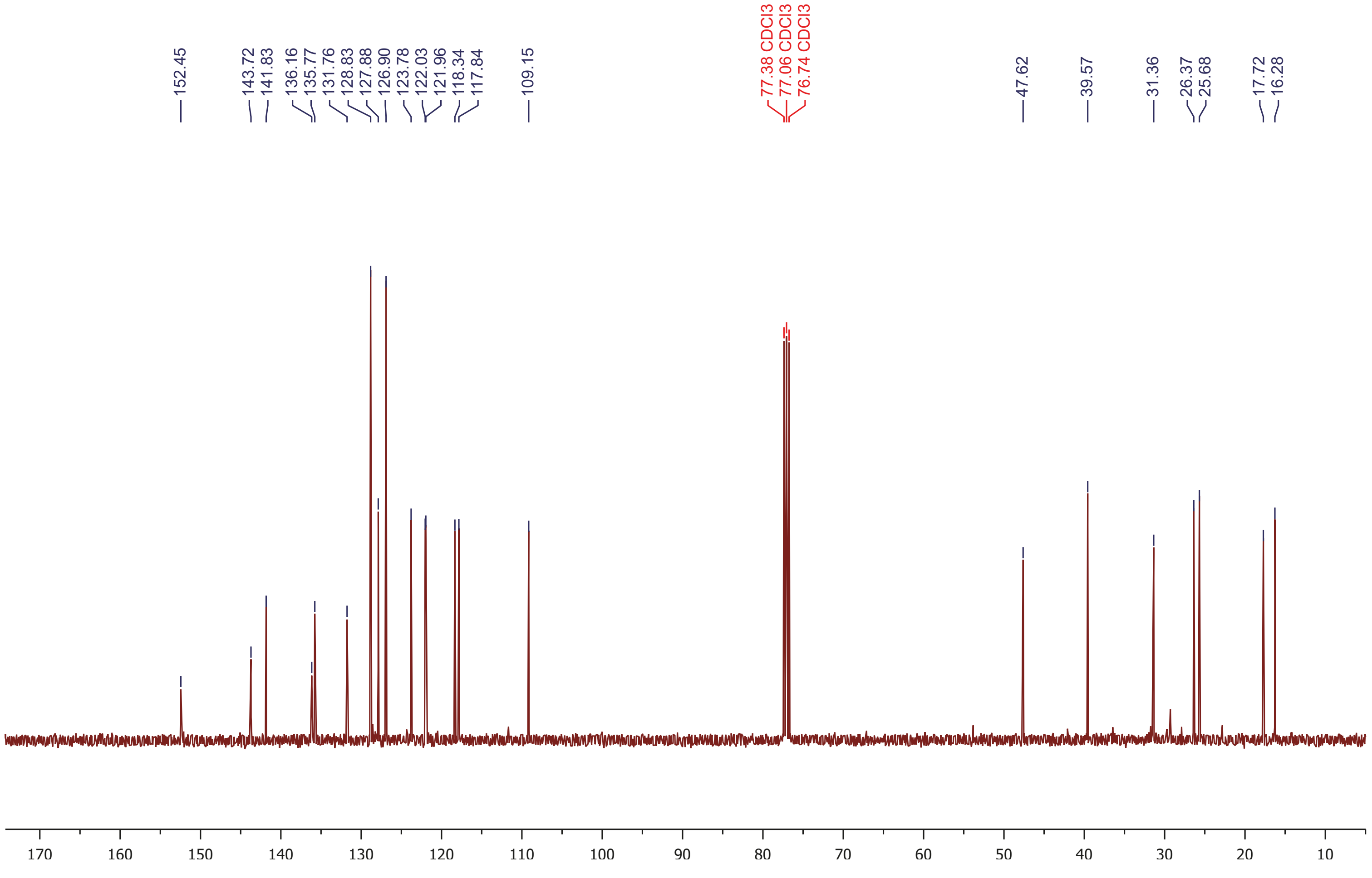
152.45
143.72 141.83 136.16 135.77 131.76 128.83 127.88 126.90 123.78 122.03 121.96 118.34 117.84
109.15
77.38 CDCl3
77.06 CDCl3
76.74 CDCl3
47.62 39.57 31.36 26.37 25.68 17.72 16.28
170 160 150 140 130 120 110 100 90 80 70 60 50 40 30 20 10

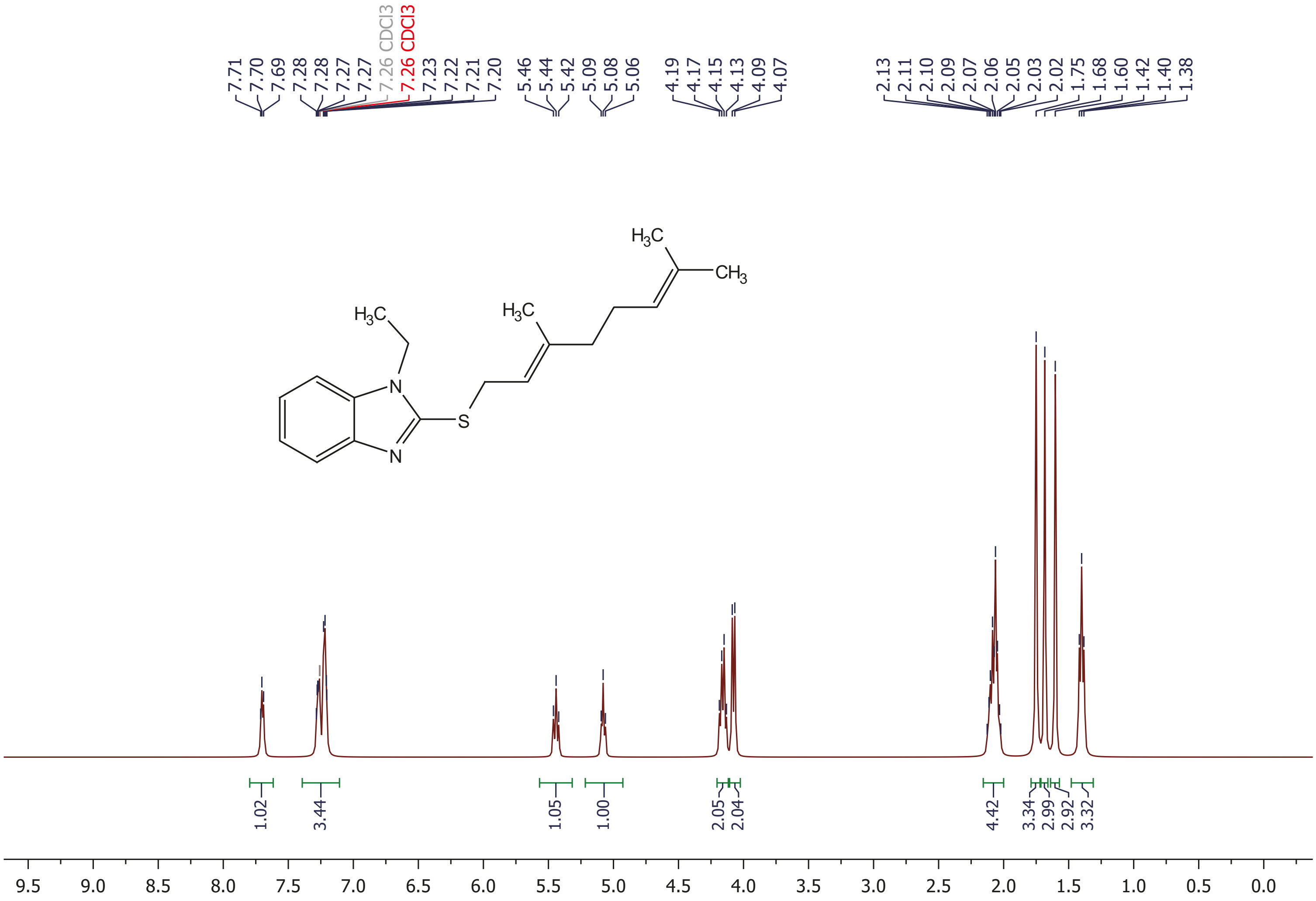
7.71 7.70 7.69 7.28 7.28 7.27 7.27 7.26 CDCl3 7.26 CDCl3 7.23 7.22 7.21 7.20 5.46 5.44 5.42 5.09 5.08 5.06 4.19 4.17 4.15 4.13 4.09 4.07 2.13 2.11 2.10 2.09 2.07 2.06 2.05 2.03 2.02 1.75 1.68 1.60 1.42 1.40 1.38
H3C
CH3
H3C
H3C
N
S
N
1.02 3.44 1.05 1.00 2.05 2.04 4.42 3.34 2.99 2.92 3.32
9.5 9.0 8.5 8.0 7.5 7.0 6.5 6.0 5.5 5.0 4.5 4.0 3.5 3.0 2.5 2.0 1.5 1.0 0.5 0.0
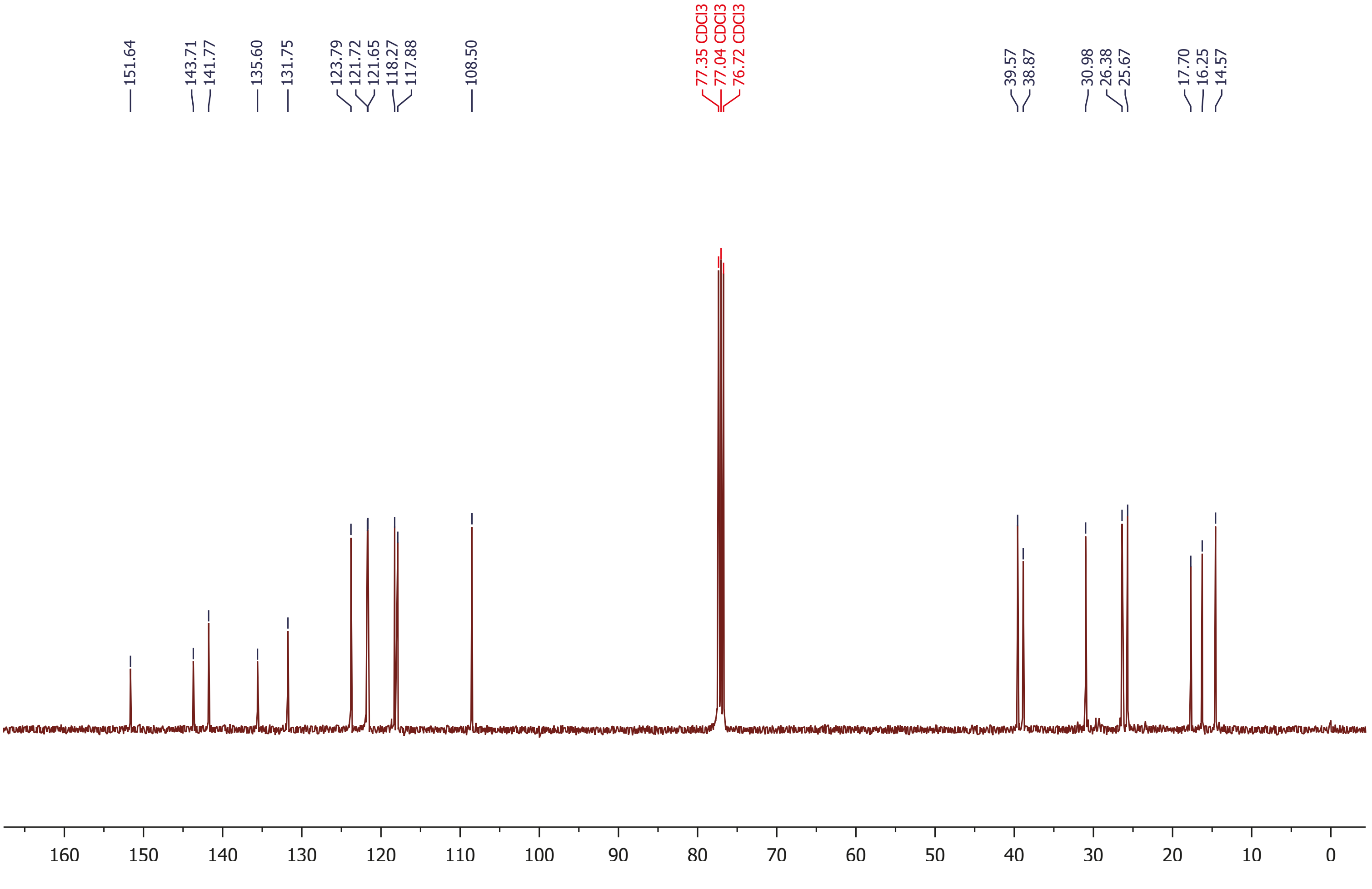
151.64 143.71 141.77 135.60 131.75 123.79 121.72 121.65 118.27 117.88 108.50 77.35 CDCl3 77.04 CDCl3 76.72 CDCl3 39.57 38.87 30.98 26.38 25.67 17.70 16.25 14.57
160 150 140 130 120 110 100 90 80 70 60 50 40 30 20 10 0

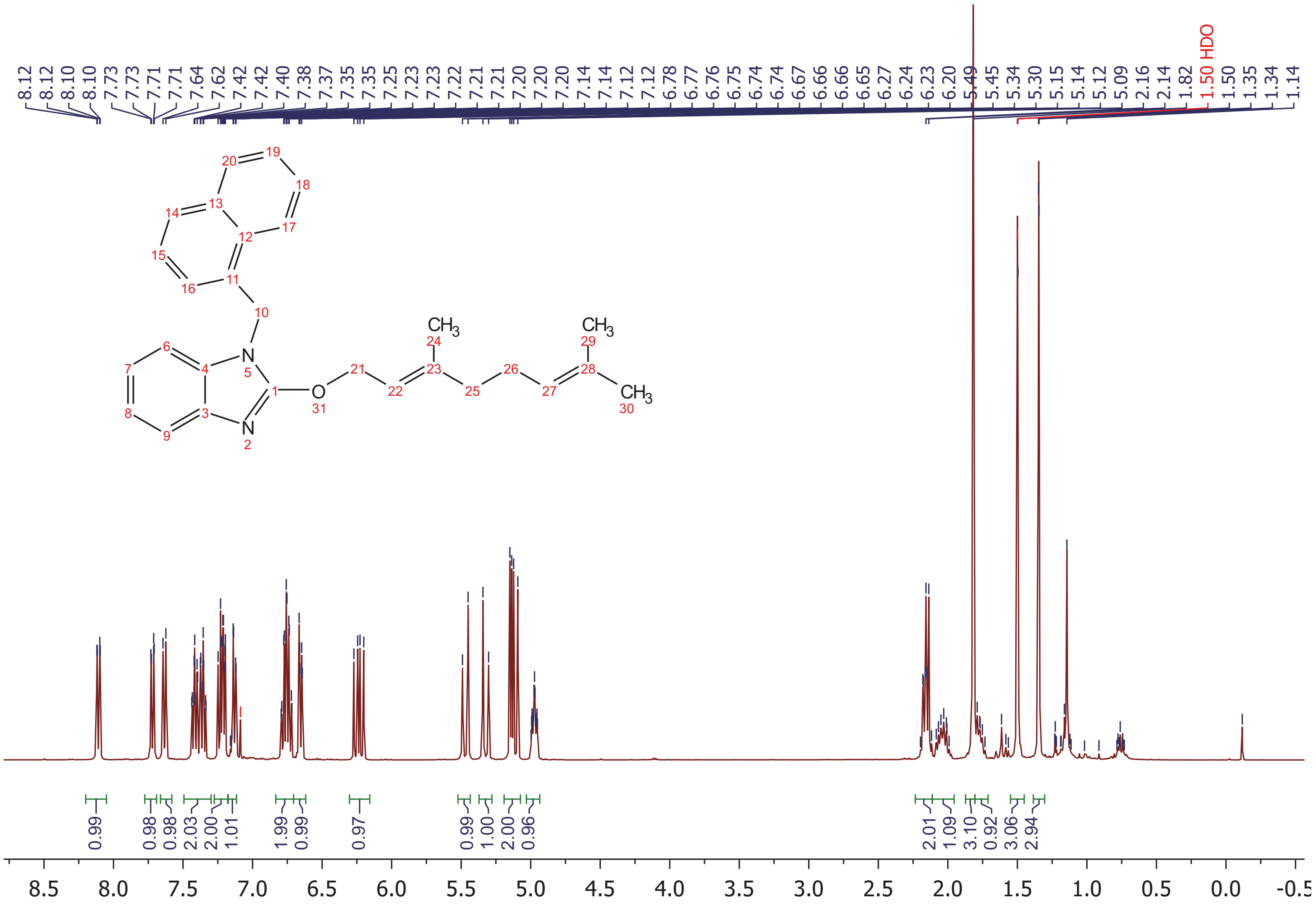

8.12
8.12
8.10
8.10
7.73
7.73
7.71
7.71
7.64
7.62
7.42
7.42
7.40
7.38
7.37
7.35
7.35
7.25
7.23
7.23
7.22
7.21
7.21
7.20
7.20
7.20
7.14
7.14
7.12
7.12
6.78
6.77
6.76
6.75
6.74
6.74
6.67
6.66
6.65
6.27
6.24
6.23
6.20
5.49
5.45
5.34
5.30
5.15
5.14
5.12
5.09
2.16
2.14
1.82
1.50 HDO
1.50
1.35
1.34
1.14
CH3
CH3
CH3
N
N
O
0.99
0.98
0.98
2.03
2.00
1.01
1.99
0.99
0.97
0.99
1.00
2.00
0.96
2.01
1.09
3.10
0.92
3.06
2.94
8.5
8.0
7.5
7.0
6.5
6.0
5.5
5.0
4.5
4.0
3.5
3.0
2.5
2.0
1.5
1.0
0.5
0.0
-0.5

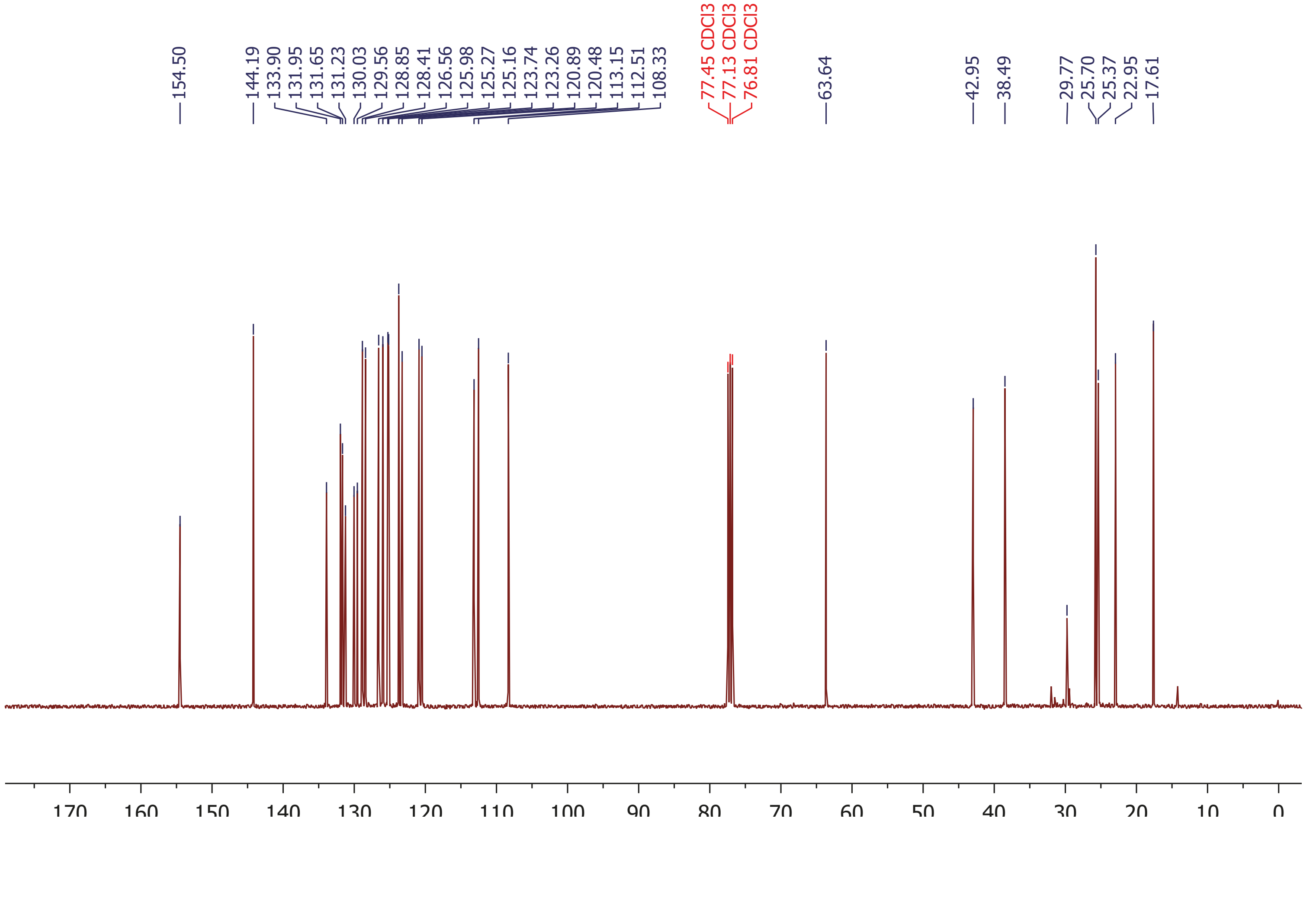

154.50
144.19
133.90
131.95
131.65
131.23
130.03
129.56
128.85
128.41
126.56
125.98
125.27
125.16
123.74
123.26
120.89
120.48
113.15
112.51
108.33
77.45 CDCl3
77.13 CDCl3
76.81 CDCl3
63.64
42.95
38.49
29.77
25.70
25.37
22.95
17.61
170
160
150
140
130
120
110
100
90
80
70
60
50
40
30
20
10
0

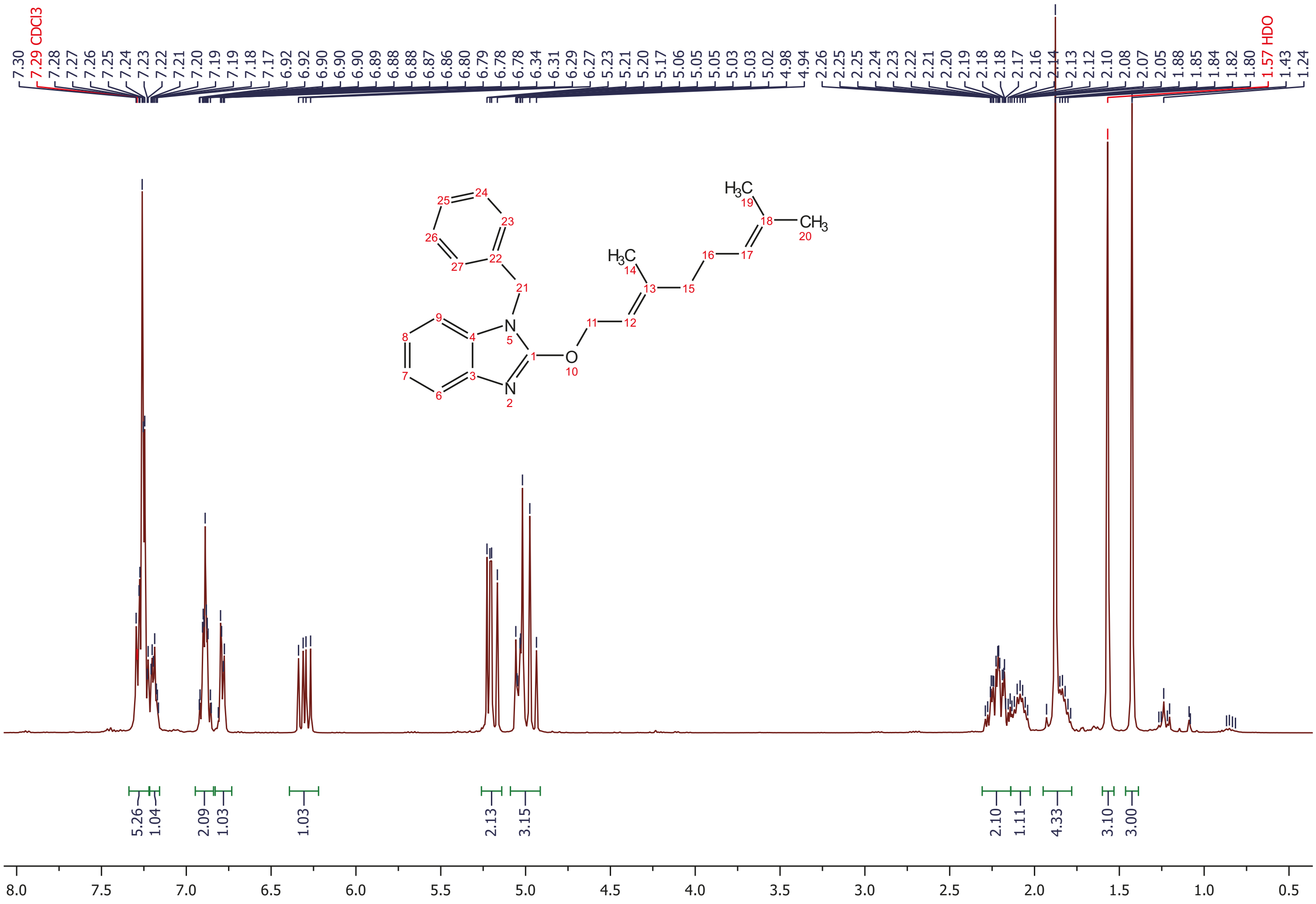

7.30
7.29 CDCl3
7.28
7.27
7.26
7.25
7.24
7.23
7.22
7.21
7.20
7.19
7.19
7.18
7.17
6.92
6.92
6.90
6.90
6.90
6.89
6.88
6.88
6.87
6.86
6.80
6.79
6.78
6.78
6.34
6.31
6.29
6.27
5.23
5.21
5.20
5.17
5.06
5.05
5.05
5.03
5.03
5.02
4.98
4.94
2.26
2.25
2.25
2.24
2.23
2.22
2.21
2.20
2.19
2.18
2.18
2.17
2.16
2.14
2.13
2.12
2.10
2.08
2.07
2.05
1.88
1.85
1.84
1.82
1.80
1.57 HDO
1.43
1.24
5.26
1.04
2.09
1.03
1.03
2.13
3.15
2.10
1.11
4.33
3.10
3.00
8.0
7.5
7.0
6.5
6.0
5.5
5.0
4.5
4.0
3.5
3.0
2.5
2.0
1.5
1.0
0.5

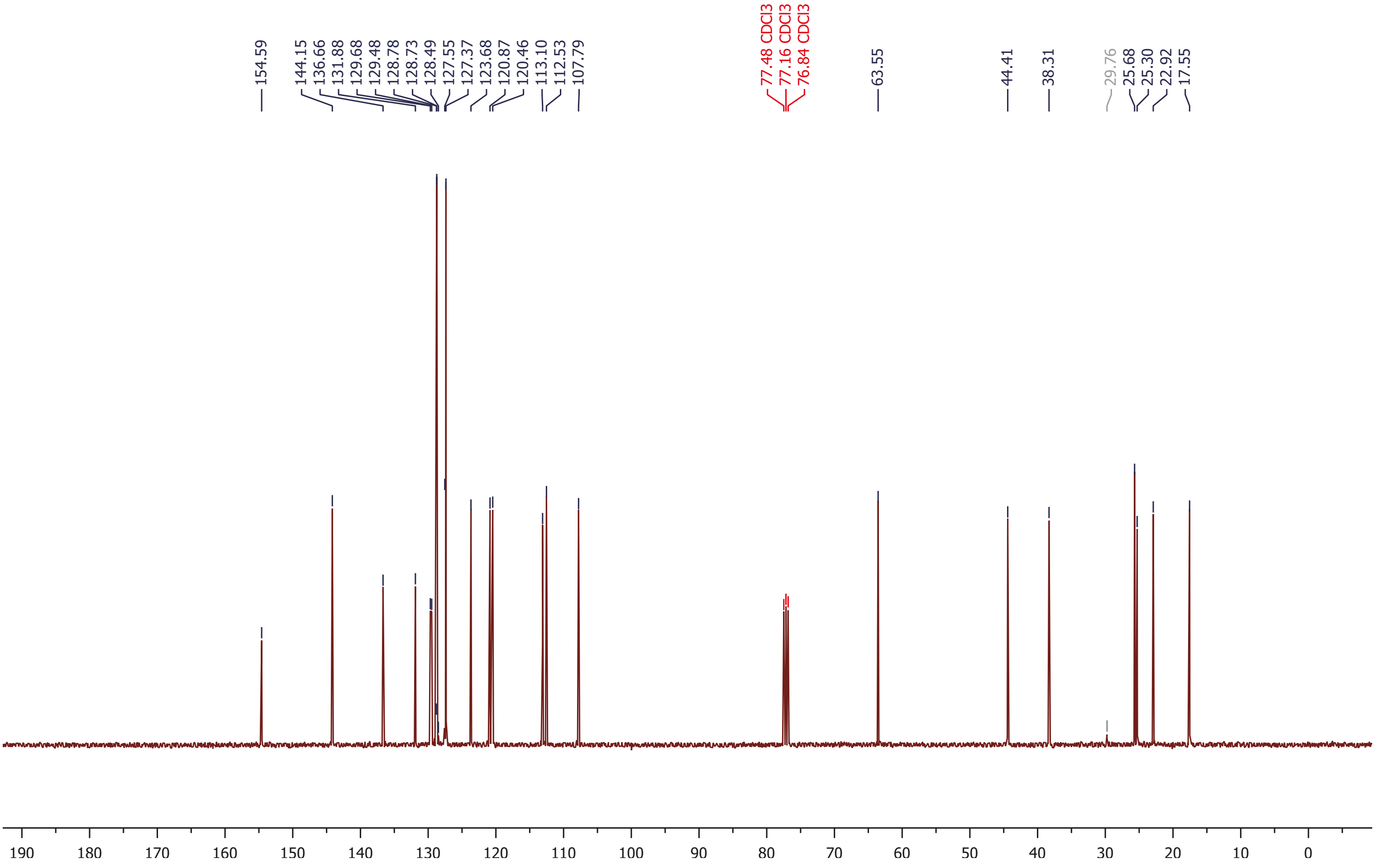

154.59
144.15
136.66
131.88
129.68
129.48
128.78
128.73
128.49
127.55
127.37
123.68
120.87
120.46
113.10
112.53
107.79
77.48 CDCl3
77.16 CDCl3
76.84 CDCl3
63.55
44.41
38.31
29.76
25.68
25.30
22.92
17.55
190
180
170
160
150
140
130
120
110
100
90
80
70
60
50
40
30
20
10
0

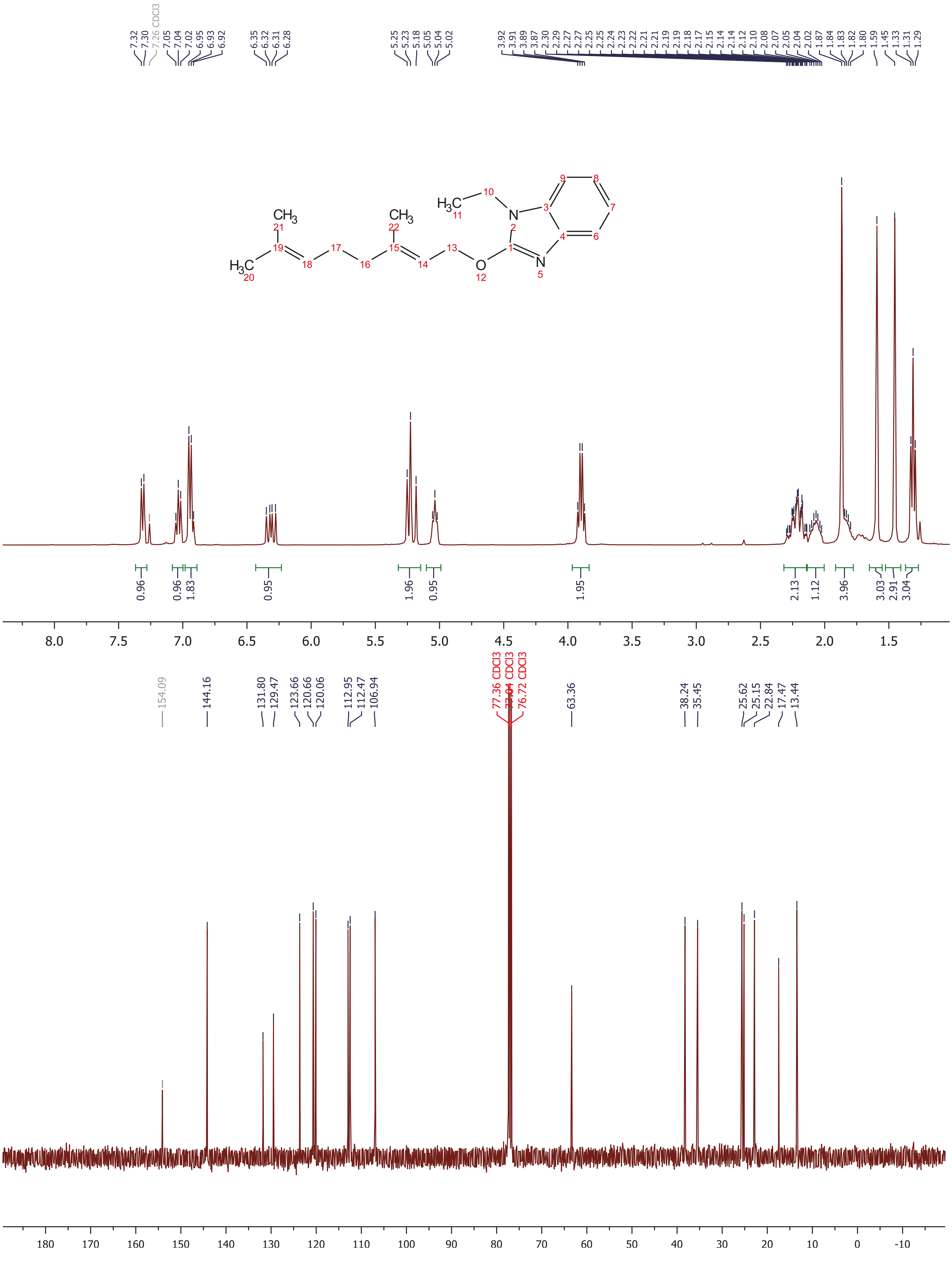

7.32 7.30 7.26 CDCl3 7.05 7.04 7.02 6.95 6.93 6.92 6.35 6.32 6.31 6.28 5.25 5.23 5.18 5.05 5.04 5.02 3.92 3.91 3.89 3.87 2.30 2.29 2.27 2.27 2.25 2.25 2.24 2.23 2.22 2.21 2.21 2.19 2.19 2.18 2.17 2.15 2.14 2.14 2.12 2.10 2.08 2.07 2.05 2.04 2.02 1.87 1.84 1.83 1.82 1.80 1.59 1.45 1.33 1.31 1.29
0.96 0.96 1.83 0.95 1.96 0.95 1.95 2.13 1.12 3.96 3.03 2.91 3.04
154.09 144.16 131.80 129.47 123.66 120.66 120.06 112.95 112.47 106.94 77.36 CDCl3 77.04 CDCl3 76.72 CDCl3 63.36 38.24 35.45 25.62 25.15 22.84 17.47 13.44

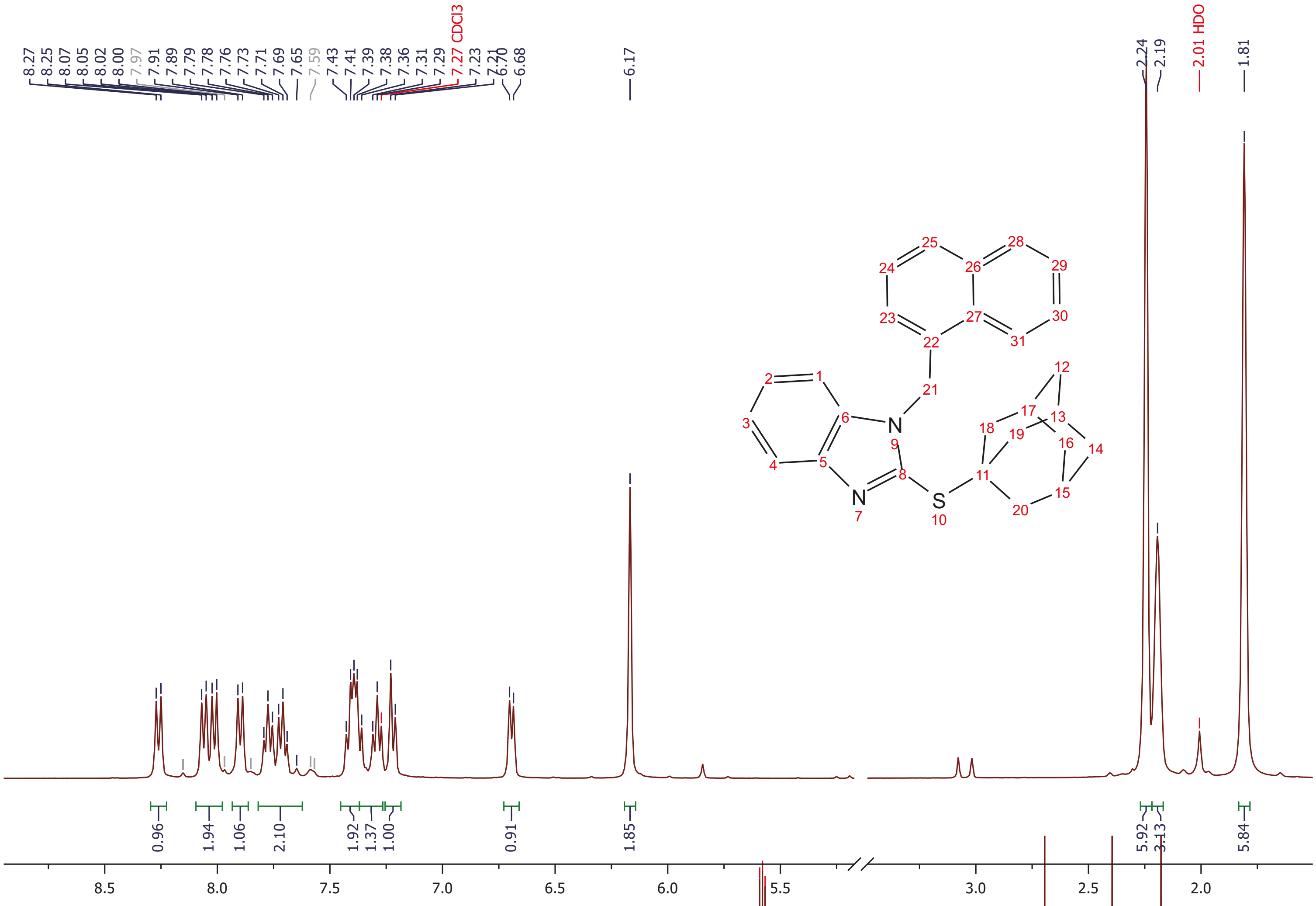
8.27
8.25
8.07
8.05
8.02
8.00
7.97
7.91
7.89
7.79
7.78
7.76
7.73
7.71
7.69
7.65
7.59
7.43
7.41
7.39
7.38
7.36
7.31
7.29
7.27 CDCl3
7.23
7.21
6.70
6.68
6.17
2.24
2.19
2.01 HDO
1.81
0.96
1.94
1.06
2.10
1.92
1.37
1.00
0.91
1.85
5.92
3.13
5.84
8.5
8.0
7.5
7.0
6.5
6.0
5.5
3.0
2.5
2.0
S
N
N
1
2
3
4
5
6
7
8
9
10
11
12
13
14
15
16
17
18
19
20
21
22
23
24
25
26
27
28
29
30
31

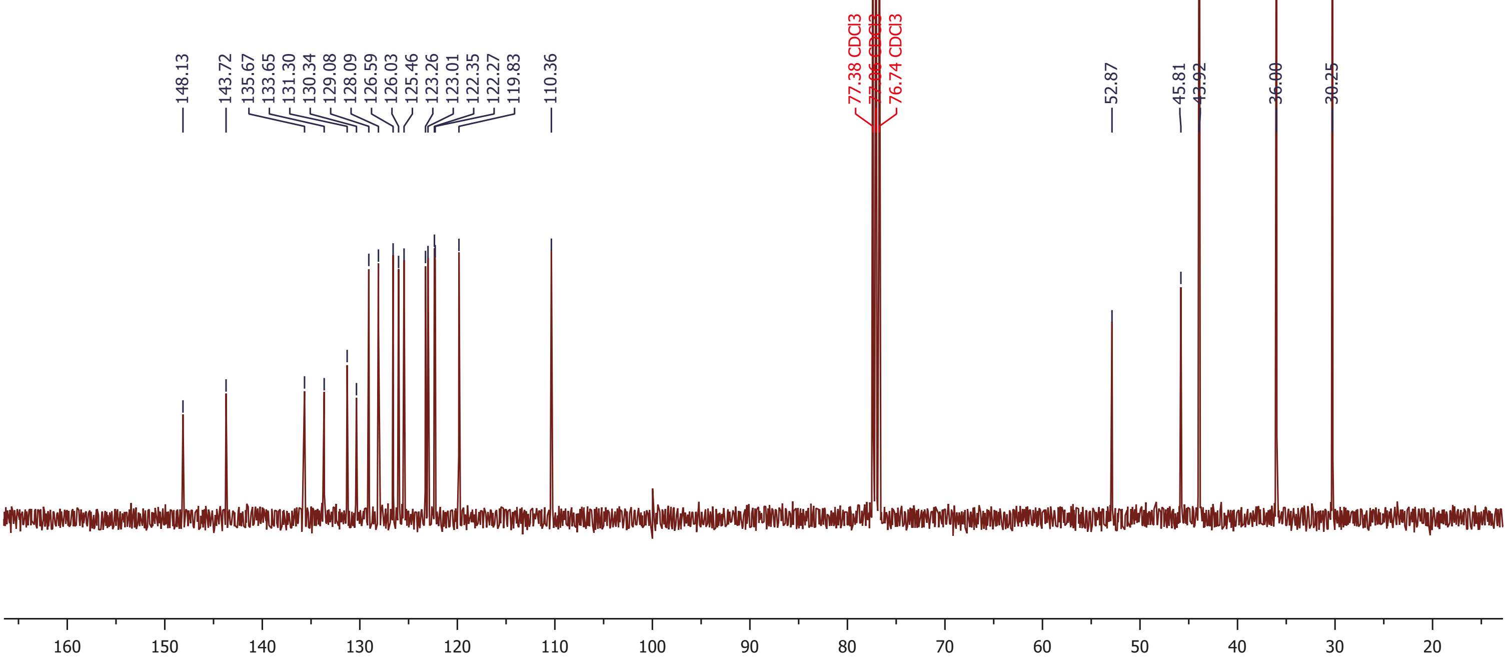
148.13
143.72
135.67
133.65
131.30
130.34
129.08
128.09
126.59
126.03
125.46
123.26
123.01
122.35
122.27
119.83
110.36
77.38 CDCl3
76.74 CDCl3
52.87
45.81
43.92
36.00
30.25
160
150
140
130
120
110
100
90
80
70
60
50
40
30
20

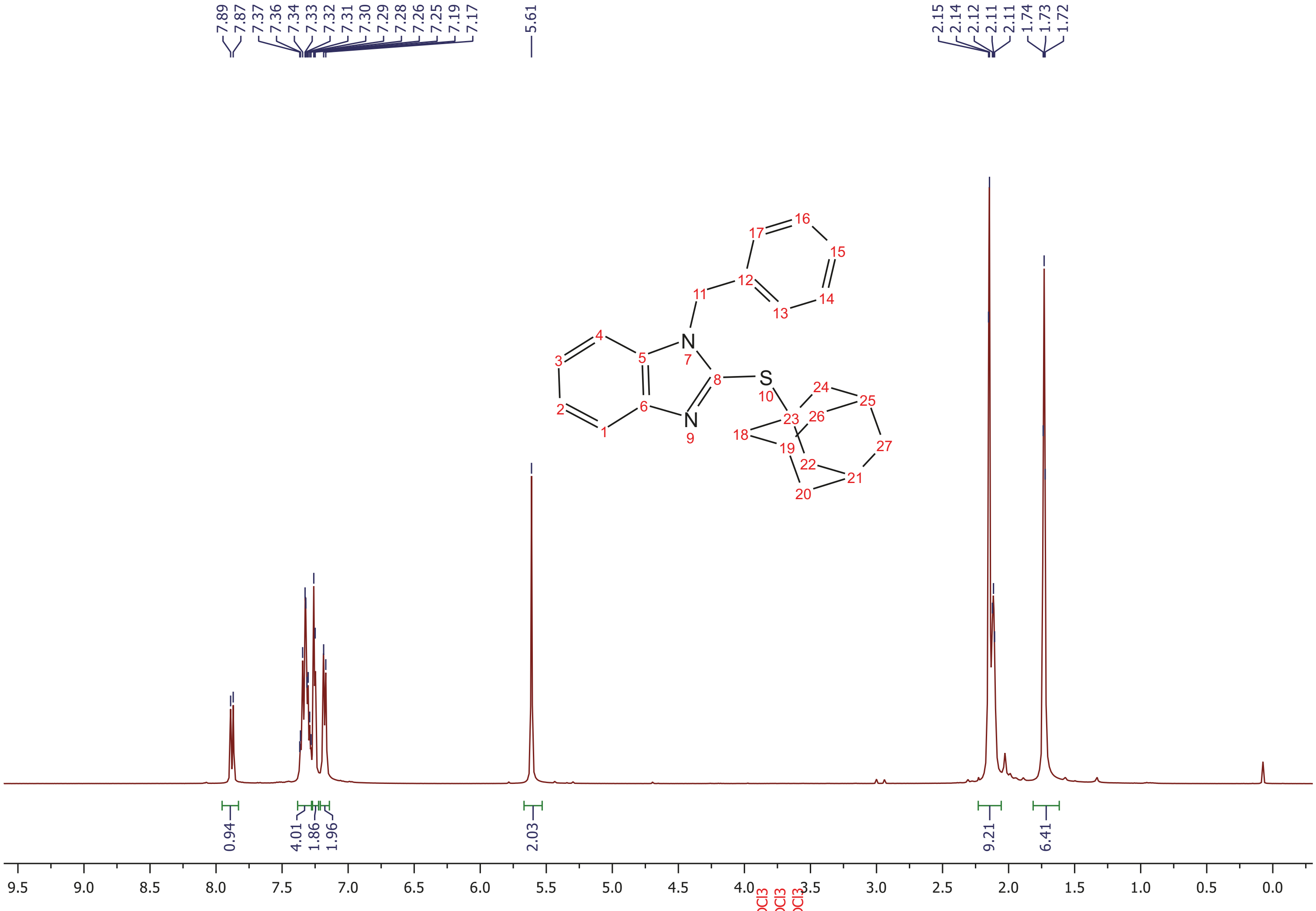
7.89
7.87
7.37
7.36
7.34
7.33
7.32
7.31
7.30
7.29
7.28
7.26
7.25
7.19
7.17
5.61
2.15
2.14
2.12
2.11
2.11
1.74
1.73
1.72
0.94
4.01
1.86
1.96
2.03
9.21
6.41
9.5
9.0
8.5
8.0
7.5
7.0
6.5
6.0
5.5
5.0
4.5
4.0
3.5
3.0
2.5
2.0
1.5
1.0
0.5
0.0
N
S
N
1
2
3
4
5
6
7
8
9
10
11
12
13
14
15
16
17
18
19
20
21
22
23
24
25
26
27

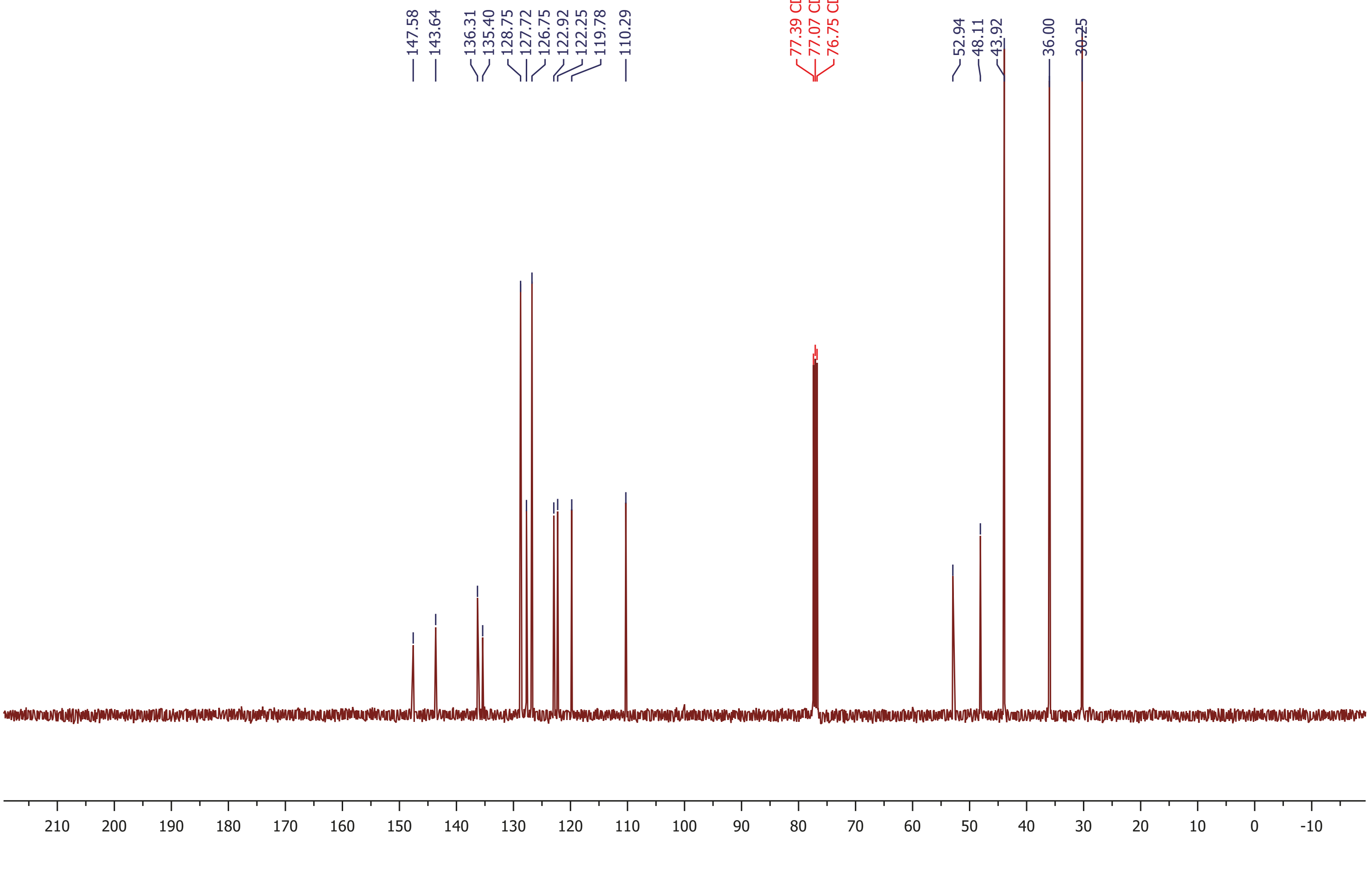
147.58
143.64
136.31
135.40
128.75
127.72
126.75
122.92
122.25
119.78
110.29
77.39 CDCl3
77.07 CDCl3
76.75 CDCl3
52.94
48.11
43.92
36.00
30.25
210
200
190
180
170
160
150
140
130
120
110
100
90
80
70
60
50
40
30
20
10
0
-10

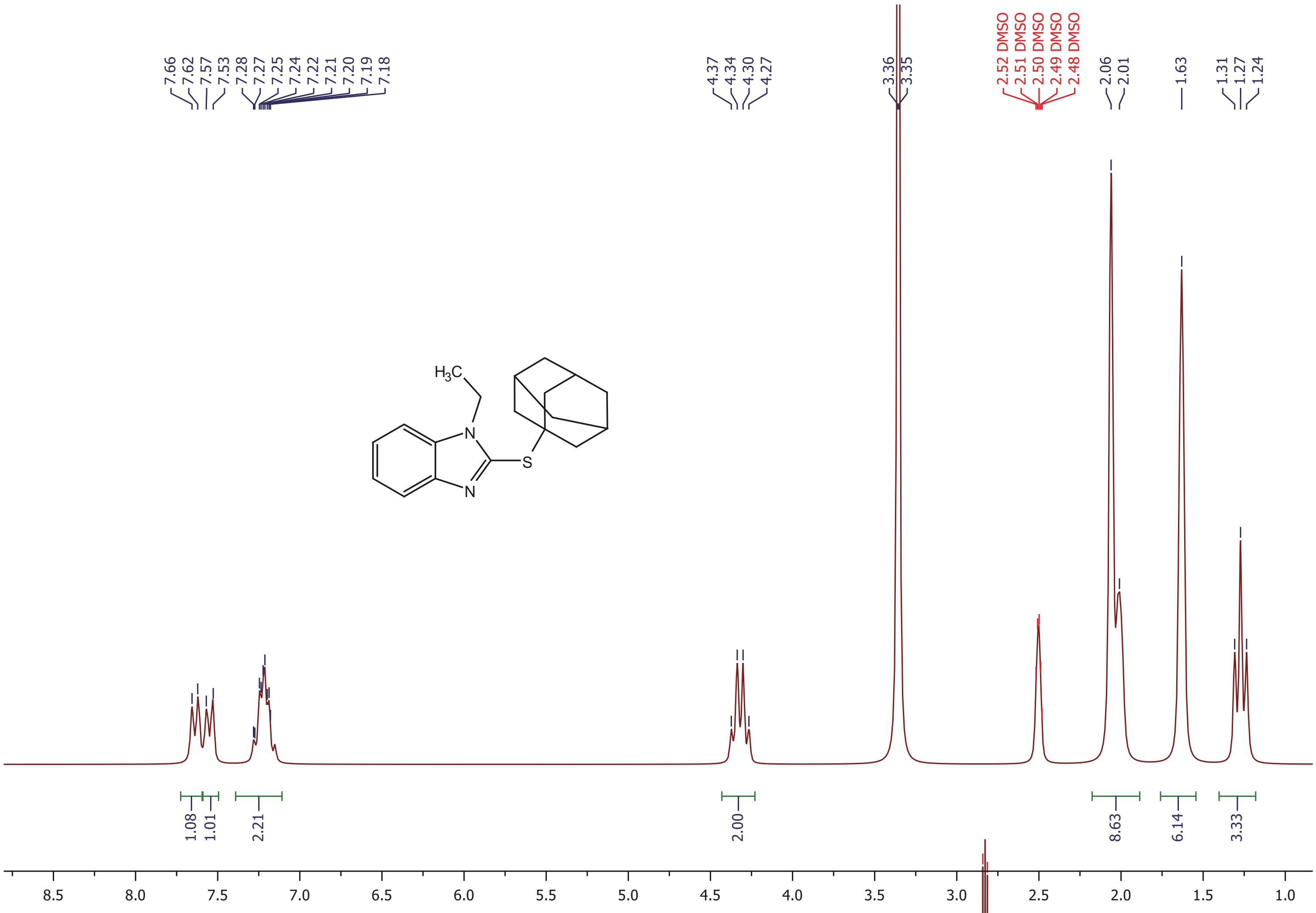
7.66
7.62
7.57
7.53
7.28
7.27
7.25
7.24
7.22
7.21
7.20
7.19
7.18
4.37
4.34
4.30
4.27
3.36
3.35
2.52 DMSO
2.51 DMSO
2.50 DMSO
2.49 DMSO
2.48 DMSO
2.06
2.01
1.63
1.31
1.27
1.24
1.08
1.01
2.21
2.00
8.63
6.14
3.33
8.5
8.0
7.5
7.0
6.5
6.0
5.5
5.0
4.5
4.0
3.5
3.0
2.5
2.0
1.5
1.0
H3C
N
N
S

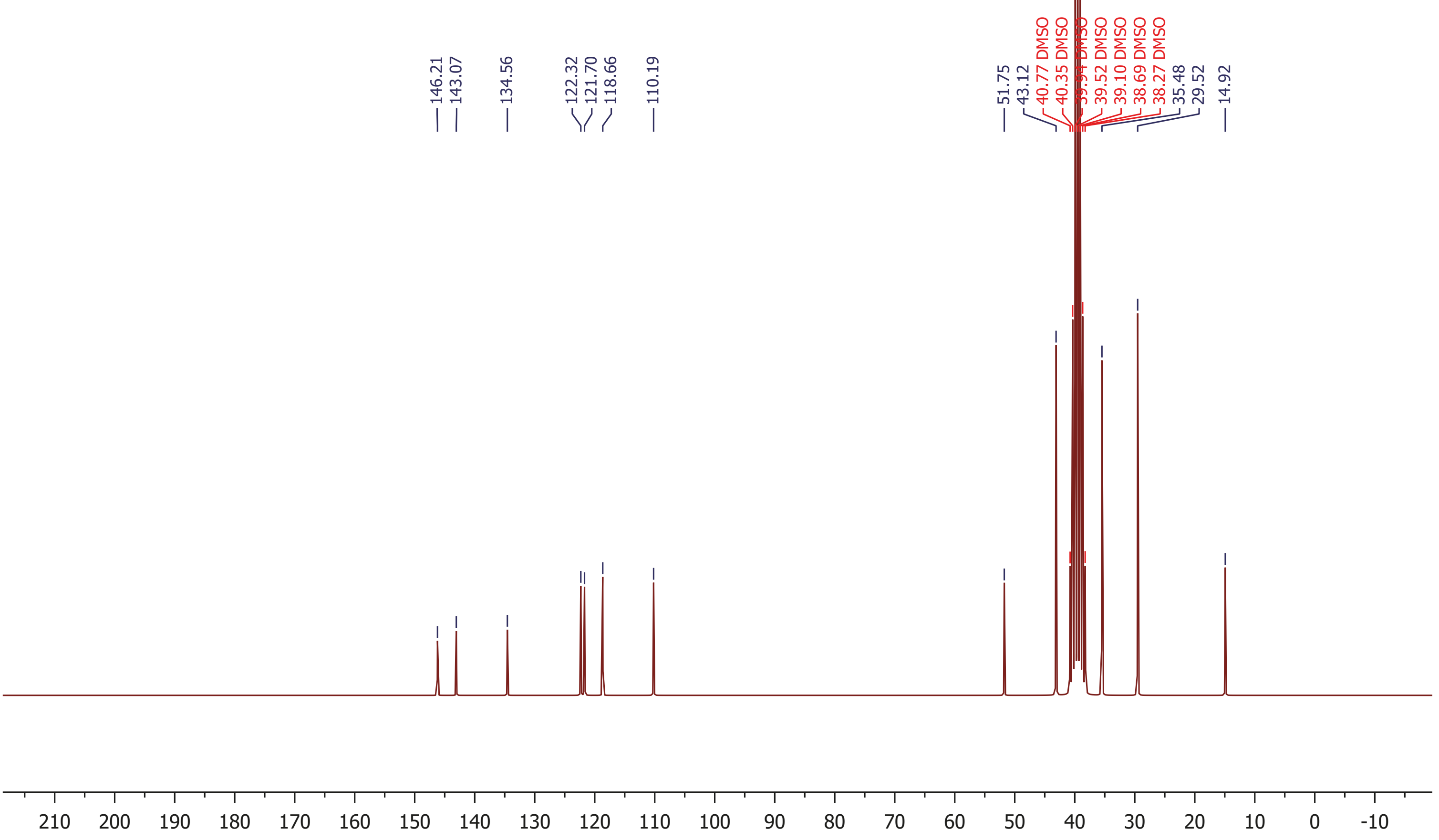
146.21
143.07
134.56
122.32
121.70
118.66
110.19
51.75
43.12
40.77 DMSO
40.35 DMSO
39.52 DMSO
39.10 DMSO
38.69 DMSO
38.27 DMSO
35.48
29.52
14.92
210
200
190
180
170
160
150
140
130
120
110
100
90
80
70
60
50
40
30
20
10
0
-10

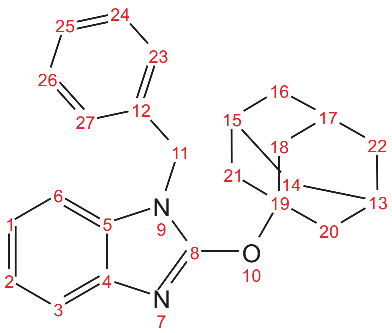
N
N
O
1 2 3 4 5 6 7 8 9 10 11 12 13 14 15 16 17 18 19 20 21 22 23 24 25 26 27

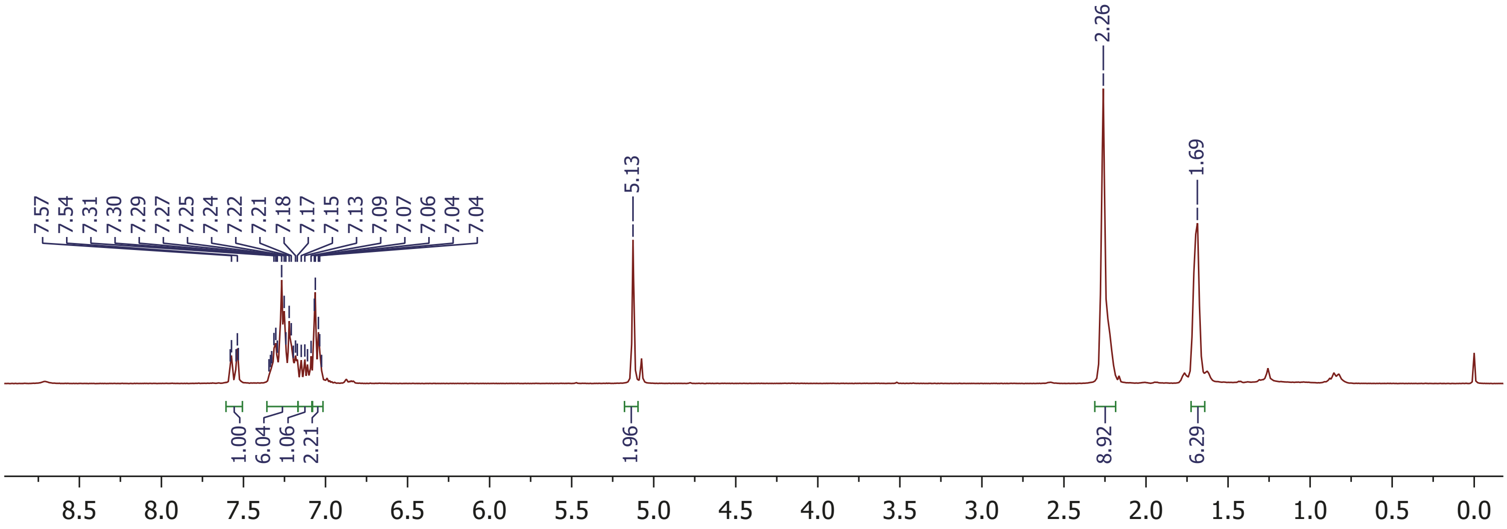
7.57 7.54 7.31 7.30 7.29 7.27 7.25 7.24 7.22 7.21 7.18 7.17 7.15 7.13 7.09 7.07 7.06 7.04 7.04
5.13
2.26
1.69
1.00 6.04 1.06 2.21
1.96
8.92
6.29
8.5 8.0 7.5 7.0 6.5 6.0 5.5 5.0 4.5 4.0 3.5 3.0 2.5 2.0 1.5 1.0 0.5 0.0

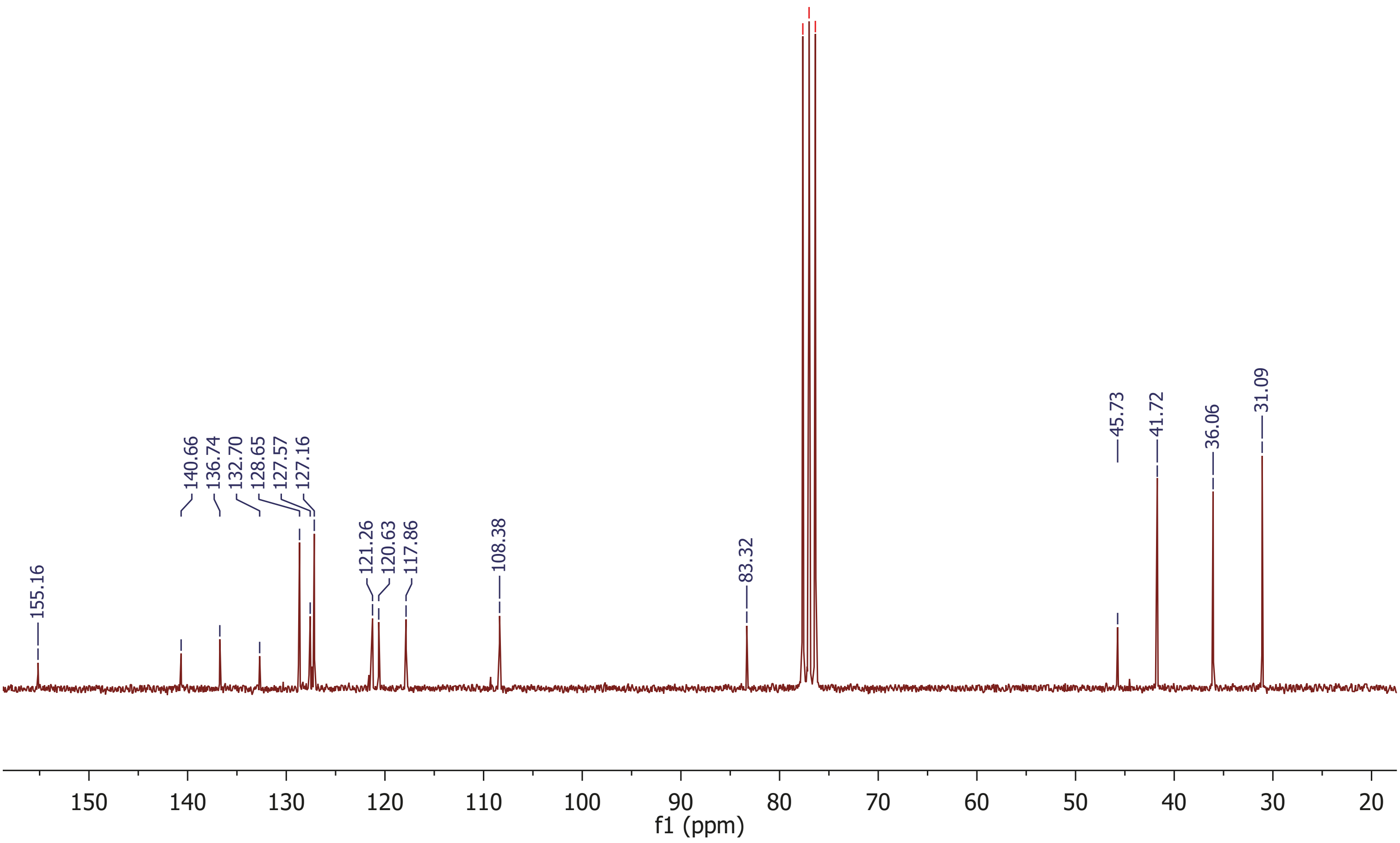
155.16
140.66 136.74 132.70 128.65 127.57 127.16
121.26 120.63 117.86
108.38
83.32
45.73
41.72
36.06
31.09
150 140 130 120 110 100 90 80 70 60 50 40 30 20
f1 (ppm)

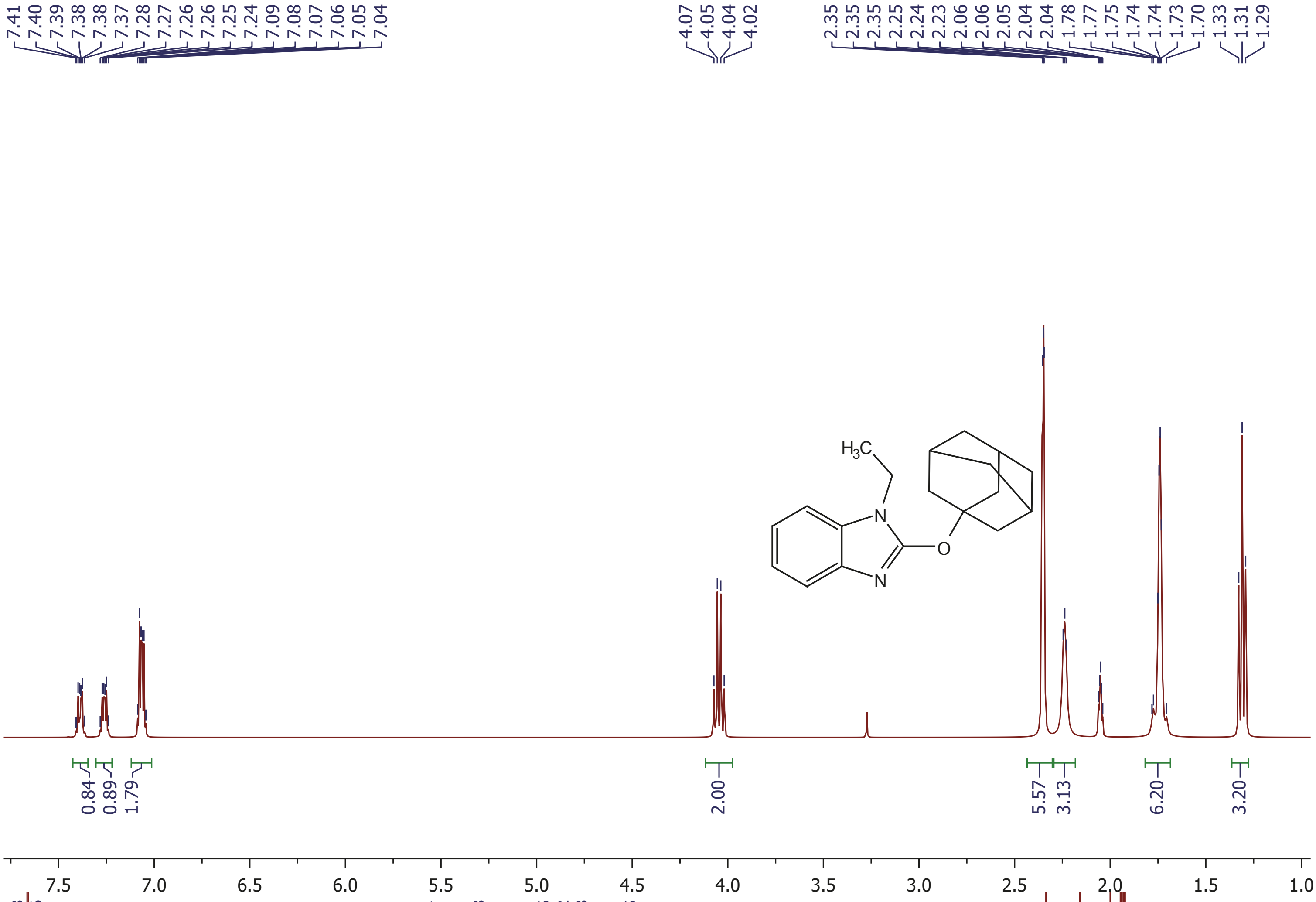
7.41 7.40 7.39 7.38 7.38 7.37 7.28 7.27 7.26 7.26 7.25 7.24 7.09 7.08 7.07 7.06 7.05 7.04
4.07 4.05 4.04 4.02
2.35 2.35 2.35 2.25 2.24 2.23 2.06 2.06 2.05 2.04 2.04 1.78 1.77 1.75 1.74 1.74 1.73 1.70 1.33 1.31 1.29
H_3C
N
N
O
0.84 0.89 1.79 2.00 5.57 3.13 6.20 3.20
7.5 7.0 6.5 6.0 5.5 5.0 4.5 4.0 3.5 3.0 2.5 2.0 1.5 1.0

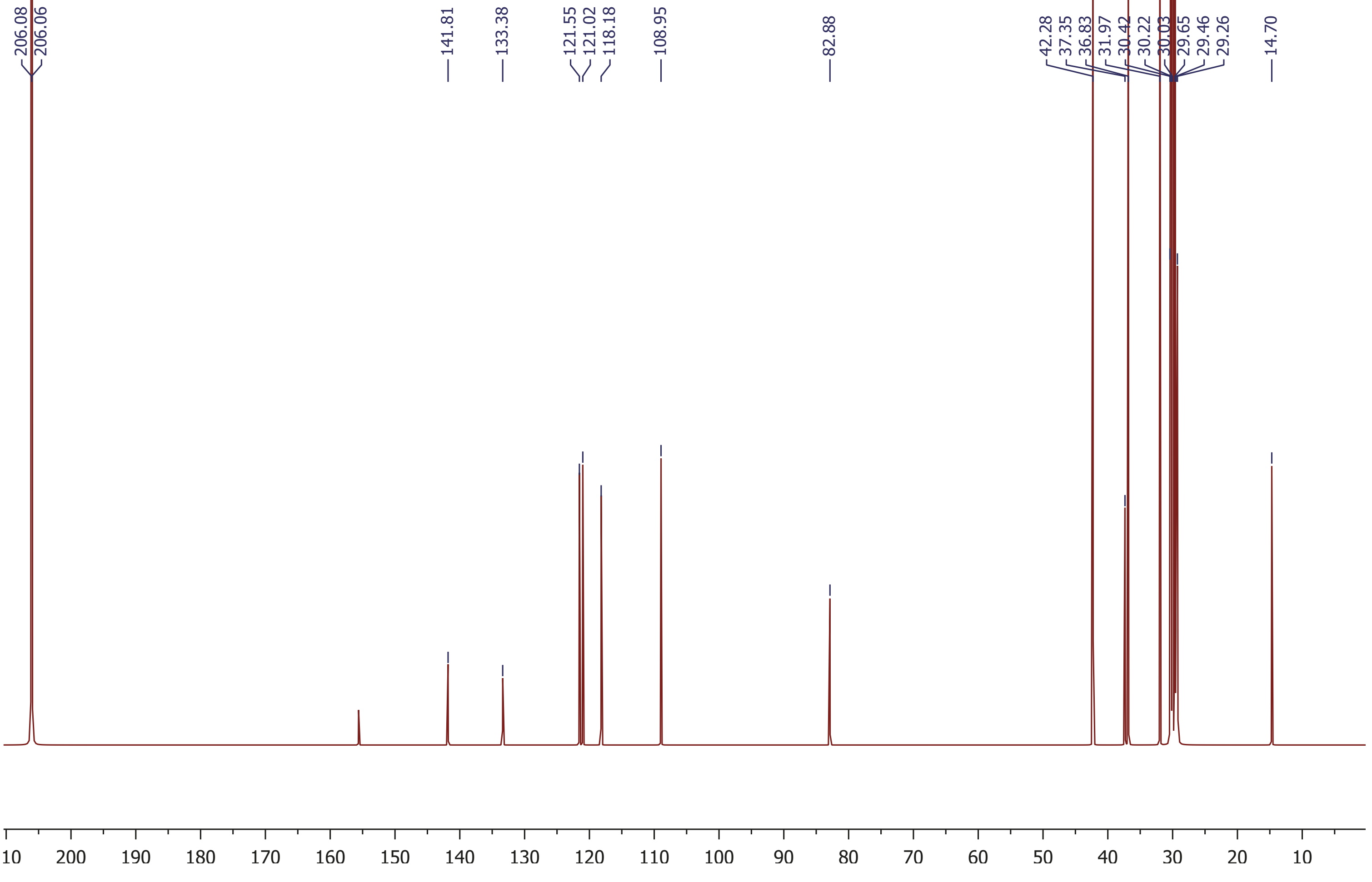
206.08 206.06
141.81
133.38
121.55 121.02 118.18
108.95
82.88
42.28 37.35 36.83 31.97 30.42 30.22 30.03 29.65 29.46 29.26
14.70
10 200 190 180 170 160 150 140 130 120 110 100 90 80 70 60 50 40 30 20 10

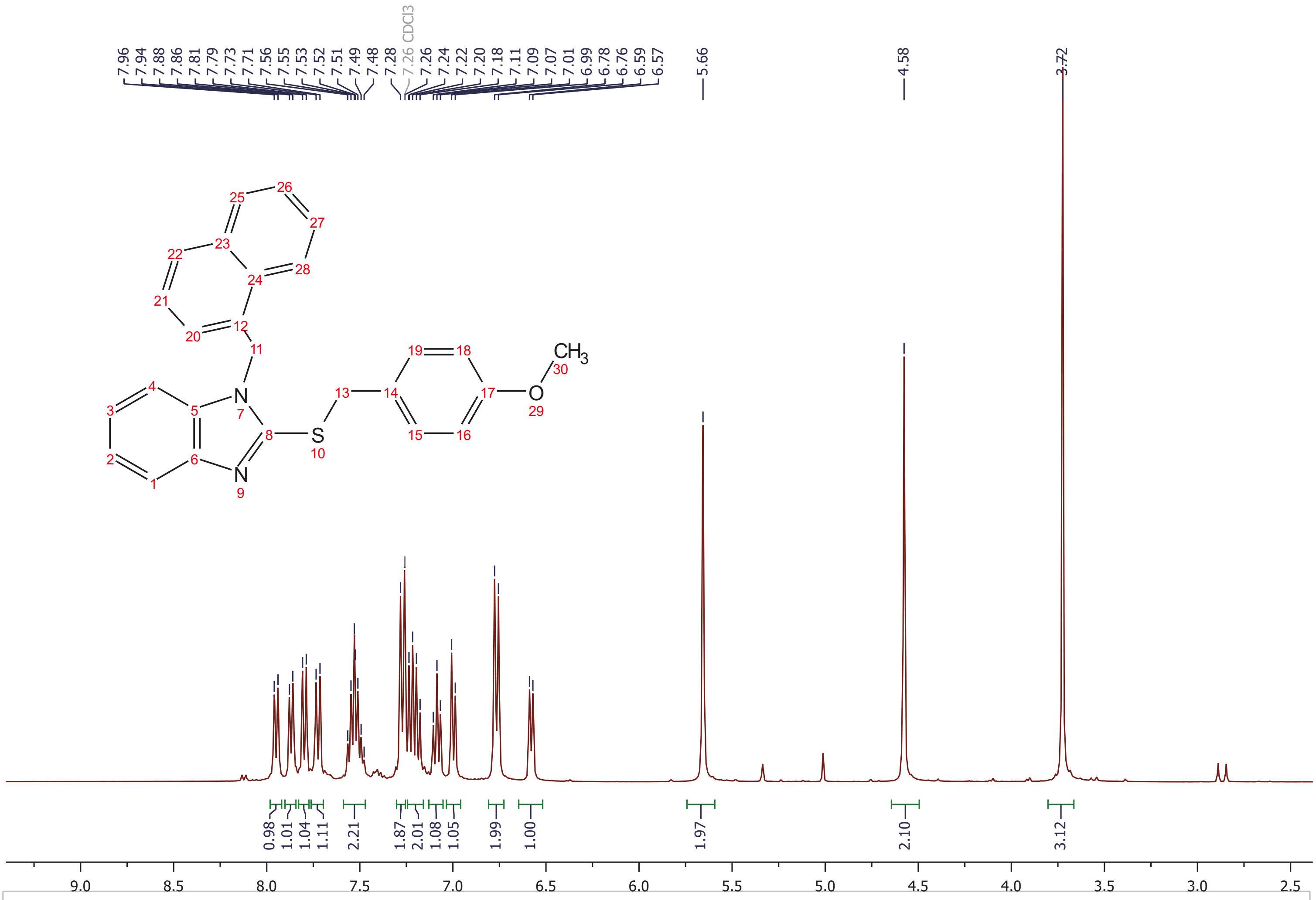
7.96 7.94 7.88 7.86 7.81 7.79 7.73 7.71 7.56 7.55 7.53 7.52 7.51 7.49 7.48 7.28 7.26 CDCl3 7.26 7.24 7.22 7.20 7.18 7.11 7.09 7.07 7.01 6.99 6.78 6.76 6.59 6.57
5.66
4.58
3.72
CH3
N
S
O
N
0.98 1.01 1.04 1.11 2.21 1.87 2.01 1.08 1.05 1.99 1.00 1.97 2.10 3.12
9.0 8.5 8.0 7.5 7.0 6.5 6.0 5.5 5.0 4.5 4.0 3.5 3.0 2.5

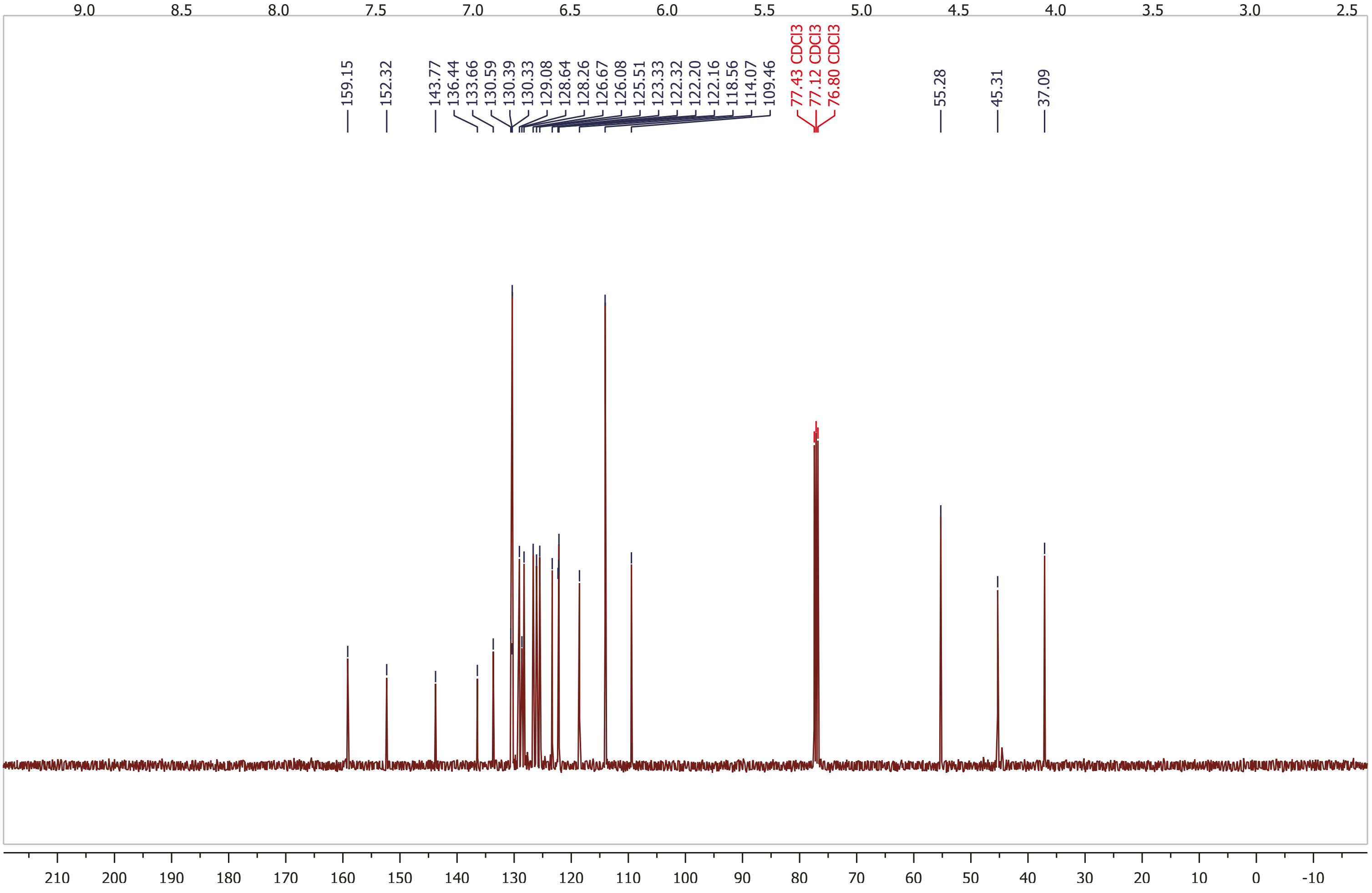
159.15
152.32
143.77 136.44 133.66 130.59 130.39 130.33 129.08 128.64 128.26 126.67 126.08 125.51 123.33 122.32 122.20 122.16 118.56 114.07 109.46
77.43 CDCl3
77.12 CDCl3
76.80 CDCl3
55.28
45.31
37.09
210 200 190 180 170 160 150 140 130 120 110 100 90 80 70 60 50 40 30 20 10 0 -10

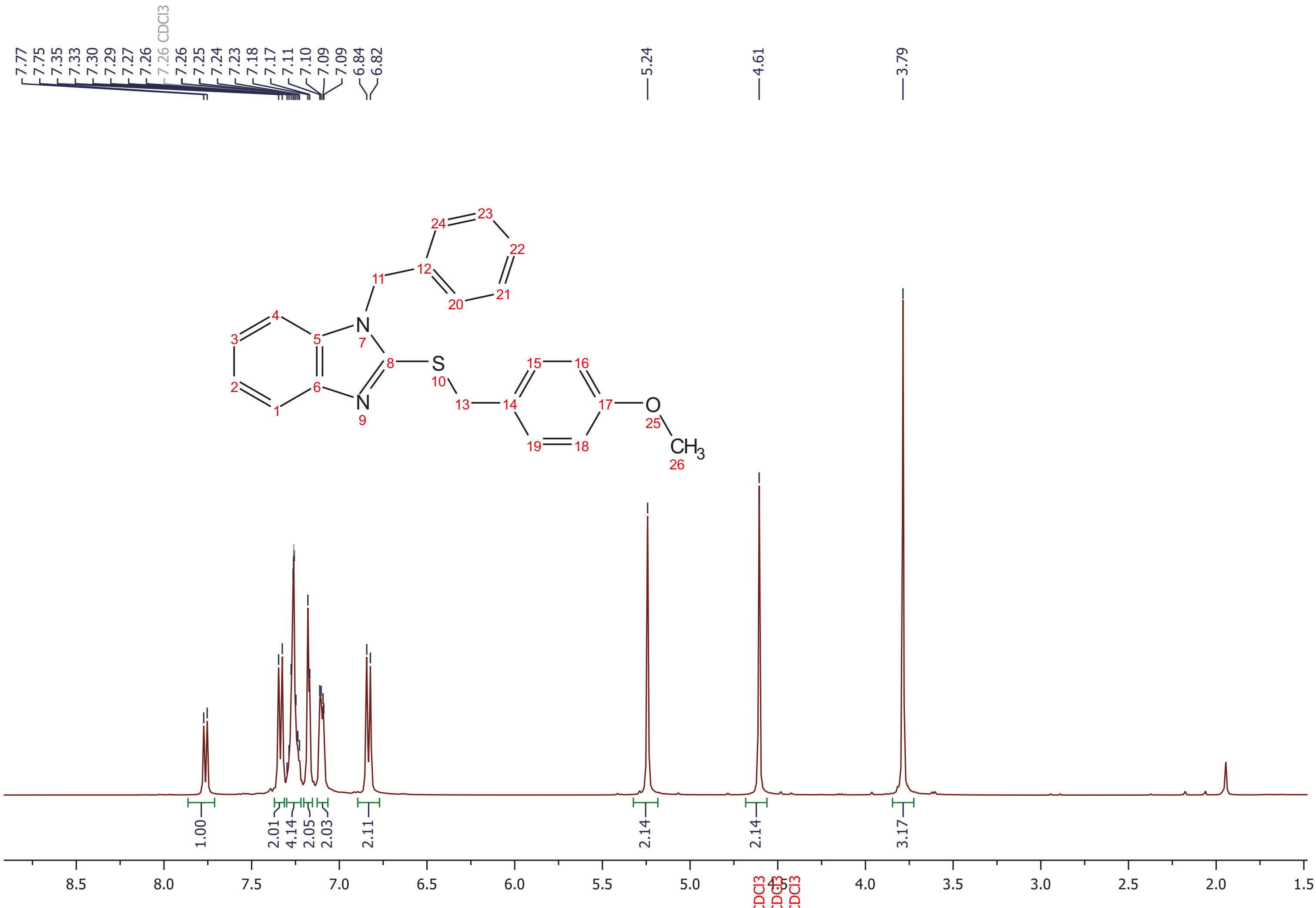
7.77
7.75
7.35
7.33
7.30
7.29
7.27
7.26
7.26 CDCl3
7.26
7.25
7.24
7.23
7.18
7.17
7.11
7.10
7.09
7.09
6.84
6.82
5.24
4.61
3.79
1.00
2.01
4.14
2.05
2.03
2.11
2.14
2.14
3.17
N
S
N
O
CH3
8.5
8.0
7.5
7.0
6.5
6.0
5.5
5.0
4.5
4.0
3.5
3.0
2.5
2.0
1.5

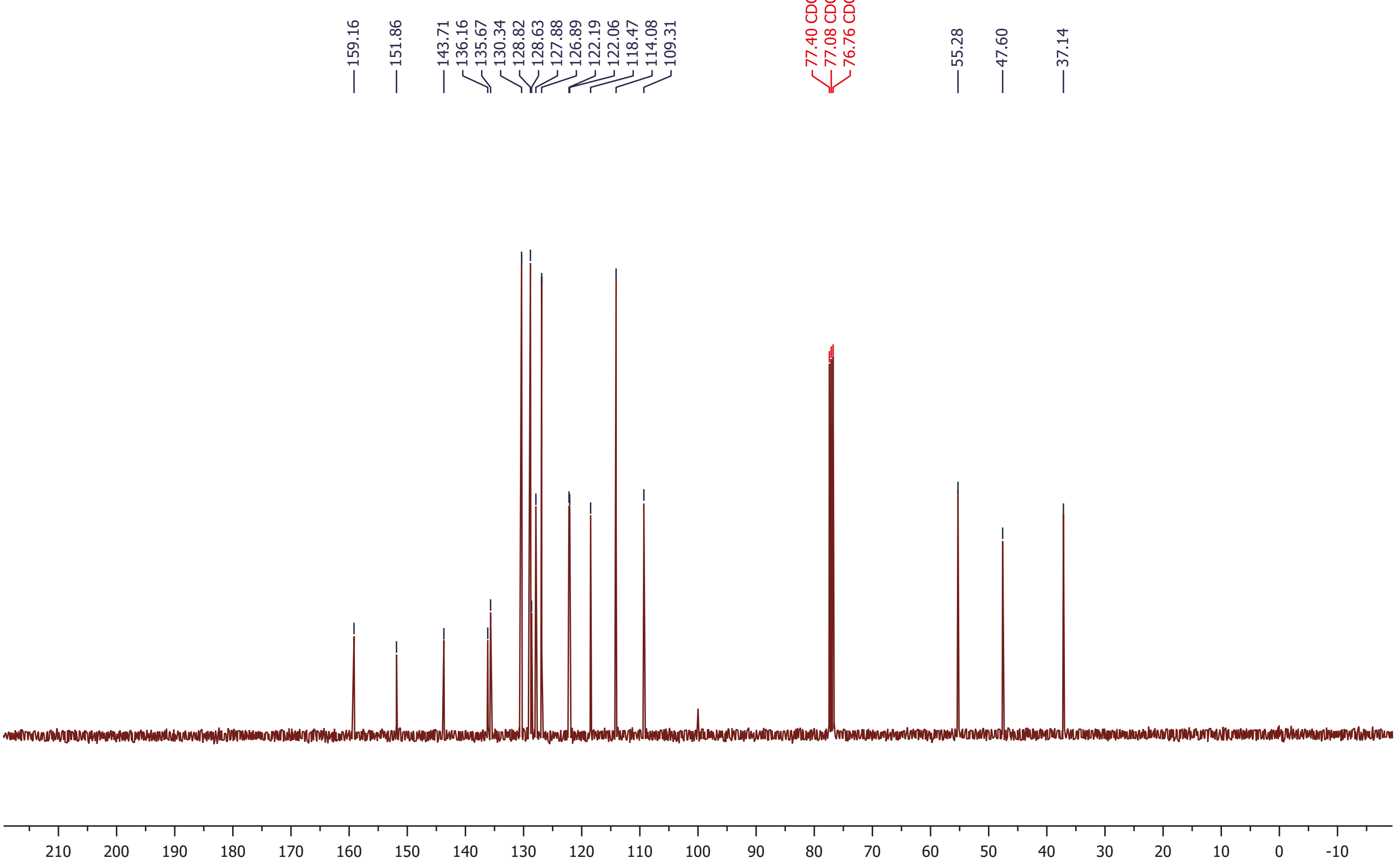
159.16
151.86
143.71
136.16
135.67
130.34
128.82
128.63
127.88
126.89
122.19
122.06
118.47
114.08
109.31
77.40 CDCl3
77.08 CDCl3
76.76 CDCl3
55.28
47.60
37.14
210
200
190
180
170
160
150
140
130
120
110
100
90
80
70
60
50
40
30
20
10
0
-10

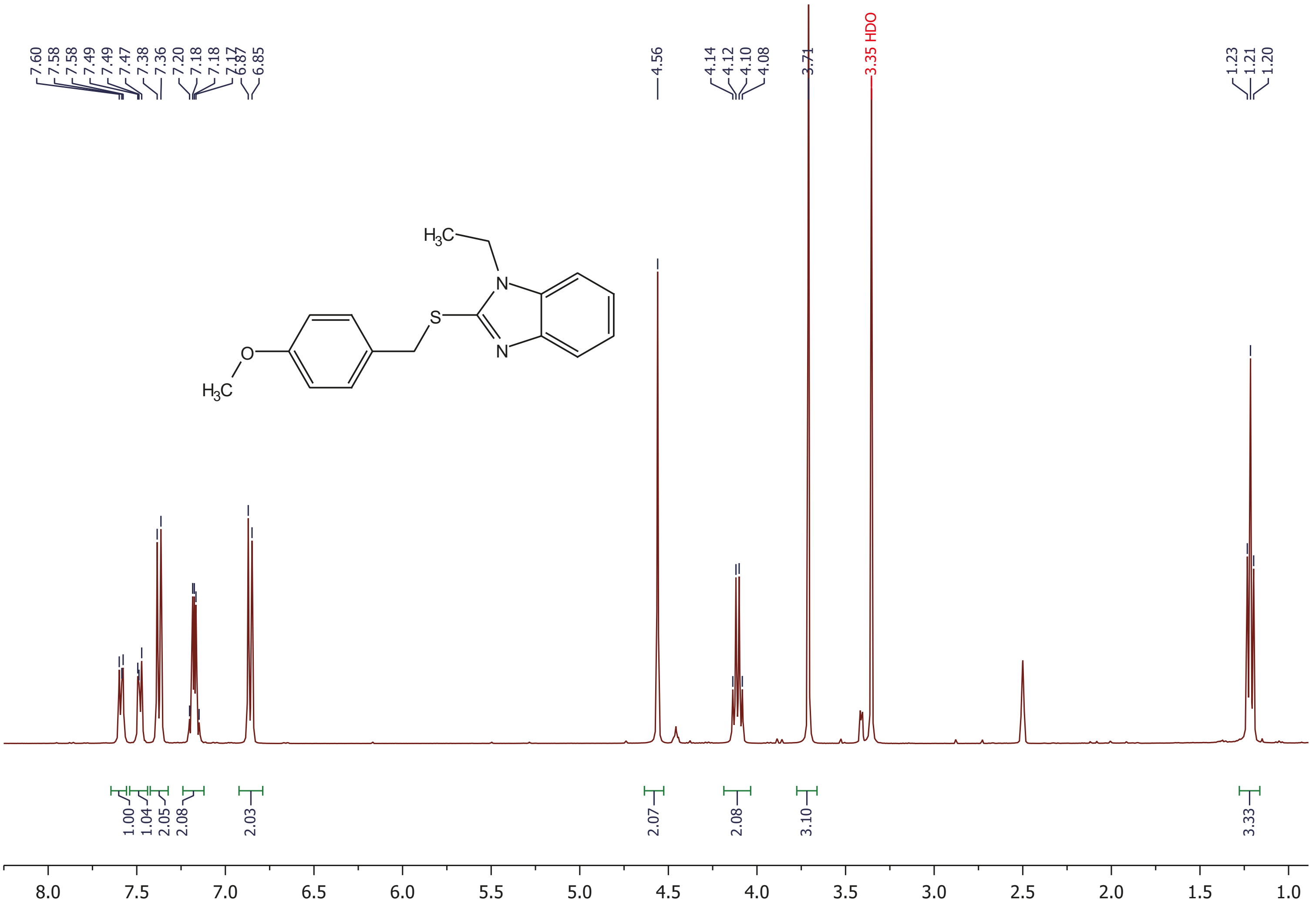
7.60
7.58
7.58
7.49
7.49
7.47
7.38
7.36
7.20
7.18
7.18
7.17
6.87
6.85
4.56
4.14
4.12
4.10
4.08
3.71
3.35 HDO
1.23
1.21
1.20
H_3C
N
S
N
O
H_3C
1.00
1.04
2.05
2.08
2.03
2.07
2.08
3.10
3.33
8.0
7.5
7.0
6.5
6.0
5.5
5.0
4.5
4.0
3.5
3.0
2.5
2.0
1.5
1.0

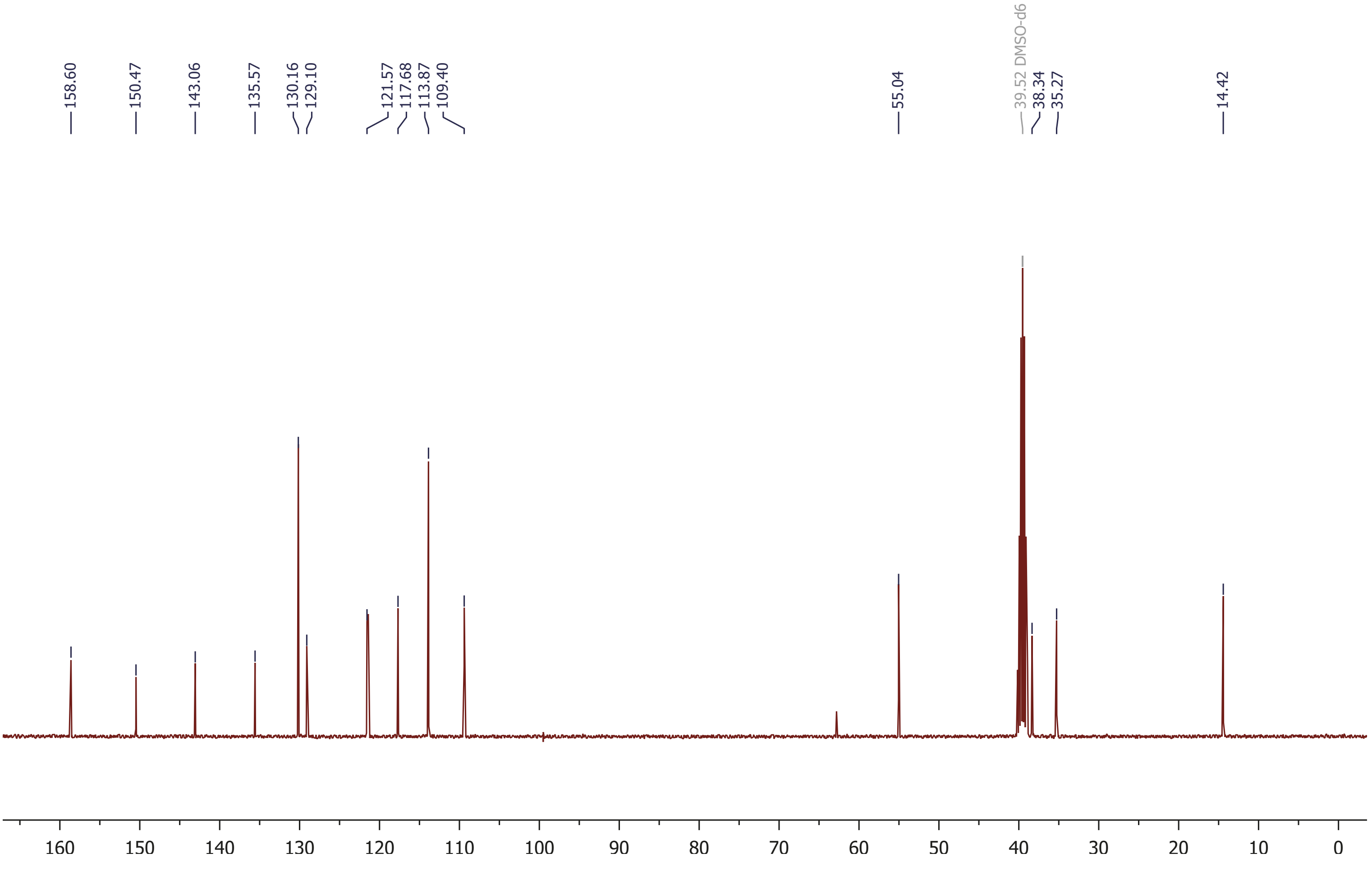
158.60
150.47
143.06
135.57
130.16
129.10
121.57
117.68
113.87
109.40
55.04
39.52 DMSO-d6
38.34
35.27
14.42
160
150
140
130
120
110
100
90
80
70
60
50
40
30
20
10
0

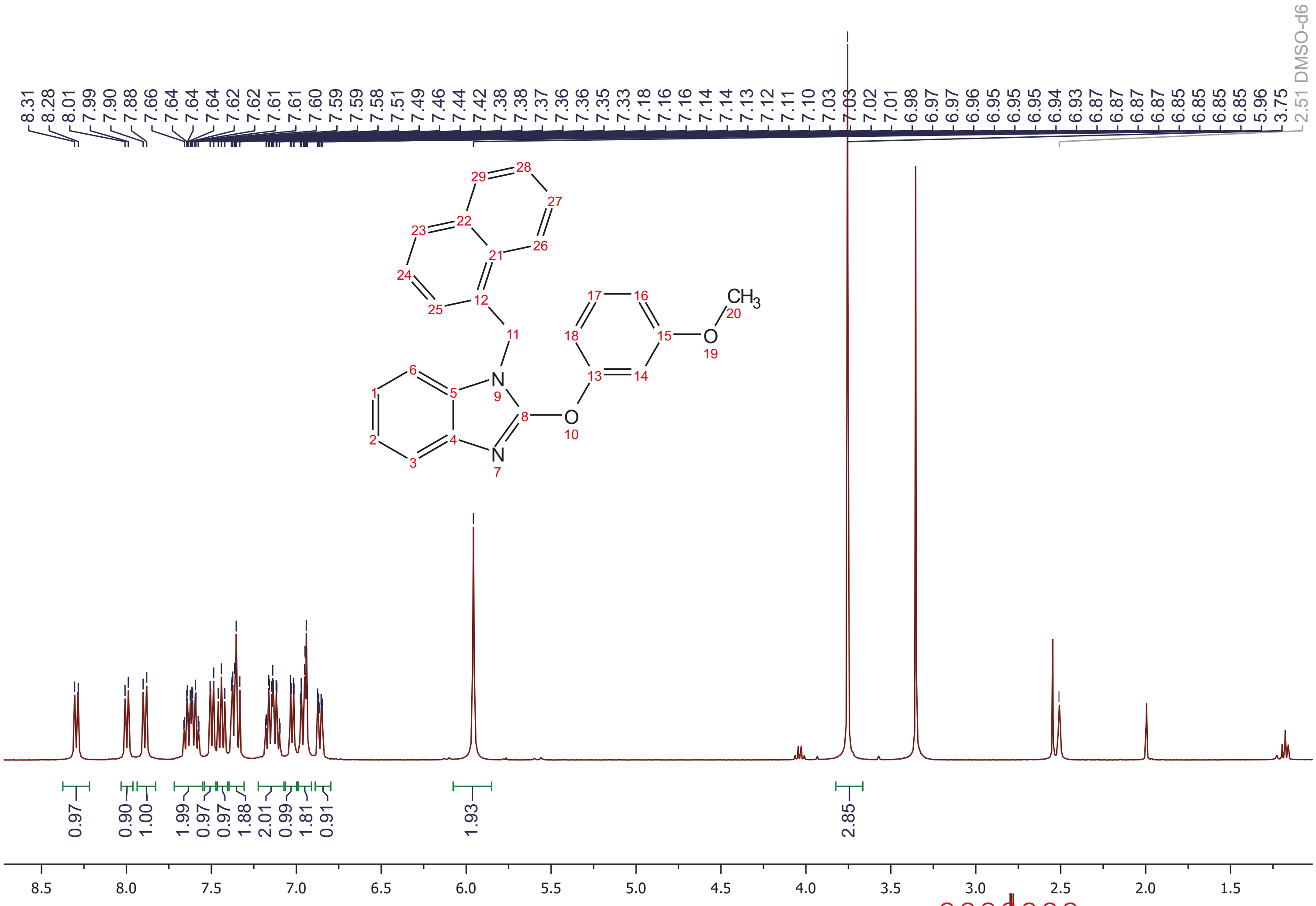
8.31 8.28 8.01 7.99 7.90 7.88 7.66 7.64 7.64 7.64 7.62 7.62 7.61 7.61 7.60 7.59 7.59 7.58 7.51 7.49 7.46 7.44 7.42 7.38 7.38 7.37 7.36 7.36 7.35 7.33 7.18 7.16 7.16 7.14 7.14 7.13 7.12 7.11 7.10 7.03 7.03 7.02 7.01 6.98 6.97 6.97 6.96 6.95 6.95 6.95 6.94 6.93 6.87 6.87 6.87 6.85 6.85 6.85 6.85 5.96 3.75
2.51 DMSO-d6
CH3
0.97 0.90 1.00 1.99 0.97 0.97 1.88 2.01 0.99 1.81 0.91 1.93 2.85
8.5 8.0 7.5 7.0 6.5 6.0 5.5 5.0 4.5 4.0 3.5 3.0 2.5 2.0 1.5
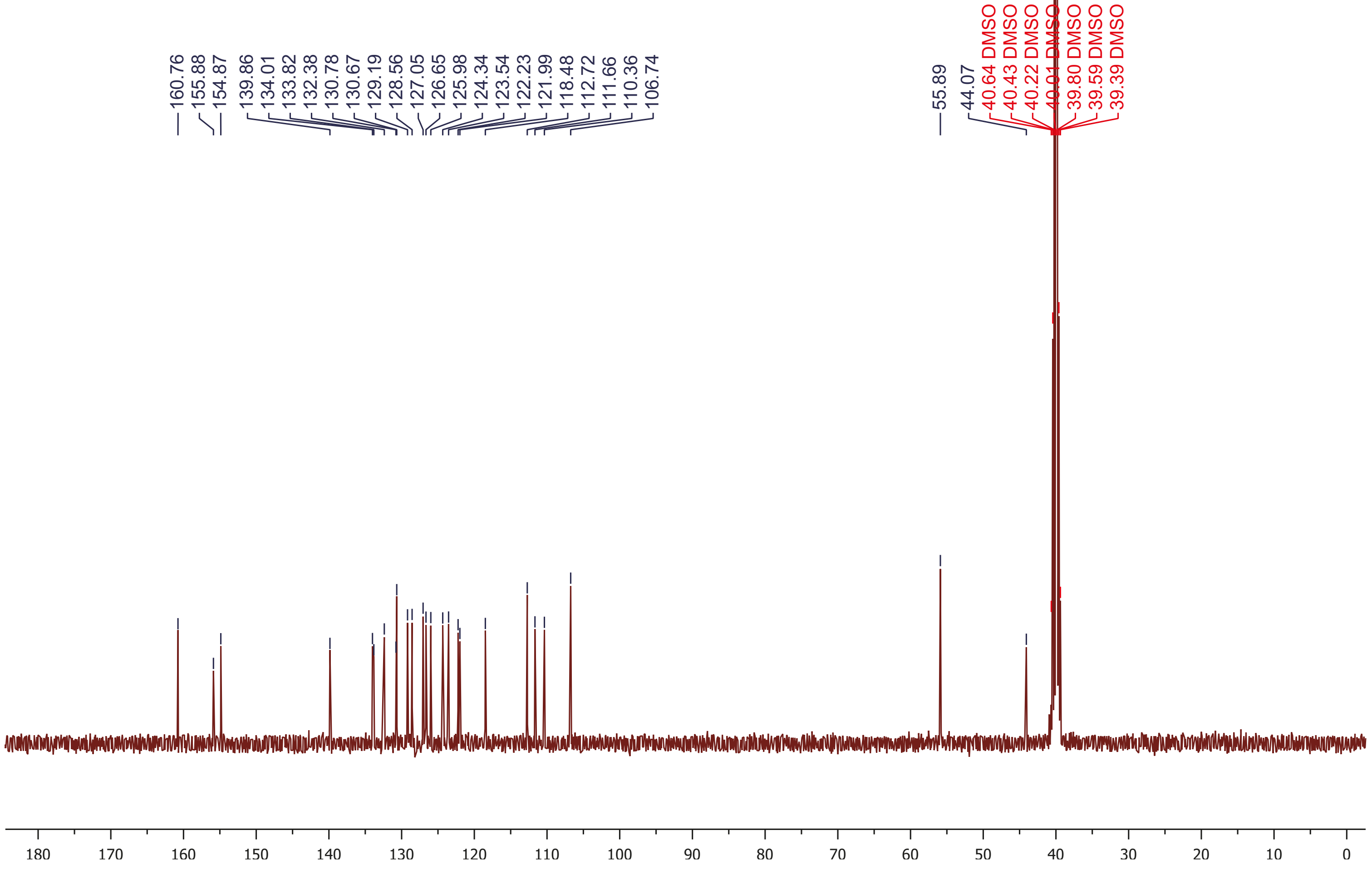
160.76 155.88 154.87 139.86 134.01 133.82 132.38 130.78 130.67 129.19 128.56 127.05 126.65 125.98 124.34 123.54 122.23 121.99 118.48 112.72 111.66 110.36 106.74
55.89 44.07
40.64 DMSO 40.43 DMSO 40.22 DMSO 40.01 DMSO 39.80 DMSO 39.59 DMSO 39.39 DMSO
180 170 160 150 140 130 120 110 100 90 80 70 60 50 40 30 20 10 0

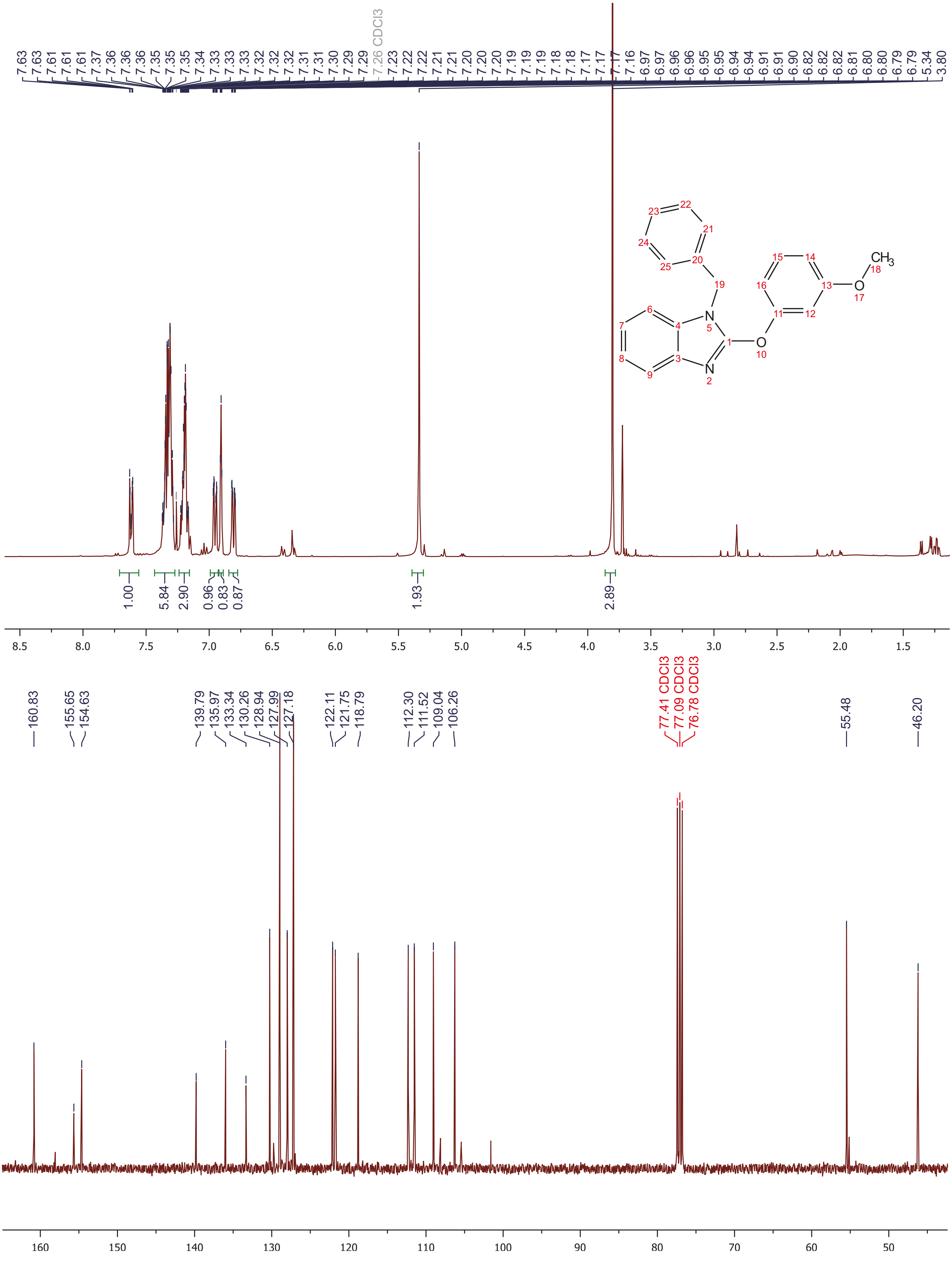

7.26 CDCl3
1.00
5.84
2.90
0.96
0.83
0.87
1.93
2.89
160.83
155.65
154.63
139.79
135.97
133.34
130.26
128.94
127.99
127.18
122.11
121.75
118.79
112.30
111.52
109.04
106.26
77.41 CDCl3
77.09 CDCl3
76.78 CDCl3
55.48
46.20

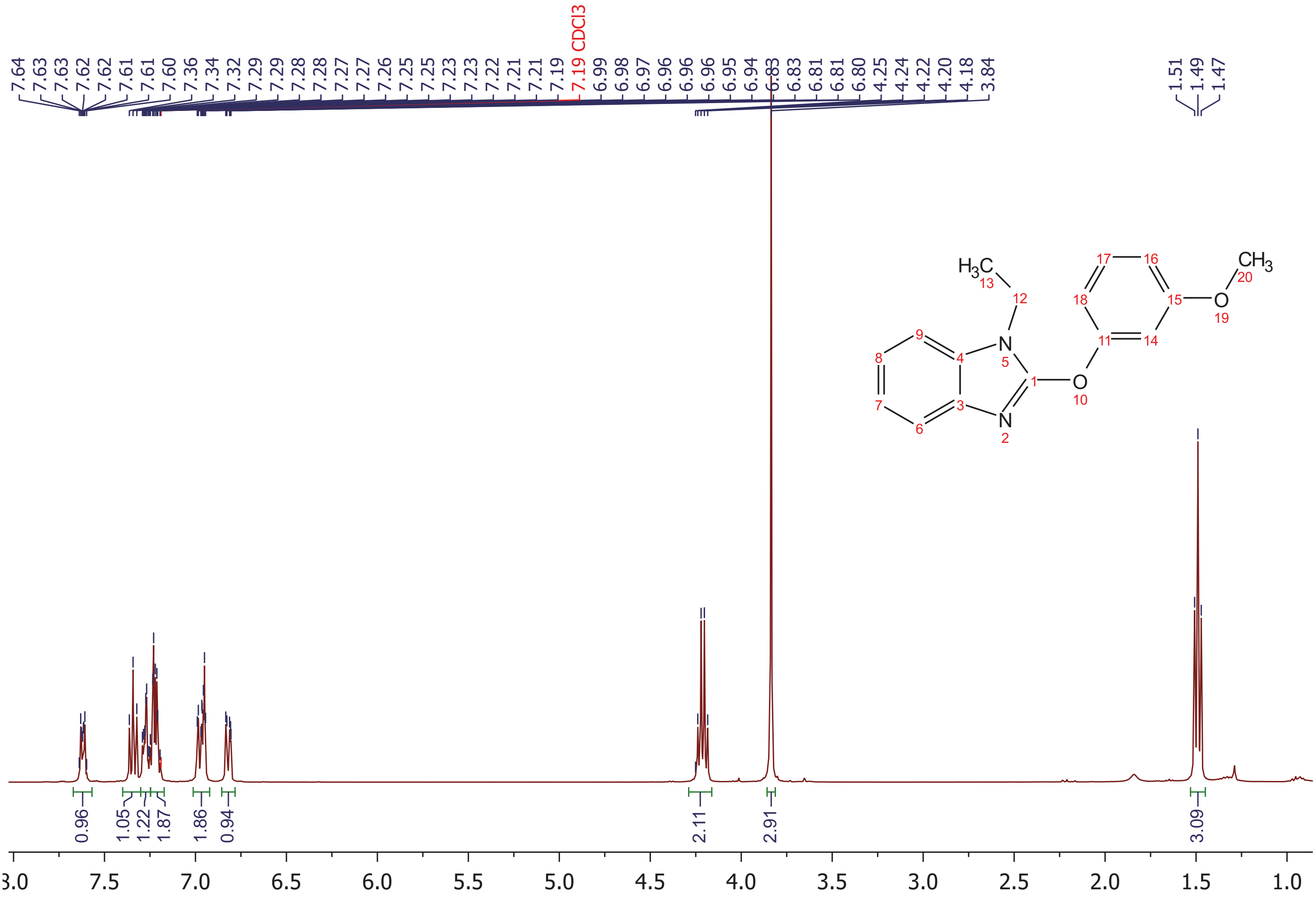

7.64
7.63
7.63
7.62
7.62
7.61
7.61
7.60
7.36
7.34
7.32
7.29
7.29
7.28
7.28
7.27
7.27
7.26
7.25
7.25
7.23
7.23
7.22
7.21
7.21
7.19
7.19 CDCl3
6.99
6.98
6.97
6.96
6.96
6.96
6.95
6.94
6.83
6.83
6.81
6.81
6.80
4.25
4.24
4.22
4.20
4.18
3.84
1.51
1.49
1.47
0.96
1.05
1.22
1.87
1.86
0.94
2.11
2.91
3.09
H3C
13
12
N
5
O
10
N
2
1
3
4
6
7
8
9
11
14
15
16
17
18
O
19
CH3
20

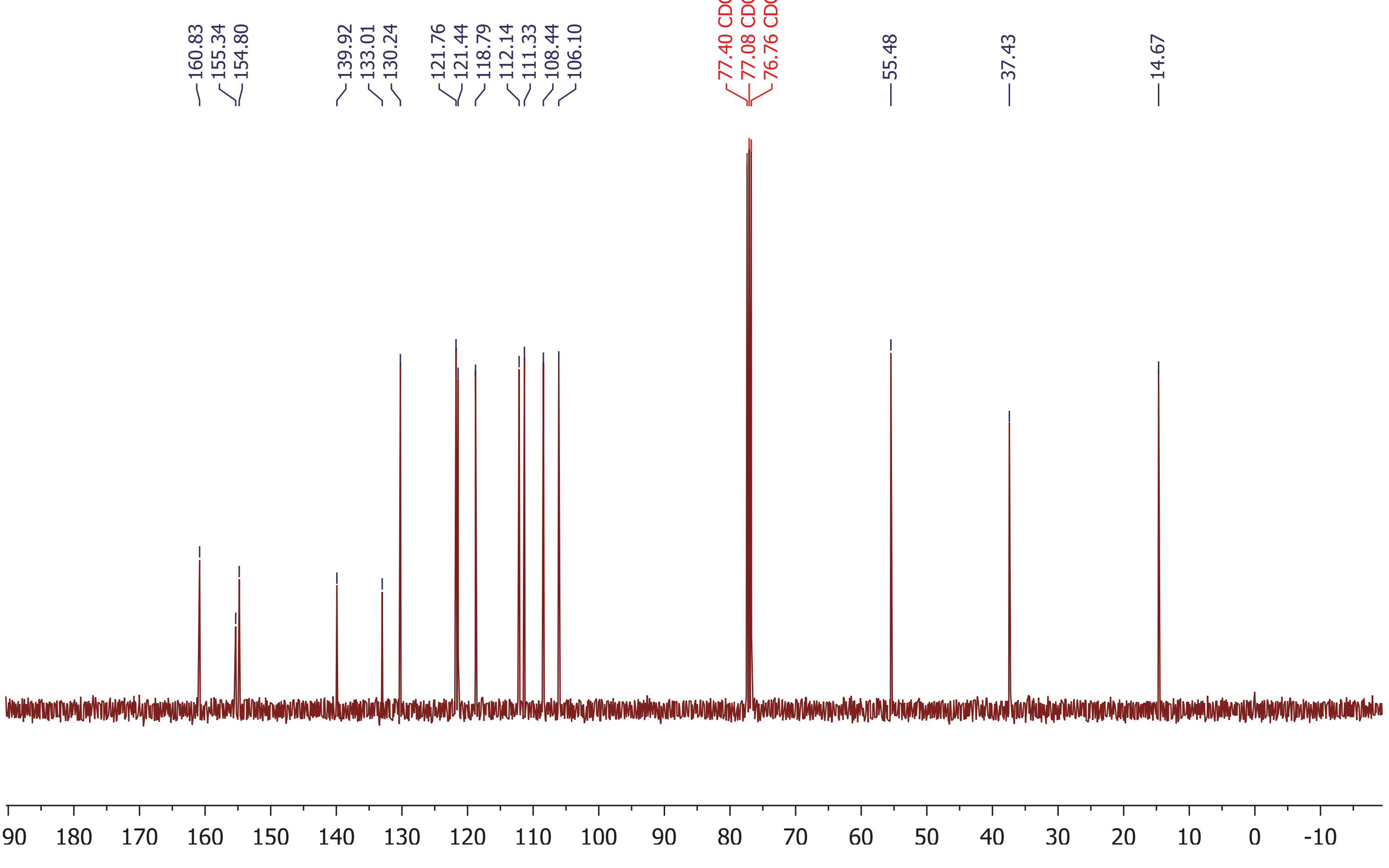

160.83
155.34
154.80
139.92
133.01
130.24
121.76
121.44
118.79
112.14
111.33
108.44
106.10
77.40 CDCl3
77.08 CDCl3
76.76 CDCl3
55.48
37.43
14.67

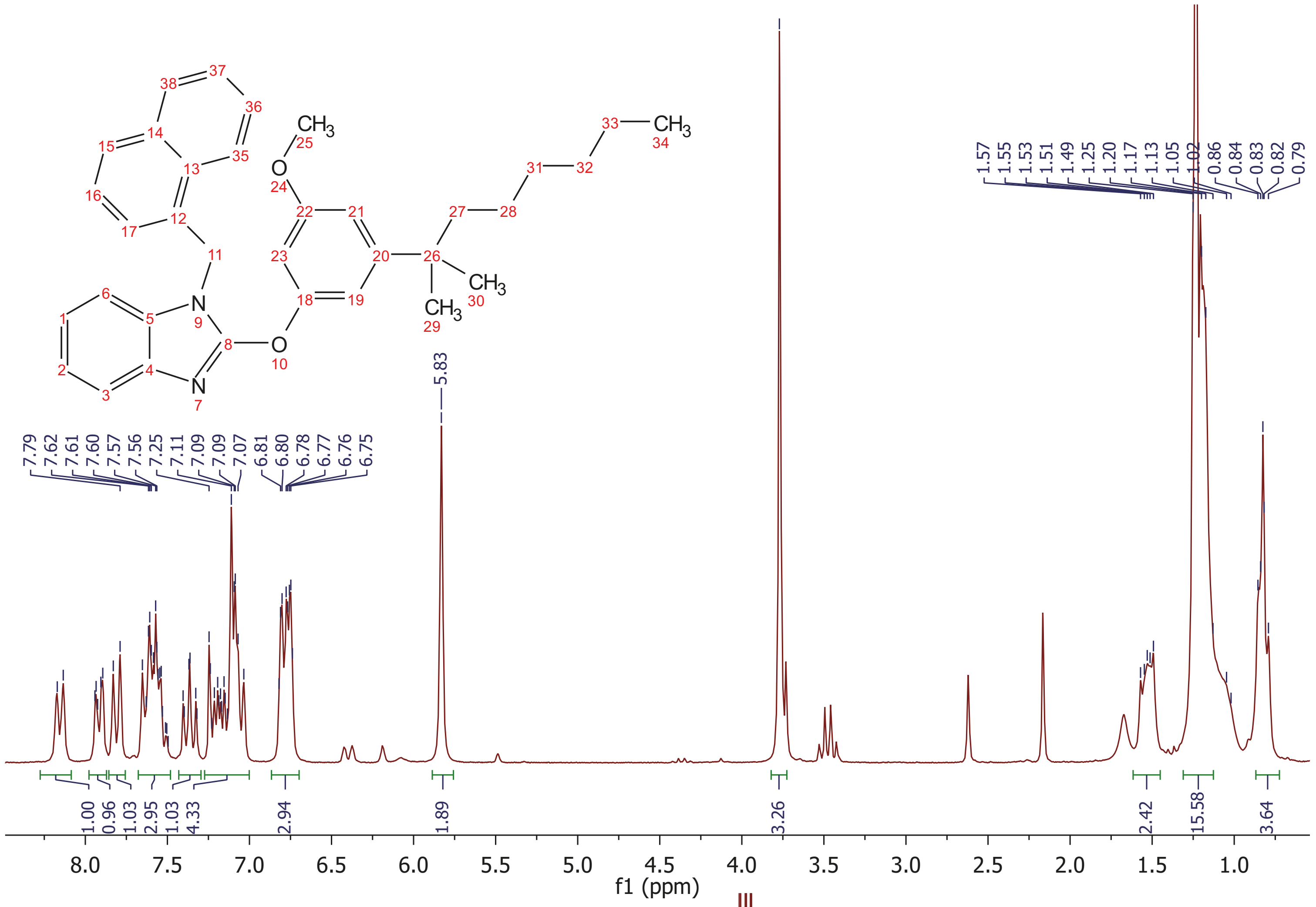

7.79
7.62
7.61
7.60
7.57
7.56
7.25
7.11
7.09
7.09
7.07
6.81
6.80
6.78
6.77
6.76
6.75
5.83
1.57
1.55
1.53
1.51
1.49
1.25
1.20
1.17
1.13
1.05
1.02
0.86
0.84
0.83
0.82
0.79
1.00
0.96
1.03
2.95
1.03
4.33
2.94
1.89
3.26
2.42
15.58
3.64
f1 (ppm)

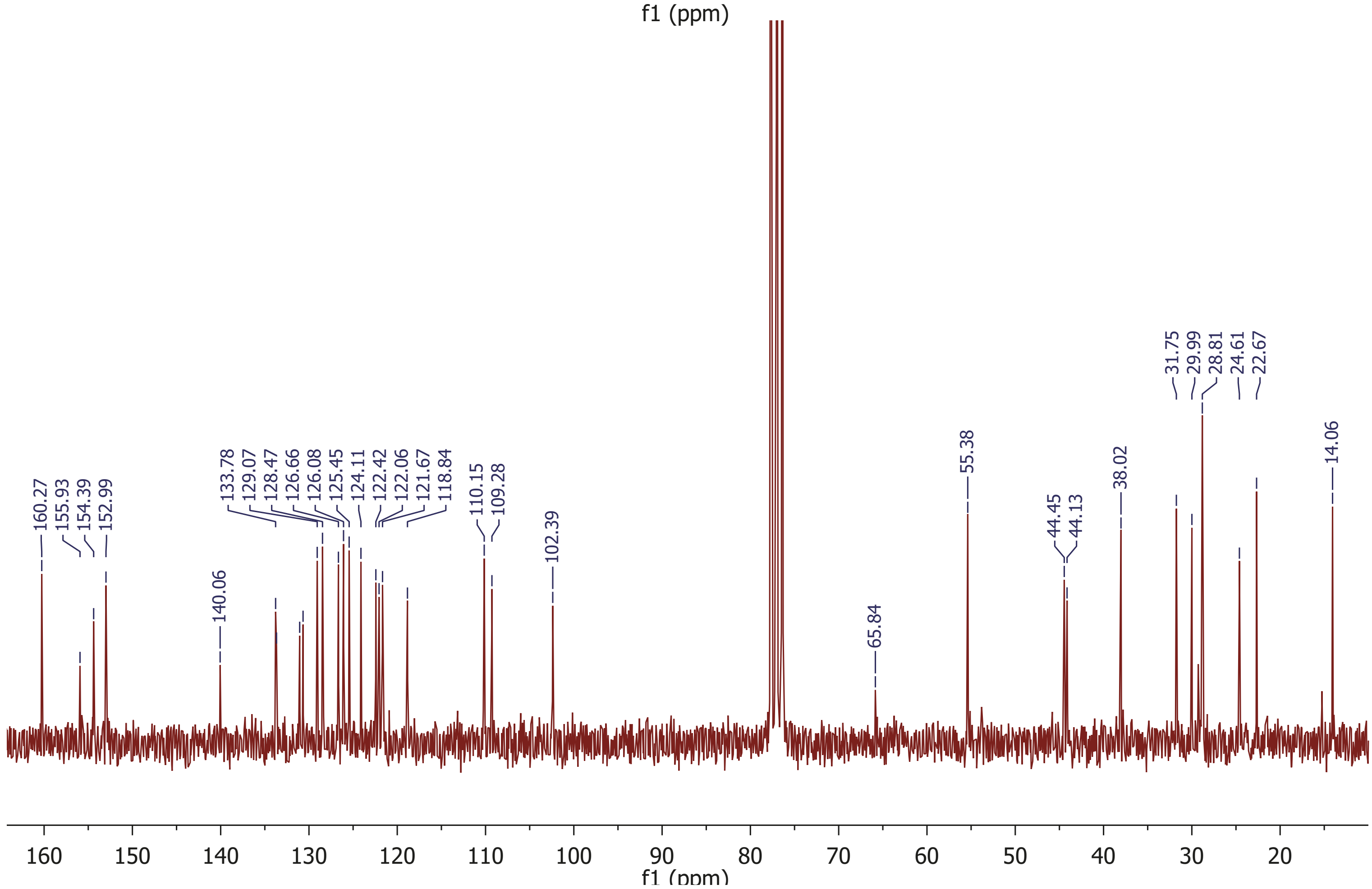

160.27
155.93
154.39
152.99
140.06
133.78
129.07
128.47
126.66
126.08
125.45
124.11
122.42
122.06
121.67
118.84
110.15
109.28
102.39
65.84
55.38
44.45
44.13
38.02
31.75
29.99
28.81
24.61
22.67
14.06
f1 (ppm)

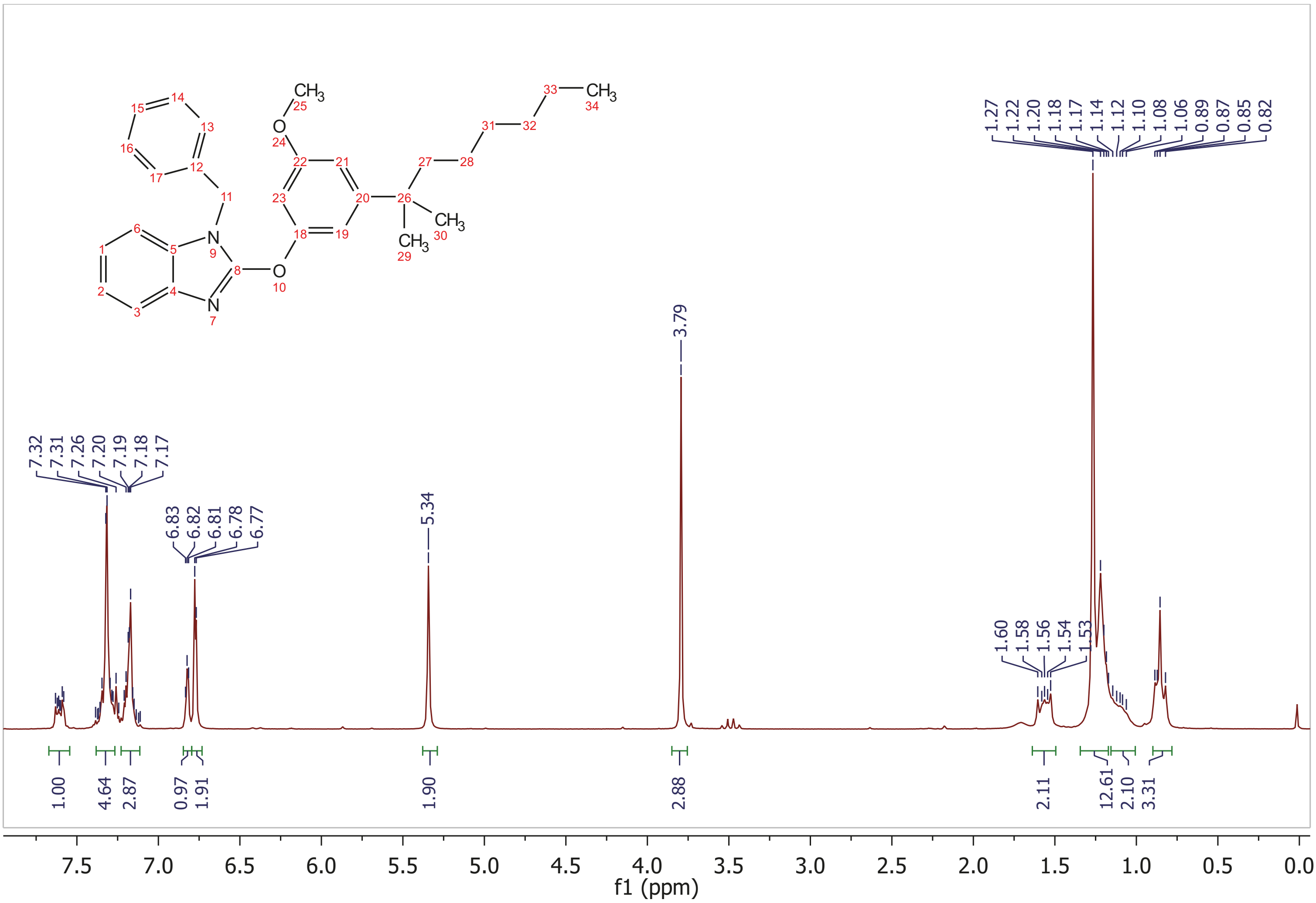
7.32 7.31 7.26 7.20 7.19 7.18 7.17
6.83 6.82 6.81 6.78 6.77
5.34
3.79
1.60 1.58 1.56 1.54 1.53
1.27 1.22 1.20 1.18 1.17 1.14 1.12 1.10 1.08 1.06 0.89 0.87 0.85 0.82
1.00 4.64 2.87 0.97 1.91 1.90 2.88 2.11 12.61 2.10 3.31
7.5 7.0 6.5 6.0 5.5 5.0 4.5 4.0 3.5 3.0 2.5 2.0 1.5 1.0 0.5 0.0
f1 (ppm)

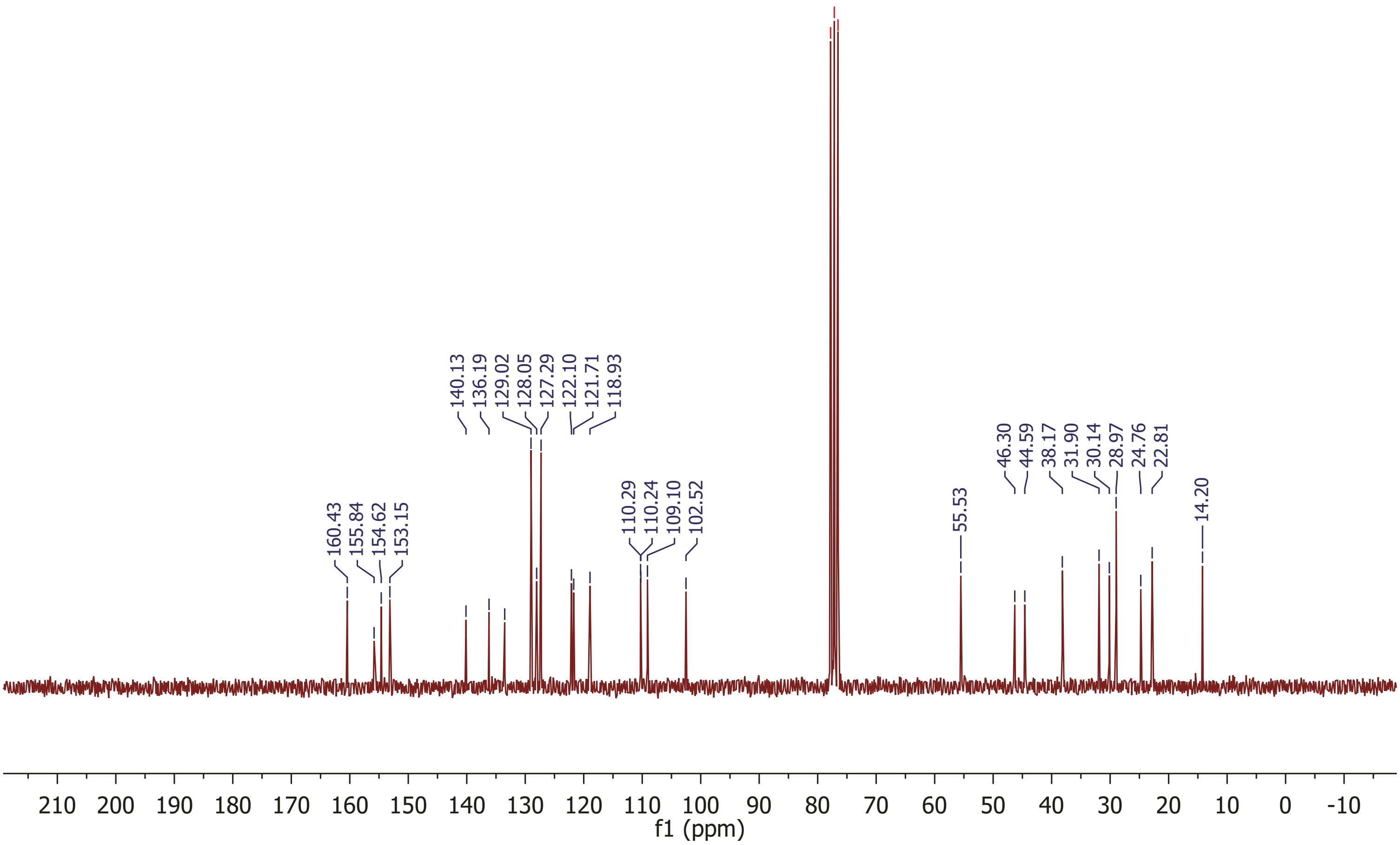
160.43 155.84 154.62 153.15
140.13 136.19 129.02 128.05 127.29 122.10 121.71 118.93
110.29 110.24 109.10 102.52
55.53
46.30 44.59 38.17 31.90 30.14 28.97 24.76 22.81
14.20
210 200 190 180 170 160 150 140 130 120 110 100 90 80 70 60 50 40 30 20 10 0 -10
f1 (ppm)

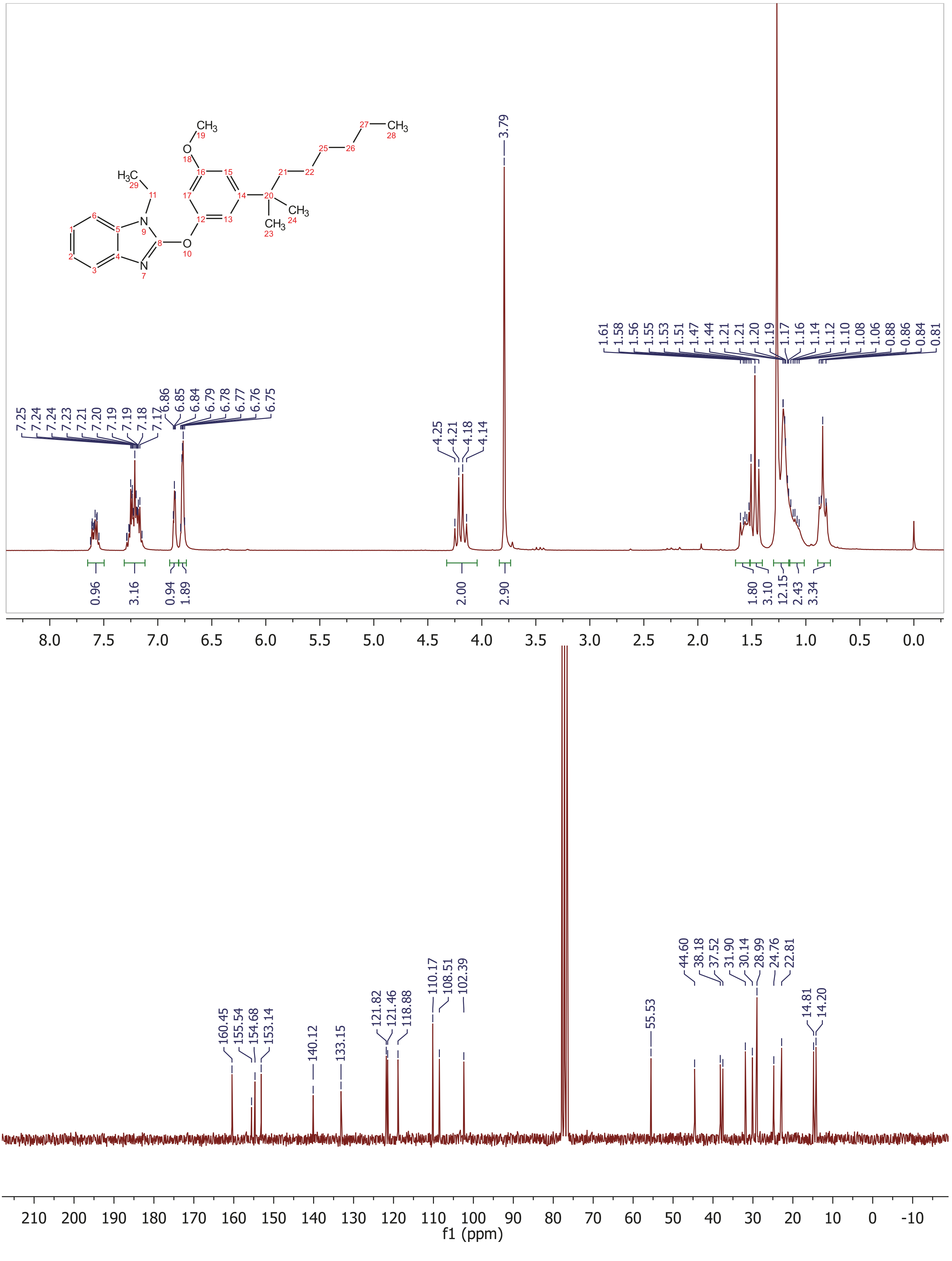

3.79
4.25
4.21
4.18
4.14
7.25
7.24
7.24
7.23
7.21
7.20
7.19
7.19
7.18
7.17
6.86
6.85
6.84
6.79
6.78
6.77
6.76
6.75
1.61
1.58
1.56
1.55
1.53
1.51
1.47
1.44
1.21
1.21
1.20
1.19
1.17
1.16
1.14
1.12
1.10
1.08
1.06
0.88
0.86
0.84
0.81
0.96
3.16
0.94
1.89
2.00
2.90
1.80
3.10
12.15
2.43
3.34
160.45
155.54
154.68
153.14
140.12
133.15
121.82
121.46
118.88
110.17
108.51
102.39
55.53
44.60
38.18
37.52
31.90
30.14
28.99
24.76
22.81
14.81
14.20
f1 (ppm)
